# A Molecular Ruler for Measuring Quantitative Distance Distributions

**DOI:** 10.1371/journal.pone.0003229

**Published:** 2008-10-17

**Authors:** Rebecca S. Mathew-Fenn, Rhiju Das, Joshua A. Silverman, Peter A. Walker, Pehr A. B. Harbury

**Affiliations:** 1 Department of Biochemistry, Stanford University, Stanford, California, United States of America; 2 Biophysics Program, Stanford University, Stanford, California, United States of America; 3 Department of Physics, Stanford University, Stanford, California, United States of America; The University of Manchester, United Kingdom

## Abstract

We report a novel molecular ruler for measurement of distances and distance distributions with accurate external calibration. Using solution X-ray scattering we determine the scattering interference between two gold nanocrystal probes attached site-specifically to a macromolecule of interest. Fourier transformation of the interference pattern provides a model-independent probability distribution for the distances between the probe centers-of-mass. To test the approach, we measure end-to-end distances for a variety of DNA structures. We demonstrate that measurements with independently prepared samples and using different X-ray sources are highly reproducible, we demonstrate the quantitative accuracy of the first and second moments of the distance distributions, and we demonstrate that the technique recovers complex distribution shapes. Distances measured with the solution scattering-interference ruler match the corresponding crystallographic values, but differ from distances measured previously with alternate ruler techniques. The X-ray scattering interference ruler should be a powerful tool for relating crystal structures to solution structures and for studying molecular fluctuations.

## Introduction

Molecular rulers comprise a family of experimental tools that provide qualitative intramolecular distance measurements for macromolecules in free solution. They have played a central role in the study of nanometer-scale conformational change. However, existing molecular ruler techniques suffer from two serious limitations: they do not provide quantitatively accurate distance values and they cannot accurately determine distributions composed of multiple different distances. Exact distances and distance distributions are desirable when solution measurements must be compared to crystal structures, generally to distinguish between models of how a particular protein or nucleic acid functions. The limitations of existing molecular rulers arise from the physical principle on which they are based, dipolar coupling between two probes incorporated site-specifically into a macromolecule. Uncertainties in the relative orientation and dynamics of the probe dipoles necessarily lead to uncertainties in the distances that are measured, so that the distances are not calibrated and must be interpreted qualitatively [1].

An alternative physical phenomenon on which to base a molecular ruler is solution X-ray scattering. Currently, solution scattering is primarily used to obtain the rough overall shape of macromolecules. In principle, point-to-point distance measurements could be obtained if a macromolecule were labeled at two locations with strongly scattering probes [2]–[4]. The scattering intensity profile of the double-labeled macromolecule, *I_AB_(S)*, would include the probe-to-probe scattering interference pattern, *I_Δ_(S)*. This interference pattern determines the distribution of distances between the probe centers-of-mass, *P_Δ_(D)*, according to the Debye formula [5]:
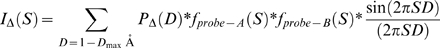
where *f_probe_(S)* is the scattering form factor for the probe. The distance distribution function *P_Δ_(D)* could thus be determined directly by decomposition of *I_Δ_(S)* into a linear sum of the appropriate sinusoidal basis functions. One complication is that the scattering intensity profile of the double-labeled macromolecule includes signals other than the inter-probe scattering interference pattern, specifically the intra-probe, intra-macromolecule, and probe-macromolecule interference patterns. Fortunately, these additional signals can be determined from the scattering profiles of the isolated probe, the isolated macromolecule and the single-labeled macromolecule, and then subtracted off [4]. Importantly, extraction of distances from diffraction data is not subject to the ambiguities associated with current molecular ruler techniques (see Discussion).

Attempts to implement such a scattering interference ruler over the last thirty years have met with partial success. Vainshtein and colleagues attached mercarbide clusters (4 mercury atoms) to cysteine residues in the two β-chains of met-form hemoglobin and measured a separation distance of 37.6 Å between the probes by X-ray scattering interference, in good agreement with the 38 Å distance derived from crystal structures [6]. Miake-Lye and colleagues replaced two calcium ions in rabbit parvalbumin with terbium ions and measured an anomalous X-ray scattering interference signal between the terbium ions and the protein center-of-mass [7]. Finally, Moore, Engelman, and colleagues used neutron scattering interference between pairs of deuterated ribosomal proteins to measure average inter-protein distances within the ribosome [8]. Their early structural model of the small ribosomal subunit model was largely validated by subsequent high-resolution crystal structures [9]. In all of these examples, the investigators could only measure an average distance between the scatterers. Distance distributions were not obtainable because of inadequate signal over noise in the data.

Here we describe a molecular ruler that utilizes 14-Å gold nanocrystal probes. The nanocrystals are attached to the 3′-ends of DNA duplexes. We measure the pattern of X-ray scattering interference between the nanocrystals and transform it into the point-to-point distance probability distribution between their centers-of-mass. We demonstrate that measurements with independently prepared samples and using different X-ray sources are extremely reproducible, we demonstrate the quantitative accuracy of the first and second moments of the distance distributions, and we demonstrate that the technique recovers complex distribution shapes that have been difficult or impossible to obtain previously.

## Materials and Methods

### Synthesis and purification of nanocrystals

Synthesis of water-soluble gold nanocrystals followed the method of Schaaff and colleagues [10], with the neutral thioglucose ligand substituted for glutathione. The extinction coefficient for thioglucose-passivated nanocrystals at 360 nm is 8(±)×104 M-1 cm-1 (Fig. S1).

### Synthesis and purification of oligonucleotides

The DNA sequences used in this study are reported in Table 1 in Supplementary Materials S1. All oligonucleotides were prepared on an automated ABI 394 DNA synthesizer (Applied Biosystems) and retained a 5′-dimethoxytrityl (DMT) group when cleaved from the resin. Thiols were incorporated into the oligonucleotides using the Glen Research C3-thiol-modifier (part # 20-2933-41). The synthetic oligonucleotides were purified by a combination of ion-exchange and reverse-phase high-pressure liquid chromatography (HPLC).

**Table 1:** DNA thermal stability with and without covalently linked gold nanocrystals.

Number of Base Pairs	Unmodified (U) T_m_ (°C)	Double-Labeled (AB) T_m_ (°C)	Δ T_m_ (AB-U)	Single-Labeled (A) T_m_ (°C)	Single-Labeled (B) T_m_ (°C)	Δ T_m_ (A-U)	Δ T_m_ (AB-B)
10	45.0±0.2	46.4±0.1	+1.4	-	-	-	-
15	56.1±0.3	58.1±0.1	+2.0	-	-	-	-
20	65.0±0.5	67.7±0.2	+2.7	-	-	-	-
25	68.7±0.3	70.0±1.0	+1.3	69.4	69.6	+0.7	+0.4
30	73.5±0.1	77.0±1.0	+3.5	75.1	75.5	+1.6	+1.5
35	79.9±0.6	81.7±0.5	+1.8	80.9	80.9	+1.0	+0.8

Melting temperatures for unmodified, single-labeled and double-labeled DNA duplexes. The increase in melting temperature upon addition of the A nanocrystal is the same whether or not the B nanocrystal is present, indicating a zero coupling energy between the two nanocrystals. The samples were approximately 10 µM concentration in 1 M NaCl, 0.05 mM EDTA, and 10 mM sodium phosphate, pH 7.0. The reported error is the difference between three independent measurements.

### Coupling of gold nanocrystals to oligonucleotides

Coupling of gold nanocrystals to single-stranded DNA (ssDNA) was accomplished by mixing 60 nmols of thiol-modified DNA oligonucleotide with a five-fold molar excess of gold nanocrystals in 100 µL of 100 mM Tris-HCl, pH 9.0, for two hours. The nanocrystal-ssDNA conjugates were purified away from uncoupled gold nanocrystals, and from gold nanocrystals coupled to multiple ssDNA strands, by ion-exchange HPLC (Dionex, DNAPac PA-100) and stored at −20°C (Fig. S2). For concentration measurements, aliquots were diluted 4-fold into water and quantified by absorbance at 260 nm and 360 nm using a NanoDrop ND-1000 (NanoDrop Technologies). Concentrations measured after X-ray exposure of the samples agreed with the initial measurements to within 15%.

### Thermal Melts, Circular Dichroism and Mass Spectrometry

Thermal melts were measured for solutions of 5–10 µM dsDNA in a buffer consisting of 10 mM sodium phosphate, pH 7.0, 0.05 mM EDTA and 1 M NaCl. Circular dichroism spectra were recorded (190–350 nm in 1 nm increments) on solutions of 1–10 µM dsDNA at 20°C in a buffer consisting of 70 mM Tris-Cl, pH 8.0, and 100 mM NaCl. Matrix assisted laser desorption/ionization time of flight spectra were obtained for the gold nanocrystals by co-spotting 2 µL of the stock solution (20 mg/mL in water) with 2 µL of α-cyano-4-hydroxycinnamic acid matrix (CHCA, Sigma) on a stainless steel target.

### X-ray scattering

Small-angle X-ray scattering experiments were performed at the BESSRC-CAT beamline 12-ID of the Advanced Photon Source (APS) and at beamline 4-2 of the Stanford Synchrotron Radiation Lab (SSRL). At APS, the beam was tuned to 12 keV and the sample-detector distance was 1 meter. A silver behenate [11] standard was used to locate the beam center and calibrate the scattering angle values. Data were collected on a Gold CCD camera in ten one-second exposures per sample. Data reduction was performed with the Goldcontrol software package [12]. At SSRL the beam was tuned to 9 keV and the sample-detector distance was 1.5 meters. Silver behenate and cholesterol myristate [13] standards were used to locate the beam center and calibrate the scattering angle values. Two different detectors, a linear position sensitive proportional counter (LPSPC) [14] and a Mar CCD165 (MAR), were used. On the LPSPC detector, data were collected in ten one-minute exposures and reduced with the OTOKO and SAPOKO [15] software packages. On the MAR detector, data were collected in twenty fifteen-second exposures and reduced with the Blue Ice software package and with auxiliary scripts at the beamline.

Immediately before each measurement, a 20 µL sample containing 100 µM dsDNA was prepared by thawing the appropriate stock solution to room temperature and diluting it into a buffer of 100 mM NaCl, 70 mM Tris-HCl, pH 8.0, and 10 mM ascorbic acid final concentrations (unless otherwise indicated). The measurements were carried out in a 2 mm path length cell with 25 µm mica windows [16]. Scattering from a buffer standard was recorded before and after each sample. Scattering curves for dsDNA samples at 50 µM matched those at 400 µM indicating that interparticle interference, aggregation, and duplex dissociation effects were negligible. With anticipated upgrades to detectors and beamlines, we expect that molecular concentrations lower than 50 µM should provide sufficient signal-to-noise for these measurements in the near future.

### Data Analysis

Using MATLAB scripts, the raw scattering data were scaled and summed to obtain nanocrystal interference patterns. These patterns were transformed into inter-nanocrystal center-of-mass distance distributions using a non-negative least squares algorithm. See Supplemental Materials S2, which is published as supporting information on the *PLoS ONE* web site, www.plosone.org, for the commented MATLAB scripts.

Data scaling was carried out in the following sequence. First, we buffer-subtracted the scattering profile measured for the gold probes alone (auto-scattering intensity profile, *I_Au_*) so that its signal would tend to zero at the highest scattering angle measured. We then scaled it so that the Guinier extrapolated zero-angle intensity of the gold profile would evaluate to one. A single buffer profile (*I_Buf_*) was scaled by the same constant to serve as a buffer intensity reference. Second, the two single-labeled DNA scattering intensity profiles (*I_A_* and *I_B_*) and the double-labeled DNA scattering intensity profile (*I_AB_*) were buffer-subtracted and scaled so as to superimpose onto the tail of the gold auto-scattering profile at *S*≥0.04 Å^−1^. Finally, the unlabeled DNA profile (*I_DNA_*) was buffer-subtracted so that its signal would tend to zero at high angle, and then scaled so that the Guinier extrapolated intensity at zero angle would evaluate to (0.0334 * # base pairs)^2^. The constant 0.0334 is the ratio of the molecular weight of a base pair (650 Daltons) to the molecular weight of the nanocrystal (∼19455 Daltons), specifically 0.0334 = 650/19455. Variances for the profiles were calculated by adding and scaling the corresponding sample and buffer variances.

Starting with the crudely scaled data, we determined final scaling coefficients by a more elaborate procedure based on ensuring that the sinusoidal oscillations in the nanocrystal interference pattern average to zero, and that the radial Patterson derived from the interference pattern does not include negative values (which are physically impossible). First, we obtained inter-atomic radial Pattersons [5] (denoted *U_Au_*, *U_Buf_*, *U_A_*, *U_B_*, *U_AB_* and *U_DNA_*) by decomposing each scattering intensity profile into a linear combination of basis profiles of functional form *sin*(2*πSD*)/(2*πSD*):
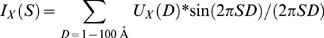
The basis profiles were calculated with distance (*D*) incremented in discrete 5 Å intervals from 1 to 200 Å, and the decomposition was performed using the non-negative least-squares optimizer in MATLAB. The scaled profiles and the corresponding radial Pattersons were then summed to generate a gold-gold scattering interference profile (*I_Δ_*) and a gold-gold interference Patterson (*U_Δ_*) as:
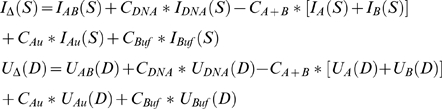
We defined the optimal values for the four scaling coefficients (*C_DNA_*, *C_A+B_*, *C_Au_* and *C_Buf_*) as those that minimized the function *T*:
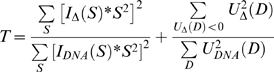
Minimization of *T* insures that the sinusoidal oscillations in I_Δ_(S)*S average to zero, and that *U_Δ_* does not include negative values. Variances for the interference profiles were obtained by adding the scaled variances of the constituent profiles.

In order to calculate center-of-mass distributions, it is necessary to calculate the form factor for a pair of gold nanocrystals. To this end, the gold nanocrystal radius distribution, *P_Au_(R)*, was obtained by decomposing the gold auto-scattering profile (*I_Au_*) into a linear combination of basis profiles, *I_R_(S)*, corresponding to hard-sphere scatterers of different radii *R*:
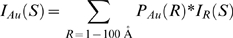
The scattering intensity and form factor (*I_R_* and *f_R_*) for a gold sphere of radius *R* are computed as [17]:
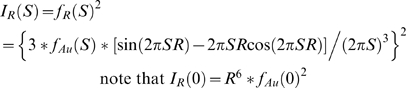
Here *f_Au_(S)* is the gold form factor approximated as a sum of Gaussian functions (International Tables of Crystallography, volume C). The zero-angle amplitude of these hard-sphere basis functions grows as *R^6^*. Decomposition of the gold auto-scattering signal into a linear sum over them was not numerically stable. However, if we used hard-sphere basis functions that were normalized to a common value at zero angle:
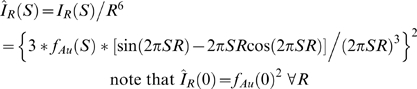
then the decomposition proceeded smoothly. The basis profiles were calculated with the nanocrystal radius incremented in discrete 1 Å intervals from 1 to 100 Å, and the decomposition was performed using the non-negative least-squares optimizer in MATLAB. As a consequence of the basis-function normalization, the linear coefficient for each normalized hard-sphere basis function is weighted up by a factor of R^6^:
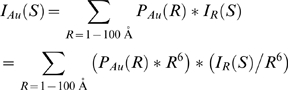
Accordingly, we divided the linear coefficients from the normalized basis functions by *R^6^* in order to generate *P_Au_(R)*, the unweighted gold nanocrystal radius distribution. Finally, the virtual intensity profile for scattering interference between a pair of nanocrystals sampled from the nanocrystal distribution, and situated with a center-to-center separation of 0 Å, was calculated as the probability-weighted average over the hard-sphere scatterer form factors:
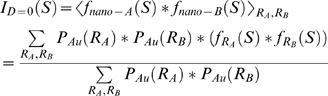
Note that if the gold nanocrystals were perfectly homogeneous, this average would be identical to the experimentally measured *I_Au_*.

Following the Debye formula [5], the positive inter-crystal distance distributions, *P_Δ_(D)*, were obtained by decomposing *I_Δ_(S)* into a linear combination of scattering interference profiles, *I_D_(S)*, corresponding to pairs of nanocrystals with varying center-of-mass separations (*D*):
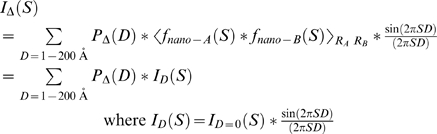
The basis profiles were calculated in discrete values of D between 1 and 200 Å and the decompositions were performed with the non-negative least squares optimizer in MATLAB. Alternatively, we used the MemSys5 quantified maximum entropy algorithm [18], modified so that a cross-validation statistic determined the stopping value of the regularization coefficient [19]–[20]. Thus a random selection of 10% of the observed scattering profile, in five contiguous blocks of 2%, was removed from the data set and designated as a control set. The remaining 90% of the data was used to construct a probability distribution. The regularization coefficient was annealed downward from a large starting point, and the value that minimized the χ^2^ statistic of the model distribution over the control data set was determined. This process was repeated ten times with different random choices of the control set, and the ten optimum values of the regularization coefficient were averaged geometrically. A final probability distribution was constructed using all of the data and the averaged stopping value. Observable properties of the distribution, such as peak centers and variances, were determined by fits of the reconstructed distributions to Gaussian curves.

## Results

**Figure 1:**
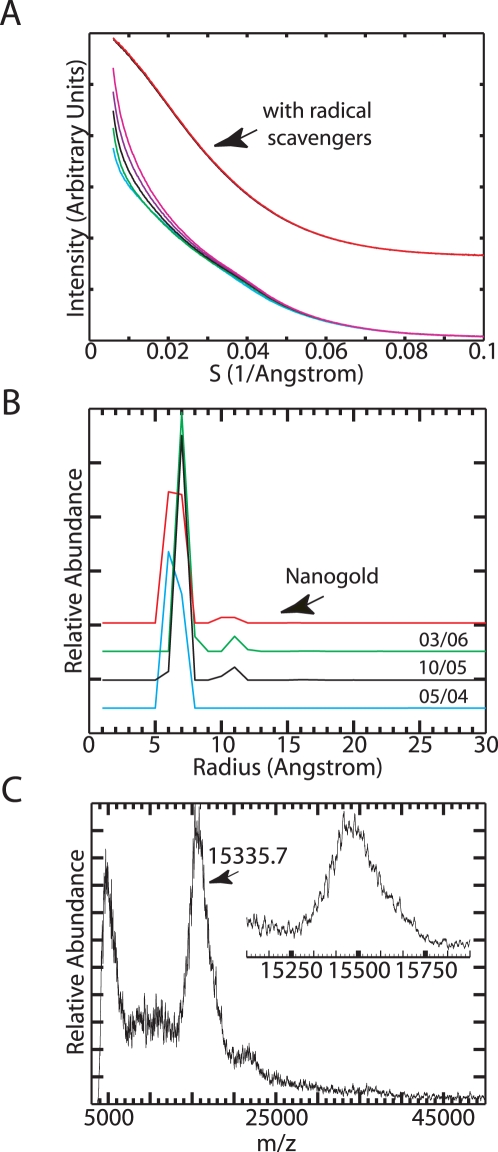
Characterization of thioglucose passivated gold nanocrystals. (A) Five superimposed gold autoscattering frames measured in the presence of radical scavengers (top) are vertically offset from five autoscattering frames collected in the absence of radical scavengers (bottom). The frames were collected at one second intervals. Tris (70 mM) and ascorbic acid (10 mM) were used to suppress radiation damage. (B) Gold nanocrystal radius distributions measured for commercially available Nanogold (Red) and three independent thioglucose nanocrystal preparations (labeled with the month/year in which they were prepared). (C) Negative ion matrix assisted laser desorption/ionization mass spectrum of the thioglucose nanocrystal. The inset shows the m/z range centered at 15,500.

As a potential heavy atom probe for a scattering interference ruler, we synthesized thioglucose passivated gold nanocrystals by the Brust method [10], [21]. In the presence of the radical scavenging agents Tris-HCl and ascorbic acid (Fig. 1A), the nanocrystal auto-scattering profile was stable for more than 200 seconds on the high-flux BESSRC-CAT 12ID-C beamline. We determined the size distribution of our nanocrystal preparation by decomposing the auto-scattering profile into a linear combination of basis profiles corresponding to hard sphere scatterers of varying diameter. The data indicate that the nanocrystals consist predominantly of 14 Å diameter spheres (Fig. 1B). The size distribution closely resembles the distribution we measure for commercially available Nanogold (Nanoprobes), and corresponds to a particle containing 75 gold atoms [22]. We also determined the molecular weight of the gold core of the particles using matrix assisted laser desorption/ionization mass spectrometry [23]. The dominant peak at m/z = 15,335 corresponds to 78 gold atoms and a sphere of 14 Å diameter (Fig. 1C). Thus the diameter of the nanocrystal probes is similar to the dimensions of an organic fluorophore.

**Figure 2:**
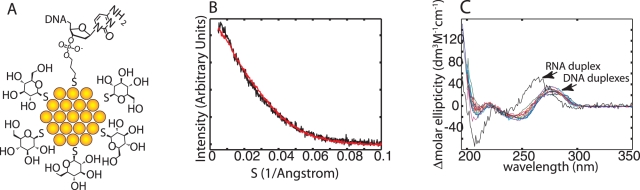
Nanocrystal attachment to DNA. (A) Schematic of thioglucose passivated gold nanocrystals coupled to a DNA oligonucleotide bearing a 3′ sulfhydryl group. The sulfhydryl group forms a bond directly to the nanocrystal core. (B) The scattering profile of a double-labeled 30 base-pair DNA duplex that was digested with 5 Units of DNase I (Black) is superimposed on the auto-scattering profile of the nanocrystal starting material (Red). The coincidence of the two profiles shows that the nanocrystal size distribution is not altered by attachment to DNA. (C) Circular dichroism (CD) spectra of unlabeled (Magenta, Red and Green) and double-labeled (Cyan, Gray, Gold and Blue) DNA duplexes are shown. Twelve spectra corresponding to 10, 15, 20, 25, 30, and 35 base-pair samples are included. The CD spectra of the DNA duplexes are not changed by covalent attachment of the nanocrystals, indicating that the B-form helix is not altered. The CD spectrum of a 500 base-pair A-form RNA duplex (Black) is also shown to illustrate the sensitivity of CD spectra to helix geometry.

To investigate how the nanocrystals might perturb the structure of a macromolecule, we coupled them to the 3′-ends of DNA strands via a sulfhydryl-gold bond (Fig. 2A, the coupling process does not measurably change the nanocrystal size distribution). We checked for possible nanocrystal-induced changes in DNA structure by comparing the melting temperatures (ΔT_m_'s) and circular dichroism spectra (CD) of double-labeled and unlabeled DNA duplexes (Table 1, Fig. 2C). For six different DNA lengths, the largest ΔT_m_ is 3.5°C. Double-labeled samples always exhibit higher melting temperatures than unlabeled samples. The CD spectra of labeled and unlabeled duplexes (Fig. 2C) are indistinguishable from one another. Collectively, the data indicate that the nanocrystals minimally perturb DNA structure.

**Figure 3:**
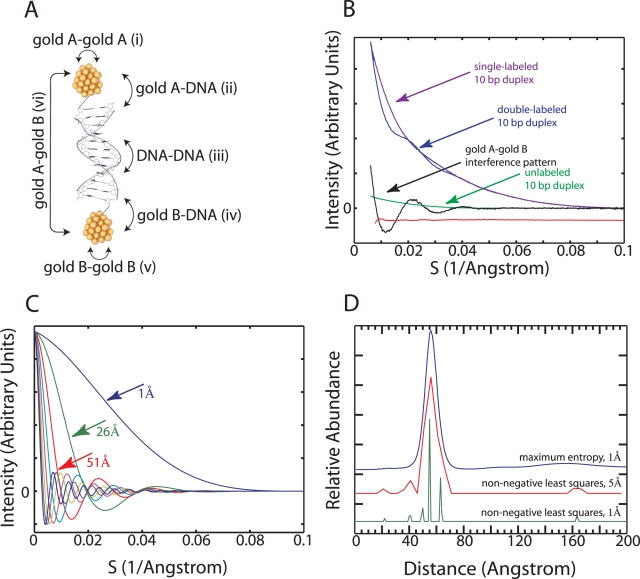
Transformation of scattering interference profiles into distance distributions. (A) Model coordinates of a 12 base-pair DNA duplex bearing a gold nanocrystal at each end. Thioglucose ligands are not shown. Various types of scattering interference between gold nanocrystals and DNA are illustrated with labeled arrows. The probe-probe interference pattern (vi) is obtained by subtracting the scattering profiles for the single-labeled A sample (i+ii+iii), and B sample (iii+iv+v) from the sum of the double-labeled sample (i+ii+iii+iv+v+vi) and the unlabeled sample (iii). (B) The scattering profiles for the 10 base-pair double-labeled (Blue), single-labeled (Purple, Magenta; indistinguishable), and unlabeled (Green) DNA duplexes. The probe-probe scattering interference pattern (Black) is obtained by adding the double-labeled and unlabeled profiles and subtracting off the single-labeled profiles. The residual difference between this interference pattern and the transform of the probability distribution in panel D is plotted in Red, and offset downward. See Fig. S6 for a log-log plot of the scattering profiles. (C) Scattering interference basis profiles corresponding to pairs of nanocrystals with center-to-center separation distances between 1 and 200 Å. The profiles are plotted at 25 Å increments for clarity. (D) Distance distributions obtained by decomposing the scattering interference pattern in panel B into a linear combination of the basis profiles shown in panel C. Three different transformation methods are illustrated. They are offset vertically from one another. The bottom profile (Green, 56.3 Å±3.1 Å) was obtained using 1 Å resolution basis functions and a non-negative least-squares optimizer. The middle profile (Red, 56.8 Å±3.2 Å) was obtained using 5 Å resolution basis functions and a non-negative least-squares optimizer. The top profile (Blue, 56.8 Å±3.0 Å) was obtained using 1Å resolution basis functions and a non-negative least-squares optimizer with a maximum entropy regularization term. The least-squares transformation of a scattering interference profile into a distance distribution is unique for coarsely sampled distance basis functions. The highest basis set resolution that retains this property is 1/(2*S_max_), where S_max_ is the scattering angle at which the signal-to-noise ratio reaches ∼2. For the data presented here, S_max_≈0.08 Å^−1^, giving a natural basis function resolution of 6 Å. Transforms at basis set resolutions higher than this value generally require some form of regularization to break the degeneracy between multiple possible solutions.

To determine the scattering interference pattern between two gold nanocrystals attached to a DNA duplex, five different scattering profiles were collected [2]–[4]. These profiles derived from samples of gold nanocrystals alone, DNA alone, two single-labeled DNA duplexes and a double-labeled DNA duplex (Fig. 3A). The profiles were scaled relative to each other and then summed to generate a probe interference pattern. Interference patterns typically exhibited low angle signal-to-noise ratios of ∼100 for a one second exposure of a 100 µM sample on the BESSRC-CAT 12ID-C beamline (Fig. 3B).

The distance distribution between the centers-of-mass of the nanocrystals was obtained by decomposing their interference pattern into a linear combination of basis patterns corresponding to interference between two gold nanocrystals at varying center-to-center separation distances (Fig. 3C). We performed this decomposition by conventional non-negative least squares, for example using the lsqnonneg function of MATLAB (Fig. 3D). The distance distributions obtained this way sometimes exhibited numerous sharp peaks instead of a single smooth peak. Alternatively, we obtained distributions by non-negative least squares fitting with an additional maximum entropy regularization term. Cross-validation was used to determine the regularization coefficient. The distributions presented in the figures were obtained with the maximum entropy procedure, unless stated otherwise.

**Figure 4:**
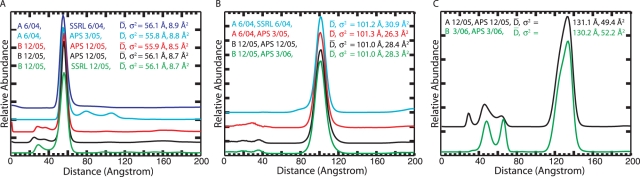
Repeat measurement of distance distributions using independently prepared samples and two different synchrotron X-ray sources. Data for the 10 [A], 25 [B], and 35 [C] base-pair duplexes are shown. Independent samples are labeled A and B followed by the month/year in which they were prepared. The plot key also indicates the synchrotron source (SSRL – Stanford Synchrotron Radiation Laboratory, APS – Advanced Photon Source) followed by the month/year in which the data were collected. The mean and variance of a Gaussian fit to each distribution is reported. The dominant feature of each distribution is extremely reproducible. The smaller variable distribution features appear to correlate with sample preparation and freezer storage time (see sample A in panel A).

We assessed the reproducibility of the scattering interference ruler by repeating distance distribution measurements on independently synthesized samples at two different synchrotron sources (Fig. 4). The samples included end-labeled 10, 25 and 35 base-pair duplexes. We observed small (∼5%) and variable features in the baseline of each distribution that were idiosyncratic to the sample preparation (they likely arise from impurities). However, the dominant probability features were constant. For all three duplexes, the coefficient of variation is less than 1% for the mean of the dominant feature, and less than 7% for the variance. Degradation of the data quality by truncation at low angle and by addition of white noise did not significantly perturb the mean and variance (Fig. S3). Thus, the scattering interference ruler is extremely robust.

**Figure 5:**
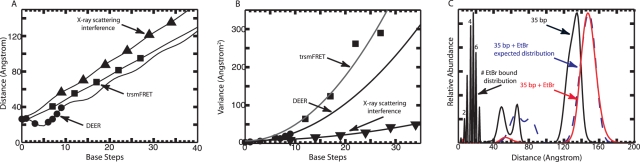
Quantitative accuracy of the first and second moments of the distance distributions. (A) Mean probe-probe separation distances within labeled duplexes is plotted with respect to the number of intervening DNA base-pair steps. Distances measured by X-ray scattering interference, time-resolved single-molecule fluorescence resonance energy transfer (trsmFRET) and double electron-electron spin resonance (DEER) are shown. A three-variable model accounting for rotation of the nanocrystal probes around the helix axis is fit to each data set, giving a fitted helical rise value (Fig. S4). An average rise of 3.32±0.19 Å is observed in naked DNA crystal structures. The scattering interference data fit to a helical rise of 3.29±0.07Å, and a 9Å radial displacement of the nanocrystals off of the helix axis (R^2^ = 0.999). The trsmFRET data fit to a helical rise of 2.74Å, and 12Å/−2Å radial displacements of the fluorophores off of the helix axis (R^2^ = 0.999). The DEER data fit to a helical rise of 3.01Å, and a 13Å radial displacement of the spin labels off of the helix axis (R^2^ = 0.983). The data sets are taken from [24], [33]–[34]. All distances were corrected for the apparent shortening caused by DNA bending (Table 2 in Supplementary Materials S1). (B) The variance in probe-probe separation distance within labeled duplexes is plotted with respect to the number of intervening DNA base-pair steps. The variance grows more rapidly with DNA length in the DEER and the trsmFRET data than in the X-ray scattering data. (C) Comparison of the expected and the observed broadening of a 35 base-pair duplex distance distribution by sub-saturating ethidium bromide. The initial distribution (Black) exhibits a mean of 131.9 Å and a variance of 51 Å^2^. The distribution in the presence of ethidium (Red) exhibits a mean of 147.7 Å and a variance of 71 Å^2^. The 15.8 Å shift in the mean corresponds to an average of 4.65 ethidium intercalation events per duplex. Individual duplexes can bind between zero and eight ethidium molecules at pyrimidine-purine base steps. The relative abundance of duplexes with different numbers of bound ethidium molecules is plotted at the left (Black, labeled #EtBr bound distribution). The position of each peak on the horizontal axis corresponds to the increase in duplex length caused by ethidium intercalation. The expected distribution for a 35 base-pair duplex in the presence of ethidium, calculated as the convolution of the distribution in the absence of ethidium with the number of ethidium bound distribution, is shown as a dashed blue line. The expected increase in variance of 22.5 Å^2^ matches closely to the observed increase of 20 Å^2^.

Two experiments establish the quantitative accuracy of the scattering interference ruler. First, we used it to determine the helical rise of DNA in solution (reported in [24]). Six end-to-end distance distributions for duplexes between 10 and 35 base-pairs were measured and fit to a three-variable model of the DNA helix (Fig. 5A and Fig. S4). The data indicate a helical rise value of 3.29±0.07 Å, in close agreement with the crystallographic average value of 3.32±0.19 Å [25]. By comparison, identical experiments with two state-of-the-art spectroscopic rulers (a single-molecule fluorescence resonance energy-transfer ruler and a double electron-electron spin resonance ruler) give rise values of 2.74 Å and 3.01 Å respectively. The distance variation measured by the scattering interference ruler is much smaller than the apparent variation measured by spectroscopic rulers (Fig. 5B, Fig. S5).

Second, we compared the expected and observed broadening in the end-to-end distance distribution of a 35 base-pair duplex caused by ethidium bromide. Ethidium binding increased the mean length of the 35mer by 15.8 Å and increased its length variance by 20 Å^2^ (Fig. 5C). In crystal structures and nuclear magnetic resonance structures of polynucleotide-ethidium complexes, each ethidium intercalation event lengthens the DNA by 3.4 Å [26]–[27]. Thus, 15.8 Å corresponds to an average of 4.65 ethidium molecules bound per duplex. Under the low saturation conditions of our experiment, ethidium intercalates non-cooperatively at pyrimidine-purine base steps [28]–[30]. Each 35mer duplex contains eight pyrimidine-purine base steps, and can thus bind between zero and eight ethidium molecules (with an average of 4.65). The relative abundance of each type of DNA/ethidium complex can be calculated using the binomial distribution (Supplemental Methods in Supplementary Materials S1). By this calculation, the variability in the number of ethidium molecules bound per duplex should increase the variance of the 35mer end-to-end distance distribution by 22.5 Å^2^. This expected broadening corresponds closely to the 20 Å^2^ increase in variance that we observe (Fig. 5C).

**Figure 6:**
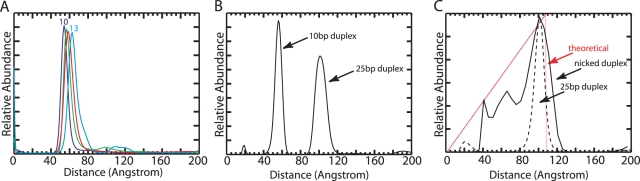
Resolution and complex distributions. (A) End-to-end distance distributions for independent measurements of 10 (Blue), 11 (Green), 12 (Red), and 13 (Cyan) base-pair DNA duplexes. Scattering profiles were acquired in 10 mM Na-MOPS pH 7.5 and 100 mM NaCl, in the absence of radical scavengers. (B) The distance distribution for a 1∶1 mixture of 10 and 25 base-pair duplexes. The distribution shows two peaks in relative proportions of 45% (left peak, centered at 56.7 Å) and 55% (right peak, centered at 100.8 Å). (C) Distance distribution for a nicked duplex sample consisting of two 12 base-pair duplexes linked by three unpaired nucleotides (Black). The distance distribution for a 25 base-pair duplex is also shown for comparison (dashed line). The solid red line corresponds to the expected distribution for weakly-interacting linked segments: dAbundance/dDistance = 0.5 * Distance/(segment length)^2^.

Finally, we investigated the length resolution of the scattering interference ruler as well as its ability to recover complex distributions. First, we measured the end-to-end distance distributions of four DNA duplexes with 10, 11, 12 and 13 base pairs. The distributions overlap, but consistently tend to longer distances (Fig. 6A). Thus, the ruler is capable of resolving single base-pair increments in DNA length, corresponding to approximately one-fifth of the diameter of the gold nanocrystal. Next, we created a sample of heterogeneous length by mixing equal quantities of labeled 10 and 25 base-pair duplexes. The corresponding distribution shows two peaks separated by 45 Å, in relative proportions of 45% and 55% (Fig. 6B). Third, we measured the end-to-end distribution of a floppy macromolecule, a nicked 27 base-pair duplex consisting of two 12 base-pair duplexes linked by three unpaired T nucleotides [31]. At 1 M NaCl, the nicked duplex exhibits a broad, unimodal distribution (Fig. 6C), with a mean distance of 93 Å. The roughly triangular shape of the distribution is expected for a pair of weakly-interacting freely-jointed segments. This type of measurement should provide a powerful test of theoretical models for the conformational fluctuations of macromolecules. Taken together, the results establish that the scattering interference ruler is applicable to heterogeneous samples that exhibit a wide range of different probe-probe separation distances.

## Discussion

The scattering interference ruler complements existing molecular ruler techniques in several ways. As illustrated here, it provides calibrated distances and the ability to record complex distributions. Another unique property is that it yields an instantaneous snapshot of a molecular ensemble, because movements of nuclei are slow with respect to the electronic transitions that produce scattering interference. Thus all macromolecule and probe dynamics are in slow exchange, and contribute to the observed distance distribution. By comparison, spectroscopic rulers operate on an intrinsic timescale (for example the excited state lifetime of a fluorophore). Distinct distances associated with movements that are in fast exchange on the intrinsic timescale collapse into a single distance. Paradoxically, however, the observed scattering interference distributions are narrower than the spectroscopic ruler distributions. Inexplicably broad distributions also have been observed in smFRET measurements on polyproline helices [32].

The range of a scattering interference ruler should extend beyond the 40–170 Å distances measured here. The short end of the range is limited by the diameter of the nanocrystal probe, although distance differences much smaller than the probe diameter can be resolved. In principle, nothing limits the long end of the range. In practice, however, longer distances are associated with broader distributions, which cause a more rapid decay of the scattering interference signal with scattering angle. Longer distances also produce higher frequency oscillations, so that the interference pattern should complete many cycles before disappearing into noise. The problem is that for sufficiently long distances, all of the measurable oscillations occur below the low angle cut-off of a typical small-angle X-ray scattering instrument. The solution to this problem is to decrease the low angle cut-off. This can be accomplished either by changing the optical geometry of existing X-ray beamline instruments, or by using longer wavelength incident radiation. Increasing the size of the nanocrystal probes would also facilitate longer distance measurements by improving the signal-to-noise of the scattering interference data in proportion to the nanocrystal volume.

An important application of the X-ray ruler will be measurement of distance distributions within proteins. Analysis of proteins should be simpler than analysis of nucleic acids, because the electron density of proteins can be contrast matched with aqueous sucrose solutions. Under these conditions, single-labeled and unlabeled profiles become unnecessary, and data scaling is greatly simplified (this should hold for any material with electron-dense centers if the bulk material can be contrast matched to the solvent). Exciting future possibilities include time-resolved measurement of distance distributions by X-ray scattering with continuous rapid mixing and combined electron-microscopy/solution X-ray experiments using the gold nanocrystal probes to label structural domains.

## Supporting Information

Supplementary Materials S1 Supporting Information Text
(0.15 MB DOC)

Supplementary Materials S2 MATLAB Data Analysis Scripts
(2.42 MB ZIP)

Figure S1 Thioglucose-passivated nanocrystal absorbance spectra.
(1.26 MB EPS)

Figure S2 Purification of nanocrystal labeled DNA.
(1.91 MB EPS)

Figure S3 Effect of reduced signal-to-noise on distance distributions.
(1.27 MB EPS)

Figure S4 Geometric model of the double helix used to fit distance data.
(2.00 MB EPS)

Figure S5 Probability distance distributions.
(1.10 MB EPS)

Figure S6 Log-log plot of scattering interference profiles.
(1.49 MB EPS)
