# Supplementary material for: A Molecular Ruler for Measuring Quantitative Distance Distributions
Source: PLoS One. 2008 Oct 17;3(10):e3229. doi: 10.1371/journal.pone.0003229 (PMC2566812; doi:10.1371/journal.pone.0003229)
Supplement: Supplementary Materials S1 — Supporting Information Text (0.15 MB DOC) [file pone.0003229.s001.doc]

Supplementary materials section for

“A molecular ruler for measuring quantitative distance distributions”

Rebecca S. Mathew-Fenn^a^, Rhiju Das^b,c^, Joshua A. Silverman^d^, Peter A. Walker

and Pehr A.B. Harbury^*^

Department of Biochemistry, Biophysics Program^a^, and Department of Physics^b^, Stanford University, Stanford, CA 94305

^c^Current address: Department of Biochemistry, University of Washington, Seattle, WA 98195

^d^Current address: Avidia Inc., 2450 Bayshore Parkway Mountain View, CA 94043.

^*^To whom correspondence should be addressed.

Email: harbury@cmgm.stanford.edu

This pdf file includes:

Materials and Methods

Supplementary Table 1-2

Supplementary Figures 1-6

References

# Supplementary Methods

## Synthesis and purification of nanocrystals:

Synthesis of water‑soluble gold nanocrystals followed the method of Schaaff and colleagues [1], with the neutral thioglucose ligand substituted for glutathione. Tetrachloroauric(III) acid (1.6 mmol) was dissolved in 36 mL of a 6:1 mixture of methanol:acetic acid, resulting in a deep orange colored solution. 1‑ß‑D‑Thioglucose (4.8 mmol) was fully dissolved in 36 mL of a 6:1 mixture of methanol:acetic acid and then added to the tetrachloroauric acid solution, producing a cloudy orange mixture. Using an addition funnel, an excess of aqueous sodium borohydride (1.2 g in 30 mL water) was dripped into the stirring suspension over ten minutes, causing a gradual color change of the solution from cloudy orange to black. After further stirring for thirty minutes at room temperature, the solution was concentrated ten‑fold by rotary evaporation, filtered, and desalted over Sephadex G15 resin (Amersham Biosciences). The desalted nanocrystals were then passed over a gel‑filtration column (Amersham Biosciences Superdex‑75) equilibrated in 150 mM ammonium acetate, pH 5.5, to isolate a gold nanocrystal stock solution with good size homogeneity. The gold nanocrystals were desalted a final time through G15 resin (long term storage of the nanocrystals in high salt solutions induced aggregation). The yield of brown thioglucose‑passivated nanocrystals was typically about 10 µmols, determined by absorbance using an extinction coefficient at 360 nm of 8(±1)X10^4^ M^‑1^cm^‑1^.

## Synthesis and purification of oligonucleotides:

The DNA sequences used in this study are reported in Table SI. All oligonucleotides were prepared on an automated ABI 394 DNA synthesizer (Applied Biosystems) and retained a 5'‑dimethoxytrityl (DMT) group when cleaved from the resin. Thiols were incorporated into the oligonucleotides using the Glen Research C3‑thiol‑modifier (part # 20-2933-41). Following deprotection in concentrated ammonium hydroxide, the oligonucleotides were purified by ion‑exchange HPLC (Dionex DNAPac PA100) with a linear gradient from 20 mM to 1.5 M sodium chloride in 25 mM Tris‑HCl, pH 8.0. A reverse‑phase HPLC purification step (Zorbax Eclipse XDB‑C18) with a linear gradient from 0 to 90% acetonitrile in 0.1 M triethylammonium acetate, pH 5.5, was then performed to remove truncation products. Full‑length product fractions were concentrated ten‑fold by centrifugal evaporation. DMT groups at the 5'‑terminus were removed by addition of trifluoroacetic acid (0.11 volume equivalents or until pH 3 was obtained) for five minutes at room temperature, followed by dropwise addition of triethylamine (0.22 volume equivalents or until pH 7 was obtained). The oligonucleotides were then immediately precipitated by addition of 10 mM magnesium chloride and five volumes of ethanol. Just prior to gold coupling reactions, thiol‑containing oligonucleotides were further deprotected by treatment of the resuspended DNA with 100 mM dithiothreitol (DTT) for 30 minutes at 70 °C. Excess DTT was removed by transferring the solution to a fresh tube, followed by a second ethanol precipitation step, as above.

## Coupling of gold nanocrystals to oligonucleotides:

Coupling of gold nanocrystals to single‑stranded DNA (ssDNA) was accomplished by mixing 60 nmols of thiol‑modified DNA oligonucleotide with a five‑fold molar excess of gold nanocrystals in 100 µL of 100 mM Tris‑HCl, pH 9.0, for two hours. The gold‑ssDNA conjugates were purified away from uncoupled gold nanocrystals, and from gold nanocrystals coupled to multiple ssDNA strands, by ion‑exchange HPLC (Dionex, DNAPac PA‑100) using a linear gradient over 80 minutes from 10 mM to 2 M ammonium acetate (pH 5.5) in 10% acetonitrile. Gold‑ssDNA conjugates eluted from the column before uncoupled ssDNA. The purified conjugates were then incubated with an appropriate complementary ssDNA (either gold labeled or unlabeled, depending on the sample) for 30 minutes at room temperature, and the desired double‑stranded DNA (dsDNA) was purified away from excess ssDNA by ion‑exchange HPLC as described above. Product fractions were pooled in a Centriplus YM‑3 concentrator (Millipore), diluted to 10 mL final volume with deionized water, and spun at 4 °C in a swinging bucket rotor at 3000 x g for five hours. Twice again, the concentrate was diluted with 10 mL deionized water and spun at 4 °C for five hours. On the third iteration, the concentrator was spun dry (approximately eight hours). The membrane was rinsed with 50 µL of water, and the device was inverted and spun for 4 minutes at 2500 x g to collect the dsDNA. Aliquots of 2 µL volume were removed for concentration determination, and the remaining material was immediately transferred to low‑binding tubes (Sorenson, part #16070), flash frozen in liquid nitrogen and stored at -20 °C. For concentration measurements, aliquots were diluted 4‑fold into water and quantified by absorbance at 260 nm and 360 nm using a NanoDrop ND‑1000 (NanoDrop Technologies). An extinction coefficient for the gold nanocrystals of 8(±1)X10^4^ M^‑1^cm^‑1^ at 360 nm and 21.5(±2)X10^4^ M^‑1^cm^‑1^ at 260 nm was determined by deconvolving the absorption spectra of purified gold‑dsDNA preparations. Concentrations measured after X-ray exposure of the samples agreed with the initial measurements to within 15%.

## Distance distributions from time‑resolved smFRET data:

Laurence *et al.* [2] report the first and second moment (mean $\hat{E}$ and standard deviation $\sigma_{E}$) for the efficiency distribution of fluorescence resonance energy transfer between donor and acceptor fluorophores coupled to DNA duplexes. The efficiencies are derived from time‑resolved single‑molecule FRET measurements that differentiate between states in slow exchange on the nanosecond timescale. The authors do not transform their data into distance distributions. We performed this transformation based on the assumption that the efficiency distributions, *dP(E)*, are Gaussian:

$$dP(E)=\sqrt{\frac{1}{2\pi\sigma_{E}^{2}}}e^{-\left(E-\hat{E}\right)^{2}/\left(2\sigma_{E}^{2}\right)}dE$$

We also assumed that the Förster expression for the relationship between transfer efficiency and inter‑probe distance, *L*, was applicable:

$$E\left(L\right)=\left[1+\left(L/R_{0}\right)^{6}\right]^{-1}$$

where *R_0_* is the Förster distance. Given these assumptions, distance probability distributions, *dP(L)*, were computed as:

$$\frac{dP(L)}{dL}=\int_{E(L+dL/2)}^{E\left(L-dL/2\right)} \frac{dP(E)}{dE}dE\approx\frac{dP(E(L))}{dE}\left[E\left(L-dL/2\right)-E(L+dL/2)\right]$$

The data from Laurence *et al.* included eight samples with a tetramethylrhodamine donor fluorophore and an Alexa 647 acceptor fluorophore:

| Base Steps | Donor | Acceptor | $\hat{E}$ | σ_E_ |
| --- | --- | --- | --- | --- |
| 5 | Internal | Internal | 0.909 | 0.051 |
| 7 | End | Internal | 0.958 | 0.0214 |
| 12 | End | Internal | 0.8 | 0.14 |
| 15 | Internal | Internal | 0.604 | 0.208 |
| 17 | End | Internal | 0.56 | 0.205 |
| 22 | End | Internal | 0.32 | 0.225 |
| 25 | Internal | Internal | 0.122 | 0.077 |
| 27 | End | Internal | 0.16 | 0.145 |

We used the value of the Förster distance (*R_0_*=69Å) that was measured by the authors [2].

## Expected broadening from ethidium bromide:

Sub‑saturating ethidium bromide binds non‑cooperatively at pyrimidine‑purine dinucleotide sites in DNA duplexes, lengthening the DNA by ~3.4 Å per intercalation event [3-7]. Eight pyrimidine‑purine sites exist in the 35 base‑pair duplex sequence used here (Table S1). Thus, a given duplex can bind between zero and eight ethidium molecules. The observed 15.8 Å increase in the mean end‑to‑end distance of the 35mer corresponds to an average of 4.65 binding events per duplex. Because the sites bind independently, the relative abundance of each type of complex (differing in ethidium to DNA stoichiometry) follows the binomial distribution:

$$P(n)=\frac{N!}{(N-n)!*n!}*(p)^{n}*(1-p)^{N-n}$$

Here, *n* denotes the duplex with *n* bound ethidium molecules and *P(n)* denotes its relative abundance. *N* denotes the number of binding sites, which is eight. The lower case *p* denotes the probability of ethidium binding at each single site, which is 4.65/8. Insertion of these parameters into the binomial distribution function gives the following results:

| # of Bound Ethidium  *n* | Relative Abundance  *P(n)* | DNA length increase  (Å) |
| --- | --- | --- |
| 0 | 0.0009 | 0 |
| 1 | 0.0105 | 3.4 |
| 2 | 0.0510 | 6.8 |
| 3 | 0.1416 | 10.2 |
| 4 | 0.2457 | 13.6 |
| 5 | 0.2728 | 17 |
| 6 | 0.1893 | 20.4 |
| 7 | 0.0751 | 23.8 |
| 8 | 0.0130 | 27.2 |

The broadening expected upon ethidium binding corresponds to the distance variance of this binomial distribution, which evaluates to (3.4 Å)^2^**N***p**(1-*p*) = 22.5 Å^2^.

# Supporting Figure Captions

Figure S1: *Thioglucose-passivated nanocrystal absorbance spectra.* Absorbance spectra for thioglucose-passivated nanocrystals (Black). The concentration of nanocrystals was determined by absorbance using an extinction coefficient at 360 nm (Black arrow) of 8 (±1)X10^4^ M^-1^cm^-1^.

Figure S2: *Purification of nanocrystal‑labeled DNA.* **[A]** Ion‑exchange HPLC chromatogram of a reaction mixture containing uncoupled gold nanocrystals (Au), a 25 base ssDNA‑gold conjugate (Au_25_S), and gold nanocrystals coupled to multiple ssDNA strands (Au_25_*). **[B]** Ion‑exchange HPLC chromatogram of a 25 base pair dsDNA‑gold conjugate (Au_25_D). The purification step after formation of duplexes eliminates excess ssDNA, and gold nanocrystals coupled to multiple dsDNA's (Au_25_*). **[C]** Ion‑exchange HPLC chromatogram of a purified 25 base‑pair dsDNA‑gold conjugate (Au_25_D) after desalting, concentration to 100 µM, and storage at -20 ºC for 14 days. **[D]** Ion‑exchange HPLC chromatogram of a 25 base‑pair dsDNA‑gold conjugate sample (Au_25_D) after exposure to X‑ray radiation during data collection.

Figure S3: *Effect of reduced signal-to-noise on distance distributions.* **[A]** Nanocrystal scattering interference data for the 10 (Red), 15 (Green), and 20 (Black) base-pair duplex samples were truncated at low values of *S*, and degraded by addition of white noise, so as to match the signal characteristics of the scattering interference data from the 35 base-pair duplex (Blue). The original interference patterns are reported elsewhere (see [8], Fig. 2A). **[B-D]** Probe center‑of‑mass distance probability distributions for the 10 [B], 15 [C] and 20 [D] base‑pair duplexes computed using the scattering interference data in [A]. The mean and variance of Gaussian fits to the full data sets are titled “Full Data:”. The mean and variance of Gaussian fits to the truncated data sets (blue solid lines) are titled “Truncated:”.

Figure S4: *Geometric model of the double helix used to fit distance data.* **[A]** Cartoon of a double helix labeled with two gold nanocrystals (black balls). The distance between the two probes *L*, as a function of the number of intervening base steps N, was fit as the Pythagorean sum of the axial and radial separation distances:

$$L(N)=\sqrt{axial^{2}+radial^{2}}=\sqrt{\left(axial_{0}+rN\right)^{2}+D_{_{1}}^{2}+D_{_{2}}^{2}-2D_{1}D_{2}\cos\left[\theta_{0}+\frac{2\pi}{10.4}N\right]}$$

The term *r* is the axial rise per base step. The terms *D_1_* and *D_2_* are the radial displacements of the two probes off of the helical axis. The term *axial_0_* is the axial distance between two probes separated by zero base steps. The term *θ_0_* is the azimuthal angle between two probes separated by zero base steps. **[B]** The labeled duplex viewed in projection along the helix axis with *θ* denoting the azimuthal angle between the probes. Using the law of cosines, the radial separation distance was calculated as: *radial^2^=D_1_^2^+D_2_^2^-2D_1_D_2_cos[θ]*. In cases with symmetrical probe geometry (specifically *D_1_=D_2_=D*), the radial separation distance simplifies to *radial* = 2*D*sin[*θ*/2]. The azimuthal angle as a function of base steps was calculated *θ*= *θ_0_* + 2*πN*/10.4. The constant in the denominator derives from the fact that 10.4 base pairs of DNA in solution make one full turn around the helix axis. Values for *θ_0_* were determined by inspection of the Dickerson dodecamer structure. For probe attachment to 3'‑phosphates (scattering interference data), *θ_0_* was set to 1.34*π*. For probe attachment to the exocyclic methyl groups of T bases (trsmFRET data), *θ_0_* was set to 1.58*π*. For probe attachment to 5'‑phosphates (DEER data), *θ_0_* was set to 0.95*π*. **[C]** Two gold nanocrystals separated by zero base steps. The nanocrystals are attached at the terminal 3'‑phosphate positions of an A:T base pair through three‑atom linkers. The axial separation between the nanocrystals corresponds to the parameter *axial_0_*. For the scattering interference data, *axial_0_* optimized to 23.9 Å. For the trsmFRET data, *axial_0_* optimized to 20.4 Å. For the DEER data, *axial_0_* optimized to 3.3 Å. Panel [A] was rendered using PovScript+ [9].

Figure S5: *Probability distance distributions.* Probability distance distribution curves for DNA duplexes measured by an electron spin resonance ruler (Blue), a time-resolved single-molecule FRET ruler (Red), and an X-ray scattering interference ruler (Black). The distributions are normalized to sum to unity and are labeled with the number of base-pair steps. The data sets are taken from [2, 8 and 10].

Figure S6: *Log-log plot of scattering interference profiles.* Log‑log plot of intensity as a function of scattering angle for the 10 base-pair double‑labeled (Blue), single-labeled (Purple, Magenta; indistinguishable), and unlabeled (Green) DNA duplexes. The absolute value of intensity versus scattering angle for the probe‑probe scattering interference profile (Black) is also shown.

Table S1:

| **Duplex Name** | | **Sequence** | **Molecular Weight** |
| --- | --- | --- | --- |
| **10** | **A** | **5**'**-GCATCTGGGC-3**'  **CGTAGACCCG** | **6057** |
|  | **B** |  |  |
| **11** | **A** | **5**'**-GGCATCTGGGC-3**'  **CCGTAGACCCG** | **6675** |
|  | **B** |  |  |
| **12** | **A** | **5**'**-CGCTACGGAAGG-3**'  **GCGATGCCTTCC** | **7292** |
|  | **B** |  |  |
| **13** | **A** | **5**'**-CGACTACGGAAGG-3**'  **GCTGATGCCTTCC** | **7911** |
|  | **B** |  |  |
| **15** | **A** | **5**'**-CGACTCTACGGAAGG-3'**  **GCTGAGATGCCTTCC** | **9146** |
|  | **B** |  |  |
| **20** | **A** | **5**'**-CGACTCTACGGCATCTGCGC-3**'  **GCTGAGATGCCGTAGACGCG** | **12237** |
|  | **B** |  |  |
| **25** | **A** | **5**'**-CGACTCTACGGAAGGGCATCTGCGC-3**'  **GCTGAGATGCCTTCCCGTAGACGCG** | **15327** |
|  | **B** |  |  |
| **30** | **A** | **5**'**-CGACTCTACGGAAGGTCTCGGACTACGCGC-3**'  **GCTGAGATGCCTTCCAGAGCCTGATGCGCG** | **18417** |
|  | **B** |  |  |
| **35** | **A** | **5**'**-CGACTCTACGGAAGGGCATCTCTCGGACTACGCGC-3**' **GCTGAGATGCCTTCCCGTAGAGAGCCTGATGCGCG** | **21507** |
|  | **B** |  |  |
| **Tethered Duplex** | **A** | **5**'**-GGTGACGAGTGA-TTT-GCTACTGGGCGG-3**'  **CCACTGCTCACT CGATGACCCGCC** | **14718** |
|  | **B** |  |  |

The sequences of the model DNA duplexes used in this study. Single strands are labeled A (sense) and B (antisense) for each DNA duplex. The eight pyrimidine‑purine dinucleotide sites in the 35 base‑pair duplex are underlined.

## Table S2:

| **Ruler** | **DNA length**  **(base steps)** | **Mean**  **(Å)** | **Variance**  **(Å^2^)** | **Bending Correction** | **Corrected**  **Mean** |
| --- | --- | --- | --- | --- | --- |
|  |  |  |  |  |  |
| XSI | 9 | 56.1 | 8.7 | 0.34 | 56.4 |
| XSI | 14 | 69.3 | 15.8 | 0.77 | 70.1 |
| XSI | 19 | 86.1 | 21.2 | 1.37 | 87.5 |
| XSI | 24 | 101.2 | 28.4 | 2.15 | 103.4 |
| XSI | 29 | 118.3 | 42.4 | 3.10 | 121.4 |
| XSI | 34 | 131.1 | 49.4 | 4.22 | 135.3 |
|  |  |  |  |  |  |
| trsmFRET | 7 | 40.4 | 17.3 | 0.22 | 40.6 |
| trsmFRET | 12 | 54.7 | 63.5 | 0.58 | 55.3 |
| trsmFRET | 17 | 66.9 | 123.8 | 1.11 | 68.0 |
| trsmFRET | 22 | 79.7 | 261.6 | 1.82 | 81.5 |
| trsmFRET | 27 | 92.3 | 289.8 | 2.70 | 95.0 |
|  |  |  |  |  |  |
| DEER | 0 | 26.4 | 2.9 | 0 | 26.4 |
| DEER | 1 | 27.0 | 3.6 | 0.01 | 27.0 |
| DEER | 1 | 27.0 | 4.4 | 0.01 | 27.0 |
| DEER | 3 | 21.2 | 6.3 | 0.05 | 21.3 |
| DEER | 6 | 22.6 | 15.2 | 0.17 | 22.8 |
| DEER | 7 | 26.1 | 13.0 | 0.22 | 26.3 |
| DEER | 8 | 31.8 | 19.4 | 0.28 | 32.1 |
| DEER | 9 | 38.8 | 25.0 | 0.34 | 39.1 |

Mean length and length variance of DNA duplexes measured by three different molecular rulers. XSI: X‑ray scattering interference. trsmFRET: time-resolved single‑molecule fluorescence resonance energy transfer. DEER: double electron‑electron spin resonance. In order to fit a helical rise value, the mean distances were corrected for the apparent shortening caused by DNA bending. The bending corrections were calculated using the analytical formula of Schurr and Fujimoto [11].

# References

1. Schaaff TG, Knight G, Shafigullin MN, Borkman RF, Whetten RL (1998) Isolation and selected properties of a 10.4 kDa gold:glutathione cluster compound. *J. Phys. Chem. B* **102:**10643-10646.

2. Laurence TA, Kong X, Jager M, Weiss S (2005) Probing structural heterogeneities and fluctuations of nucleic acids and denatured proteins. *Proc. Natl. Acad. Sci. USA* **102:**17348-17353.

3. Tsai C-C, Jain SC, Sobell HM (1975) X-ray crystallographic visualization of drug nucleic acid intercalative binding: structure of an ethidium-dinucleoside monophosphate crystalline complex, ethidium:5-Iodouridylyl(3'-5')Adenosine. *Proc. Natl. Acad. Sci. USA* **72:**628-632.

4. Davies DB, Karawajew L, Veselkov AN (1995) ^1^H-NMR structural analysis of ethidium bromide complexation with self‑complementary deoxytetranucleotides 5'‑d(ApCpGpT), 5'-d(ApGpCpT), and 5'‑d(TpGpCpA) in aqueous solution. *Biopolymers* **38:**745-757.

5. Kastrup RV, Young MA, Krugh TR (1978) Ethidium bromide complexes with self‑complementary deoxytetranucleotides. Demonstration and discussion of sequence preferences in the intercalative binding of ethidium bromide. *Biochemistry* **17:**4855‑4865.

6. Graves DE, Krugh TR (1983) Single‑cell partition analysis—A direct fluorescence technique for examining ligand‑macromolecule interactions. *Anal. Biochem.* **134:**73-81.

7. Nelson JW, Tinoco Jr. I (1984) Intercalation of ethidium ion into DNA and RNA oligonucleotides. *Biopolymers* **23:**213-233.

8. Mathew-Fenn RS, Das R, Harbury PAB (2008) *Science* Remeasuring the double helix. In press*.*

9. Fenn TD, Ringe D, Petsko GA (2003) POVScript+: a program for model and data visualization using persistence of vision ray-tracing. *J. Appl. Cryst.* **36:**944.

10. Cai Q, Kusnetzow AK, Hubbell WL, Haworth IS, Gacho GPC, et al. (2006) Site‑directed spin labeling measurements of nanometer distances in nucleic acids using a sequence‑independent nitroxide probe. *Nucl. Acids Res.* **34:**4722-4730.

11. Schurr JM, Fujimoto BS (2000) The distribution of end-to-end distances of the weakly bending rod model. *Biopolymers* **54:**561.
