# Supplementary material for: A Molecular Ruler for Measuring Quantitative Distance Distributions
Source: PLoS One. 2008 Oct 17;3(10):e3229. doi: 10.1371/journal.pone.0003229 (PMC2566812; doi:10.1371/journal.pone.0003229)
Supplement: Supplementary Materials S2 — MATLAB Data Analysis Scripts (2.42 MB ZIP) [file pone.0003229.s002.zip › Data for PLoS ONE/Instructions_README/MemSys5_manual.pdf]

Quantified Maximum Entropy

MemSys5

Users' Manual

This manual describes the `MemSys5` package of FORTRAN 77 subroutines designed to find and use maximum entropy reconstructions of images, power spectra, and other such additive distributions from arbitrary sets of data.

S.F. Gull and J. Skilling
Maximum Entropy Data Consultants Ltd.
South Hill
42 Southgate Street
Bury St. Edmunds
Suffolk, IP33 2AZ, U.K.

Version 1.2
September 8, 1999
Copyright © MEDC 1990—1999

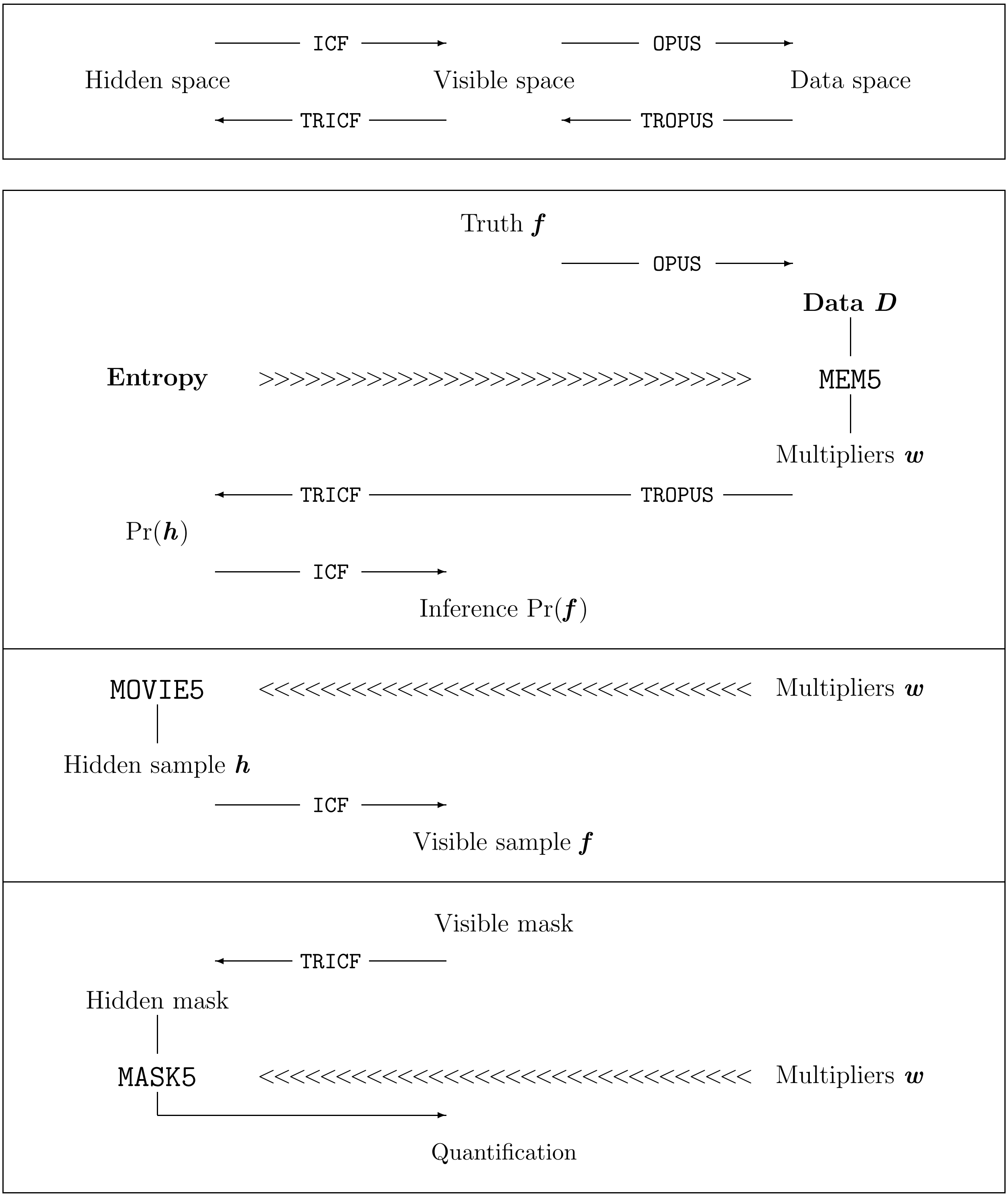
ICF
OPUS
Hidden space
Visible space
Data space
TRICF
TROPUS
Truth f
OPUS
Data D
Entropy
MEM5
Multipliers w
TRICF
TROPUS
Pr(h)
ICF
Inference Pr(f)
MOVIE5
Multipliers w
Hidden sample h
ICF
Visible sample f
Visible mask
TRICF
Hidden mask
MASK5
Multipliers w
Quantification

Contents

Introduction **7**

I Theory **9**

1 Probability theory and Bayes' Theorem **11**
1.1 Bayesian calculus for scientific inference 11
1.2 Entropic prior for a distribution 13
1.3 Gaussian likelihood 15

2 Classic maximum entropy **17**
2.1 Classic maximum entropy 17
2.2 Inferences about the reconstruction 24
2.3 Basic iterative algorithm 26
2.4 The intrinsic correlation function 29
2.5 Predicted data 29
2.6 Inferences about the noise level and other variables 30

3 Extensions and generalisations **33**
3.1 Positive/negative distributions 33
3.2 Fermi–Dirac distributions 34
3.3 Quadratic entropy 34
3.4 Fixed total 34

4 Algorithmic details **35**
4.1 Iterative algorithm in hidden space 35
4.2 Vector coding 36
4.3 Data-space algorithm 38

II Practice **41**

5 The `MemSys5` software package **43**

6 Storage areas **45**
6.1 Area structure 45
6.2 Initialisation: `MEINIT` 47
6.3 Area handling and overlaying 47

7 The vector library **49**
7.1 Area access routines VFETCH and VSTORE . . . 50
7.2 Disc-handling routines UFETCH and USTORE . . . 52
7.3 Arithmetical vector routines . . . 53

8 Transform routines **55**
8.1 Transform subroutines OPUS, TROPUS, and UMEMX . . . 55
8.2 Intrinsic correlation function routines ICF and TRICF . . . 57

9 Diagnostics and utilities **59**
9.1 Diagnostics routine UINFO . . . 59
9.2 Transform checking subroutine MEMTRQ . . . 60
9.3 Save and restore utility: MESAVE and MEREST . . . 61
9.4 Error messages . . . 61
9.5 Miscellaneous subroutines . . . 63

10 Specifications of the major subroutines **65**
10.1 MEMSET arguments . . . 65
10.2 MEM5 arguments . . . 66
10.3 MOVIE5 arguments . . . 69
10.4 MASK5 arguments . . . 71

11 Example of simple use **73**
11.1 Example of classic maximum entropy . . . 75
11.2 Post script . . . 84

12 Helpful hints **87**
12.1 Multiple maxima of α . . . 87
12.2 Tolerance variable UTOL . . . 87
12.3 Internal iteration limits . . . 88
12.4 Arithmetic accuracy . . . 88
12.5 Combination of transforms . . . 88

A User-supplied routines **91**

B Historic maximum entropy **93**

C Reserved names **95**

D Changes from MemSys2 to MemSys3 **97**

E Changes from MemSys3 to MemSys5 **99**

List of Figures

2.1 Maximum entropy trajectory. 20
2.2 Maximum entropy cloud. 21
2.3 Maximum entropy cloud. 21
2.4 Maximum entropy cloud. 22
2.5 Maximum entropy cloud. 22
2.6 Maximum entropy cloud. 23
2.7 Maximum entropy cloud. 23
2.8 Integrated maximum entropy cloud. 24

11.1 Noisy blurred data (`gauss.dat`). 74
11.2 Classic MaxEnt reconstruction $\widehat{\boldsymbol{f}}$ 80
11.3 Classic MaxEnt reconstruction $\widehat{\boldsymbol{h}}$ 80
11.4 Sample from posterior cloud 81
11.5 Sample from posterior cloud 81
11.6 Sample from posterior cloud 82
11.7 Sample from posterior cloud 82
11.8 The true simulation $\boldsymbol{f}$ 84

B.1 Historic maximum entropy. 94

List of Tables

6.1 MemSys5 area usage. 46

9.1 Coding of INFO calls with MLEVEL. 59

11.1 MASK5 quantification results 83
11.2 Comparison of estimated and true values for the simulation of Figure 11.2. 85

Introduction

The origins of maximum entropy analysis can be traced back to the 18th century, to the works of Bernoulli and Laplace, and brought to our own day through the works of Jeffreys and Jaynes. Today, it is widely recognised as a fundamental tool of scientific inference. The `MemSys5` package is the most advanced software available for maximum entropy analysis, and has been developed by Maximum Entropy Data Consultants Ltd. as a result of more than 10 years' research and development. Over this period, extensive experience has been gained in the application of these techniques to practical problems in commercial, academic, and government organisations around the world.

Problems involving the reconstruction of distributions occur in many branches of science and engineering, and many techniques have been developed over the years to try to deal with them. One of the reasons for the enormous success of maximum entropy in an ever-increasing number of disciplines is that it is not an *ad hoc* technique, but is founded instead on the bedrock of probability theory. Thus the principles involved are universally applicable, and, if properly applied, allow the most efficient possible use of the data in the inference process.

One of the major benefits of an analysis based on probability theory is that it provides a mechanism for handling 'noisy' and sparse data in an entirely rational and consistent manner. The result of the analysis is not just a single 'best estimate' of the quantity of interest, but includes a precise statement of the confidence which can be placed in it. The `MemSys5` package provides two tools for investigating and quantifying the accuracy of these estimates. Firstly, it provides a general-purpose routine which can be used to give a dynamic, visual illustration of the way in which the uncertainties in the data allow uncertainties in the results. Secondly, it provides a routine for quantifying how accurately specific features of the reconstructed distributions are determined. The `MemSys5` package is written in FORTRAN 77, but a machine-translated version of the source code in C can be supplied instead.

This manual describes the theory and practice of maximum entropy data analysis as implemented in the `MemSys5` package. Part I summarises the derivation of the maximum entropy formalism from the basic laws of probability theory, and describes the algorithms used in the package. Part II contains a detailed description of the software itself. The `MemSys5` package is a maximum entropy 'inference engine'; to apply this 'engine' to a specific problem, a set of interface routines is required to model the relationship between the data and the distribution of interest. The specifications for these interface routines are given, together with examples. The package is distributed with a complete, working "`TOY`" deconvolution program as a simple illustration of its use.

Part I

Theory

Chapter 1

Probability theory and Bayes' Theorem

1.1 Bayesian calculus for scientific inference

Bayesian probability calculus is the natural, and indeed the only, language for drawing inferences in a consistent fashion. Each of the hypotheses A, B, C, ... which might be assessed in an experiment corresponds to a logical proposition of the form "X is true", where X stands for any of A, B, C, ... It follows that scientific data analysis is a special application of our methods for dealing with logical propositions.

Let us suppose that there is a general calculus for dealing with such propositions. If there is, then obviously it must apply in special cases. Cox (1946) proved that any calculus which is consistent with the ordinary rules of Boolean algebra obeyed by propositions must be equivalent to Bayesian probability theory. Each proposition P carries a numerical code $\Pr(P)$, obeying

$$\Pr(P, Q) = \Pr(P)\Pr(Q \mid P)$$
$$\Pr(P) + \Pr(\overline{P}) = 1$$

where "$\,,\,$" means "and", "$\,|\,$" means "given", and "$\,\overline{}\,$" means "not". All such codes lie between 0 and 1, with the particular propositions "true" and "false" having the extreme values

$$\Pr(\text{false}) = 0, \qquad \Pr(\text{true}) = 1\ .$$

These are the standard rules of probability calculus, and accordingly the codes are called "probabilities". The only freedom allowed is to rescale the Bayesian probabilities by some arbitrary monotonic function, as percentages are obtained by multiplying by 100. Whatever codes were first assigned to the propositions, these could be mapped uniquely back to the Bayesian probability values, so that we may adopt this standard convention without any loss of generality.

This definition of "probability" is, of course, consistent with the commonly used definition as a limiting frequency ratio of "successes/trials". Frequency experiments are a compelling simple case for which any general calculus ought to give the obvious rules derived from multiplication and addition of (large) integers. Bayesian calculus passes this test. Moreover, the Cox definition of probability is more general than the frequentist definition, because it refers to arbitrary propositions without having to invent a "many-worlds" scenario involving all the events which might have happened but did not.

Conceivably, there may be no general language at all. One can imagine the existence of some other compelling simple case for which Bayesian calculus might give a demonstrably "wrong" result. We cannot prove that such counter-examples do not exist. However, it is difficult to see how one might be constructed. Despite much effort, nobody has yet found one, and so we shall adopt a rigorous Bayesian approach.

In scientific data analysis, we wish to use our data $\boldsymbol{D}$ to make inferences about various possible hypotheses A, B, C, ... We have no rational alternative but to code this inference in terms of conditional probabilities

$$\Pr(A \mid \boldsymbol{D}), \quad \Pr(B \mid \boldsymbol{D}), \quad \Pr(C \mid \boldsymbol{D}), \quad \ldots$$

5 Using "h" to represent any particular hypothesis $A, B, C, \ldots$, we need the probability density

$$\Pr(h \mid \boldsymbol{D})$$

as a function of h.

The data $\boldsymbol{D}$ do not give us this directly. Instead, the data give us the **likelihood**

$$\Pr(\boldsymbol{D} \mid h)$$

as a function of h. The particular algebraic form of the likelihood may involve delta functions for exact data, or Gaussian factors if the data have normally-distributed noise, and may include various correlations and other peculiarities of the observing system which produced the data. Just about the simplest example would be a single measurement

$$D = h \pm \sigma$$

of a single number h, which (assuming normally distributed error) is just a convenient shorthand for

$$\Pr(D \mid h) = (2\pi\sigma^2)^{-1/2} \exp(-(D-h)^2/2\sigma^2).$$

Another common example concerns data which are integer counts with mean h, subject to Poisson statistics. In that case,

$$\Pr(D \mid h) = e^{-h}\, h^D / D!$$

In order to reverse the conditioning from $\Pr(\boldsymbol{D} \mid h)$ to the required $\Pr(h \mid \boldsymbol{D})$ we use Bayes' theorem. A useful trick for navigating through the morass of possible algebraic manipulations when dealing with multiple propositions is to start with the joint probability density of everything relevant, and expand it in various ways. Here we have

Prior (↓ $\Pr(h)$) *Likelihood* (↓ $\Pr(\boldsymbol{D} \mid h)$)

$$\boxed{\begin{aligned} \Pr(h, \boldsymbol{D}) &= \Pr(h) \quad \Pr(\boldsymbol{D} \mid h) \\ &= \Pr(\boldsymbol{D}) \quad \Pr(h \mid \boldsymbol{D}) \end{aligned}}$$

Evidence (↑ $\Pr(\boldsymbol{D})$) *Inference* (↑ $\Pr(h \mid \boldsymbol{D})$)

which is the basic tool of Bayesian data analysis. Basically, we seek the inference

$$\Pr(h \mid \boldsymbol{D}) = \Pr(h)\ \Pr(\boldsymbol{D} \mid h)/\Pr(\boldsymbol{D})$$

about h (Bayes' Theorem), but the evidence

$$\Pr(\boldsymbol{D}) = \sum_h \Pr(h, \boldsymbol{D})$$

is hardly less important. In order to calculate these quantities, we need the "prior probability" density $\Pr(h)$ as well as the experimental likelihood. This prior codifies our prior expectations about possible results h *before* acquiring the new data $\boldsymbol{D}$. Probability theory tells us how to use data to *modulate* this prior expectation into a posterior inference, but it does *not* tell us how to assign the prior expectation in the first place. Actually, this is quite reasonable: a language should not impose on what one wishes to say, and it leaves open the question of assigning the prior.

Different analysts may legitimately disagree on their choice of prior. However, the evidence lets us discriminate objectively between them. Each choice X, Y, ... of prior leads to a numerical evidence $\Pr(\boldsymbol{D})$, more properly written now as $\Pr(\boldsymbol{D} \mid X)$, $\Pr(\boldsymbol{D} \mid Y)$, ... Unless we have particular grounds for favouring one choice over another, our inferences $\Pr(X \mid \boldsymbol{D})$, $\Pr(Y \mid \boldsymbol{D})$, ... about the available choices will follow the evidence values in proportion. Indeed, the evidence discriminates between priors in exactly the same way that the likelihood discriminates between hypotheses. In principle, one perhaps ought to average over the different choices of prior, but in practice the discrimination is usually good enough that we may simply choose the "best" available prior, on the basis of the evidence, and ignore the others.

1.2 Entropic prior for a distribution

Many distributions h of interest in science are positive and additive. For example, the intensity of light across an image is positive (obviously) and additive (meaning that the flux received across two non-overlapping patches is the sum of the individual fluxes). Likewise, a power spectrum is positive (obviously) and additive (meaning that the net power in two non-overlapping bands is the sum of the powers in the individual bands). We call a positive and additive distribution a **PAD**.

We seek the prior density of h

$$\Pr(h)$$

where each individual h now takes the form of a positive additive function h of position $\boldsymbol{x}$ (defined in one or more dimensions).

$$h(\boldsymbol{x}) = \text{density at position } \boldsymbol{x}$$

so that

$$\int h(\boldsymbol{x})\, d\boldsymbol{x} = \text{quantity within the range of integration.}$$

The corresponding discrete form on L cells $i = 1, 2, \ldots, L$ is

$$\Pr(\boldsymbol{h})$$

where

$$h_i = \text{quantity in cell } i$$

so that

$$\sum h_i = \text{quantity within the range of summation}$$

tends to the continuous form as the cells shrink and $L \to \infty$.

As a step towards finding $\Pr(\boldsymbol{h})$, we consider the easier problem of assigning a "best" $\boldsymbol{h}$. Later, we shall identify this with the most probable $\boldsymbol{h}$, and thus gain information about $\Pr(\boldsymbol{h})$ itself. Following the Cox mode of reasoning, we suppose that there is a general prior $\Pr(\boldsymbol{h})$, and hence a generally valid rule for assigning the most probable $\boldsymbol{h}$. If there is, it must be valid for special types of data, and in particular the most probable selection must also be valid for such data. We shall use the "kangaroo" argument (Gull and Skilling 1984a):

> If the proportion of some entity which has a given property is known to be p, then the most probable estimate of the proportion in some subclass which has that property is (in the absence of any information connecting the subclass with the property) the same number p.

For example, if 30% of kangaroos are left-handed, then the most probable estimate of the proportion of kangaroos in Queensland which are left-handed is also 30% (unless more is known about the handedness of Queensland kangaroos).

Remarkably, the consequence of this apparently weak requirement (Shore and Johnson 1980, Tikochinsky, Tishby and Levine 1984, Gull and Skilling 1984a,b) is that the "best" set of proportions p_i ($i = 1, 2, \ldots, L$) on L *a priori* equivalent cells must be obtained by maximising the entropy

$$S(\boldsymbol{p}) = -\sum_{i=1}^{L} p_i \log p_i$$

No other function will always give the required uncorrelated form of "best" proportions. This result is slightly restrictive in that proportions must sum to 1, whereas more general positive additive distributions need not. In fact, the only acceptable generalisation of this (to PADs $\boldsymbol{h}$ which need not add to 1) is to select $\boldsymbol{h}$ by maximising the **entropy**

$$S(\boldsymbol{h}) = \sum_{i=1}^{L} (h_i - m_i - h_i \log(h_i/m_i)) \tag{1.1}$$

where m_i is the measure assigned to cell i (Skilling 1988). In the continuum limit the entropy becomes

$$S(h) = \int d\boldsymbol{x}\, (h(\boldsymbol{x}) - m(\boldsymbol{x}) - h(\boldsymbol{x}) \log(h(\boldsymbol{x})/m(\boldsymbol{x}))).$$

The global maximum of S is at $h = m$ (where $S = 0$), so that the (negative) value of S measures the deviation of h from the assigned measure m. Often, m may be set to a constant, perhaps justified by an appeal to spatial invariance. More generally, $m(\boldsymbol{x})$ can be used as an initial or "default" **model** for the distribution h, to which h will default in the absence of data.

Identifying the "best" h with the most probable, this result shows that the most probable h under definitive "testable" constraints (Jaynes 1978) is to be found by maximising the entropy of h. Applied directly to the assignment of probability densities, this prescription is the Principle of Maximum Entropy (Jaynes 1978), here separated from its usual derivation in terms of counting large numbers of states. However, our problem is more difficult: we do not wish merely to assign an h—we have to determine a full prior probability density $\Pr(h)$.

If the most probable h is always to be found by maximising S, then $\Pr(h)$ can only be some as yet unknown but monotonically increasing function

$$\Pr(h) = \Phi(\, S(h)\,).$$

To find Φ we need some quantified special case which is consistent with the selection requirement above and for which $\Pr(h)$ is known. We use the following "monkey" argument (Gull and Daniell 1978):

> If a team of monkeys throws a very large number N of quanta randomly at the L *a priori* equivalent cells of a distribution, then the probability of obtaining a particular set $(n_1, n_2, \ldots, n_L)$ of occupation numbers shall be proportional to the degeneracy $N!/n_1!n_2!\ldots n_L!$

Of course, we do not suppose that distributions of interest have to be formed in this way; we merely remark that we would like to obtain the right answer in that special case. The consequence of this argument (Skilling and Gull 1989, Skilling 1989) is that Φ must be of exponential form

$$\Phi(S) \propto \exp(\alpha S)$$

where α is some constant. A full treatment yields also the diagonal metric tensor $[\boldsymbol{g}] = -\nabla\nabla S$ and the corresponding invariant volume element $(\det[\boldsymbol{g}])^{1/2} d^L h$ which should be assigned to the space of distributions $\boldsymbol{h}$. Here, and henceforward, we use square brackets [ ] to denote a diagonal matrix. For later convenience, we rewrite the metric tensor in contravariant form as

$$[\boldsymbol{\mu}] = [\boldsymbol{g}]^{-1} = (-\nabla\nabla S)^{-1}$$

whence the invariant volume element becomes $d^L h / \det[\boldsymbol{\mu}]^{1/2}$. The final result is that

$$\Pr(\boldsymbol{h} \in V \mid \alpha) = \frac{1}{Z_S(\alpha)} \int_V \exp(\alpha S(\boldsymbol{h}))\, d^L h / \det[\boldsymbol{\mu}]^{1/2}$$

where V is a domain of PADs $\boldsymbol{h}$, and

$$Z_S(\alpha) = \int_\infty \exp(\alpha S(\boldsymbol{h}))\, d^L h / \det[\boldsymbol{\mu}]^{1/2}$$

is the scaling constant required to ensure that $\Pr(\boldsymbol{h})$ is properly normalised.

We have now determined the prior completely, apart from the single number α which remains unknown. Because S is dimensional (having the units of total $\boldsymbol{h}$) and the exponential must have dimensionless argument, it follows that α too must be dimensional, having the inverse units to total $\boldsymbol{h}$. Accordingly, α cannot be determined *a priori*, and the prior analysis can go no further.

If there is a general prior, it must be the one derived here. Conceivably, there may be no general prior at all. One can imagine the existence of some other compelling simple case for which this prior might be demonstrably "wrong". However, such other cases as have been investigated, such as the geometrical arguments of Levine (1986) and Rodríguez (1989), serve to confirm this functional form, and might indeed be developed into alternative derivations of it. The coefficient α is known as the "regularisation constant" by analogy with statistical regularisation theory.

1.3 Gaussian likelihood

Noise in experimental apparatus is often adequately described by normal statistics. Specifically, we then take the probability density for errors on a series of N measurements D_k (for $k = 1, 2, \ldots, N$) to be Gaussian, with a covariance matrix $[\boldsymbol{\sigma}^{-2}]$, so that the likelihood is

$$\Pr(\boldsymbol{D} \mid \boldsymbol{h}) = \frac{e^{-L(\boldsymbol{h})}}{Z_L}$$

where

$$Z_L = \int e^{-L(\boldsymbol{h})}\, d^N D,$$

and

$$\begin{aligned} L(\boldsymbol{h}) &= \frac{1}{2}(\boldsymbol{D} - \boldsymbol{F}(\boldsymbol{h}))^T [\boldsymbol{\sigma}^{-2}]\, (\boldsymbol{D} - \boldsymbol{F}(\boldsymbol{h})) \\ &= \frac{1}{2}\chi^2(\boldsymbol{h}) \end{aligned} \tag{1.2}$$

and $\boldsymbol{F}(\boldsymbol{h})$ are the data predicted from the hidden-space distribution $\boldsymbol{h}$, so that

$$Z_L = (2\pi)^{N/2} / \det[\boldsymbol{\sigma}^{-1}].$$

We take the covariance matrix $[\boldsymbol{\sigma}^{-2}]$ to be diagonal, as from independent errors. Although these assumptions are not essential, they facilitate our analysis.

Chapter 2

Classic maximum entropy

2.1 Classic maximum entropy

Classic maximum entropy uses the Bayesian formulation just described to produce the posterior **probability cloud** $\Pr(\boldsymbol{h} \mid \boldsymbol{D})$.

The full joint probability density can be expanded as

$$\Pr(\boldsymbol{h}, \alpha, \boldsymbol{D}) = \Pr(\alpha)\ \Pr(\boldsymbol{h} \mid \alpha)\ \Pr(\boldsymbol{D} \mid \boldsymbol{h}, \alpha)$$

Taking these factors in reverse order, we can drop the conditioning on α in $\Pr(\boldsymbol{D} \mid \boldsymbol{h}, \alpha)$ because it is $\boldsymbol{h}$ itself which induces the data. Thus $\Pr(\boldsymbol{D} \mid \boldsymbol{h}, \alpha) = \Pr(\boldsymbol{D} \mid \boldsymbol{h}) = \exp(-\mathcal{L}(\boldsymbol{h}))/Z_{\mathcal{L}}$ for Gaussian errors. Next, the factor $\Pr(\boldsymbol{h} \mid \alpha)$ is the entropic prior $\exp(\alpha\mathcal{S}(\boldsymbol{h}))/Z_{\mathcal{S}}(\alpha)$. We use the notation $\mathcal{S}(\boldsymbol{h})$ and $\mathcal{L}(\boldsymbol{h})$ to indicate that the maximum entropy formalism can be extended to accommodate forms other than (1.1) and (1.2) on pages 14 and 16 for $S(\boldsymbol{h})$ and $L(\boldsymbol{h})$. Alternative entropy forms must, however, like the standard form 1.1, be convex functions of $\boldsymbol{h}$ with a global maximum value of zero. This will be illustrated in Chapter 3. Hence

$$\begin{aligned}\Pr(\boldsymbol{h}, \alpha, \boldsymbol{D}) &= \Pr(\alpha)\ \Pr(\boldsymbol{h} \mid \alpha)\ \Pr(\boldsymbol{D} \mid \boldsymbol{h}) \\ &= \Pr(\alpha)\,\frac{\exp(\alpha\mathcal{S}(\boldsymbol{h}) - \mathcal{L}(\boldsymbol{h}))}{Z_{\mathcal{S}}(\alpha)\ Z_{\mathcal{L}}}. \qquad (2.1)\end{aligned}$$

If $\mathcal{L}(\boldsymbol{h})$ is a quadratic function of $\boldsymbol{h}$, then for each α there is necessarily (because $\alpha\mathcal{S}(\boldsymbol{h}) - \mathcal{L}(\boldsymbol{h})$ is also convex in $\boldsymbol{h}$) a single maximum of (2.1) over $\boldsymbol{h}$ at $\widehat{\boldsymbol{h}}(\alpha)$, where $\widehat{\boldsymbol{h}}$ obeys

$$\alpha\frac{\partial\mathcal{S}}{\partial\boldsymbol{h}} = \frac{\partial\mathcal{L}}{\partial\boldsymbol{h}}.$$

Gaussian approximation

Further analysis using the exact form (2.1) appears to be algebraically and numerically intractable, and so we form a Gaussian approximation about $\widehat{\boldsymbol{h}}$. The Hessian matrix of $\alpha\mathcal{S} - \mathcal{L}$ is

$$\begin{aligned}-\frac{\partial^2}{\partial\boldsymbol{h}\,\partial\boldsymbol{h}}\Big(\alpha\mathcal{S}(\boldsymbol{h}) - \mathcal{L}(\boldsymbol{h})\Big) &= -\alpha\nabla\nabla\mathcal{S} + \nabla\nabla\mathcal{L} \\ &= [\boldsymbol{\mu}^{-1/2}]\ \Big(\alpha\boldsymbol{I} + [\boldsymbol{\mu}^{1/2}]\,\nabla\nabla\mathcal{L}\,[\boldsymbol{\mu}^{1/2}]\Big)\ [\boldsymbol{\mu}^{-1/2}] \\ &= \alpha\,[\boldsymbol{\mu}^{-1/2}]\,\boldsymbol{B}\,[\boldsymbol{\mu}^{-1/2}]\end{aligned}$$

where

$$\begin{aligned} \boldsymbol{B} &= \boldsymbol{I} + \boldsymbol{A}/\alpha \\ \boldsymbol{I} &= \text{identity matrix}, \quad \text{and} \quad \boldsymbol{A} = [\boldsymbol{\mu}^{1/2}]\, \nabla\nabla\mathcal{L}\, [\boldsymbol{\mu}^{1/2}]. \end{aligned}$$

Hence the Gaussian approximation to (2.1) is

$$\Pr(\boldsymbol{h}, \alpha, \boldsymbol{D}) = \Pr(\alpha)\, \frac{\exp(\alpha\mathcal{S}(\widehat{\boldsymbol{h}}) - \mathcal{L}(\widehat{\boldsymbol{h}}))}{Z_{\mathcal{S}}\, Z_{\mathcal{L}}} \exp\left(-\frac{\alpha}{2}(\boldsymbol{h} - \widehat{\boldsymbol{h}})^T [\boldsymbol{\mu}^{-1/2}]\, \boldsymbol{B}\, [\boldsymbol{\mu}^{-1/2}]\, (\boldsymbol{h} - \widehat{\boldsymbol{h}})\right). \qquad (2.2)$$

The regularisation constant α

In order to investigate the rôle of α more closely, we integrate $\boldsymbol{h}$ out of (2.2), obtaining

$$\begin{aligned} \Pr(\alpha, \boldsymbol{D}) &= \int \Pr(\boldsymbol{h}, \alpha, \boldsymbol{D})\, d^L h / \det[\boldsymbol{\mu}]^{1/2} \\ &= \Pr(\alpha)\, Z_{\mathcal{L}}^{-1} \exp(\alpha\mathcal{S}(\widehat{\boldsymbol{h}}) - \mathcal{L}(\widehat{\boldsymbol{h}}))\, (\det \boldsymbol{B})^{-1/2}. \qquad (2.3) \end{aligned}$$

We now need to assign $\Pr(\alpha)$. Because α is a scale parameter, complete ignorance demands the improper Jeffreys prior $\Pr(\alpha) \propto \alpha^{-1}$. However, impropriety carries a large penalty, in that the evidence $\Pr(\boldsymbol{D}) = \int d\alpha\, \Pr(\alpha, \boldsymbol{D})$ can become arbitrarily small. In practice, users of Jeffreys priors cut them off outside some largish range, but even then there is a large penalty relative to an analyst who already knew the order of magnitude of α. It seems to us that this cost is too high.

With *theoretical* objectivity being impractical, we propose to recover objectivity by *convention.* In practice, one almost always knows the order of magnitude of what one is going to observe (to which α is inversely related). Accordingly, we recommend reducing both tails of the Jeffreys prior by single powers of α, to give the fully convergent form

$$\Pr(\alpha \mid \alpha_0) = \frac{2\alpha_0}{\pi(\alpha^2 + \alpha_0^2)} \qquad (\alpha > 0).$$

Although the purist would require α_0 to be fixed in advance, the pragmatist may observe that the experiment is likely to have been set up so that the inferred range of α will not be too far from α_0. Hence we recommend *choosing* α_0 *to maximise the evidence* $\Pr(\boldsymbol{D} \mid \alpha_0)$. This involves evaluating $\Pr(\alpha, \boldsymbol{D})$ for a sufficient range of α, and performing the integrals over α numerically. In this way, adequate objectivity can be retained, so that (for example) maximum entropy results could be compared with different Bayesian calculations (e.g., Skilling 1991).

With realistically large datasets, though, it is seldom necessary to attend to such subtleties. In most practical cases, the evidence $\Pr(\boldsymbol{D} \mid \alpha)$ is so strongly peaked that it overwhelms any plausible prior on α. We then just *select this "best" value* $\widehat{\alpha}$ and proceed on the assumption that α is then fixed.

Differentiation of (2.3) shows that the evidence is extremal over α when

$$-2\alpha\mathcal{S}(\widehat{\boldsymbol{h}}) = G, \qquad G = \operatorname{trace}((\alpha\boldsymbol{B})^{-1}\boldsymbol{A})$$

and maximal when $d(-2\alpha\mathcal{S}/G)/d\alpha > 0$ at the extremum.

Using the eigenvalues λ of $\boldsymbol{A}$ to write

$$G = \sum \frac{\lambda}{\lambda + \alpha}$$

gives G a natural interpretation as the number of "good" measurements. Each eigenvalue larger than α represents an informatively accurate independent measurement, which contributes roughly 1 to G. Conversely, each eigenvalue less than α represents an inaccurate measurement, which contributes roughly 0 to G. The most probable value of α occurs when the number of good measurements equals the dimensionless amount $-2\alpha\mathcal{S}(\widehat{\boldsymbol{h}})$ of structure in the distribution $\widehat{\boldsymbol{h}}$ (remember $\mathcal{S}$ is negative).

When α is small, G and $\mathcal{S}$ go to fixed limits, so that $-2\alpha\mathcal{S}/G < 1$. Conversely, when α is large, it is usually the case that the data are sufficiently informative to ensure $-2\alpha\mathcal{S}/G > 1$. In that case, some intermediate value of α will be the most probable. Although mildly unusual, it is perfectly possible for there to be more than one local maximum of $\Pr(\alpha \mid D)$, in which case the largest ought to be found and selected. It is also possible for the data to be sufficiently in accord with the assumed default model $\boldsymbol{m}$ that $\alpha = \infty$ is the most probable value, in which case the cloud for $\boldsymbol{h}$ collapses because there is no adequate evidence for any change from the model.

With this assignment of $\widehat{\alpha}$, the corresponding probability cloud

$$\Pr(\boldsymbol{h} \mid \boldsymbol{D}) = \text{constant} \times \exp\left(-\frac{\widehat{\alpha}}{2}(\boldsymbol{h} - \widehat{\boldsymbol{h}})^T[\boldsymbol{\mu}^{-1/2}]\,\boldsymbol{B}\,[\boldsymbol{\mu}^{-1/2}]\,(\boldsymbol{h} - \widehat{\boldsymbol{h}})\right) \tag{2.4}$$

is the maximum entropy result. It represents the inference about $\boldsymbol{h}$, conditional in particular on the default model $\boldsymbol{m}$. Accompanying it is the evidence

$$\begin{aligned}\Pr(\boldsymbol{D}) &= Z_{\mathcal{L}}^{-1}\,\exp(\widehat{\alpha}\mathcal{S}(\widehat{\boldsymbol{h}}) - \mathcal{L}(\widehat{\boldsymbol{h}}))\,(\det \boldsymbol{B})^{-1/2}\\ &= (2\pi)^{-N/2}\,\det[\boldsymbol{\sigma}^{-1}]\,\exp(\widehat{\alpha}\mathcal{S}(\widehat{\boldsymbol{h}}) - \mathcal{L}(\widehat{\boldsymbol{h}}))\,(\det \boldsymbol{B})^{-1/2}\end{aligned} \tag{2.5}$$

for Gaussian errors. The term $\det[\boldsymbol{\sigma}^{-1}]$ in (2.5) confirms that $\Pr(\boldsymbol{D})$ has the dimensions of $[\text{data}]^{-N}$.

The maximum entropy trajectory.

The solutions $\widehat{\boldsymbol{h}}$ for various values of α define the "maximum entropy trajectory", starting from $\widehat{\boldsymbol{h}} = \boldsymbol{m}$ at $\alpha = \infty$ and ending at minimum $\mathcal{L}$ when $\alpha = 0$. As a matter of practical computation, it is advantageous to start from $\widehat{\boldsymbol{h}} = \boldsymbol{m}$ and iterate down the trajectory until the **stopping criterion** is satisfied. We call the value of α at which this occurs the "stopping" value. It is usually time-consuming to recover from a PAD $\boldsymbol{h}$ which lies far from the trajectory.

A general feature of maximum entropy trajectories is that structure in $\boldsymbol{h}$ for which there is good evidence in the data is picked up first. Only later does the trajectory bend around to acquire structure for which there is progressively weaker evidence. This qualitative behaviour can be seen in Figure 2.1, which illustrates maximum entropy applied to finding a two-cell PAD $\boldsymbol{h} = (h_1, h_2)$ from the noisy data

$$\begin{aligned}0.56h_1 + 0.83h_2 &= 5.32 \pm 0.48,\\ 0.83h_1 - 0.56h_2 &= 4.24 \pm 2.00.\end{aligned}$$

Solid contours are of $\mathcal{L}$,

$$\mathcal{L}(\boldsymbol{h}) = \frac{1}{2}\left((0.56h_1 + 0.83h_2 - 5.32)^2/0.48^2 + (0.83h_1 - 0.56h_2 - 4.24)^2/2.00^2\right).$$

Dashed contours are of entropy

$$\mathcal{S}(\boldsymbol{h}) = h_1 + h_2 - m_1 - m_2 - h_1\log(h_1/m_1) - h_2\log(h_2/m_2) \quad \text{with} \quad m_1 = m_2 = 1.$$

The line of stars marks the trajectory of maxima of entropy over different values of $\mathcal{L}$, starting at the global maximum of entropy at $\boldsymbol{h} = \boldsymbol{m}$ and ending at the $\mathcal{L}$ minimum where the data are fitted as closely as possible (exactly, in this example). Because $\mathcal{S}$ is a strictly convex function of $\boldsymbol{h}$, and $\mathcal{L}$ is concave, each such maximum is necessarily unique.

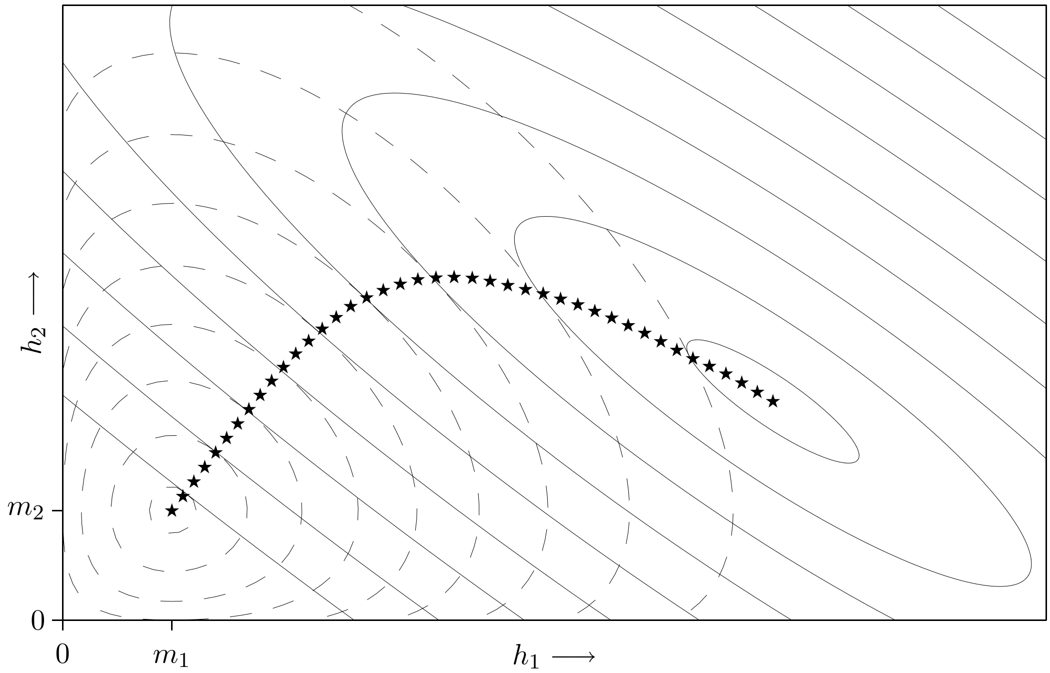

Figure 2.1: Maximum entropy trajectory.

Maximum entropy clouds.

Figures 2.2 to 2.7 illustrate classic maximum entropy clouds for the numerical example of Figure 2.1. For any given value of α, the posterior probability cloud of $\boldsymbol{h}$ takes a roughly Gaussian form centred at the corresponding point $\widehat{\boldsymbol{h}}(\alpha)$ of the maximum entropy trajectory. Each cloud is plotted as a scatter diagram of points randomly picked from the corresponding probability density $\exp(\alpha\mathcal{S}-\mathcal{L})$, and scaled for display to a constant number of samples.

Initially, at large α (Figure 2.2), the cloud is small, being tightly constrained by the entropy. Later, at smaller α, the cloud expands until (by about Figure 2.5) the accurate data have been fitted and the entropy exerts relatively little effect in such directions. At yet smaller values of α, less accurate data become fitted until ultimately (at $\alpha = 0$) the cloud fits all the data (in so far as this is consistent with positivity of $\boldsymbol{h}$) and is defined purely by $\mathcal{L}$ (Figure 2.7).

The most probable cloud is close to that in Figure 2.6, so that there happens to be rather little entropic regularisation in this small-scale problem.

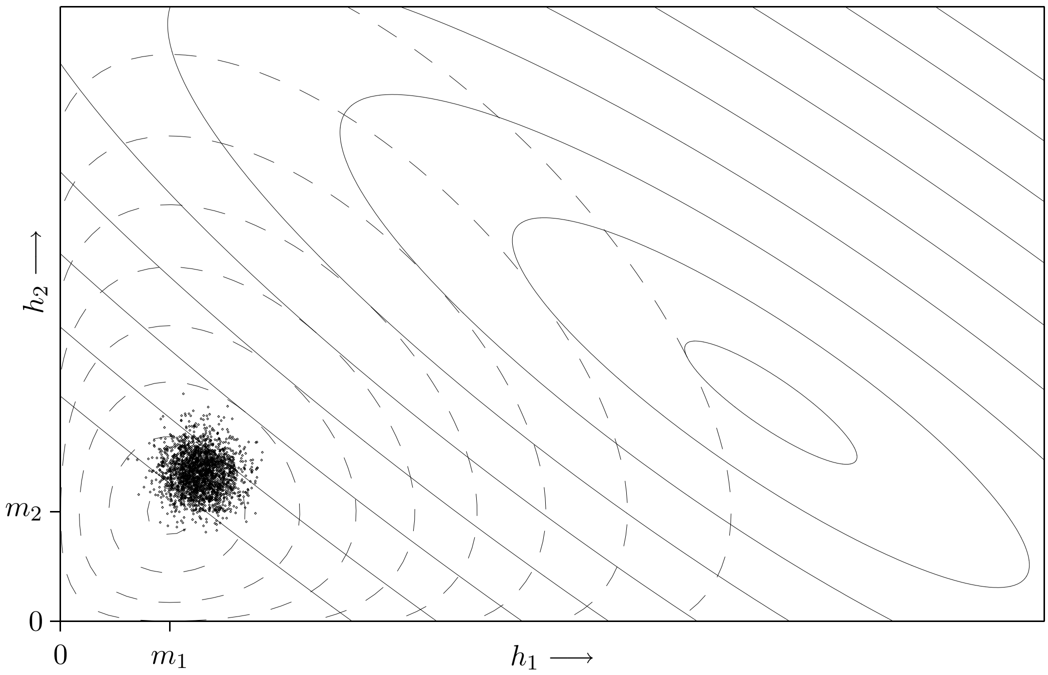

Figure 2.2: Maximum entropy cloud.

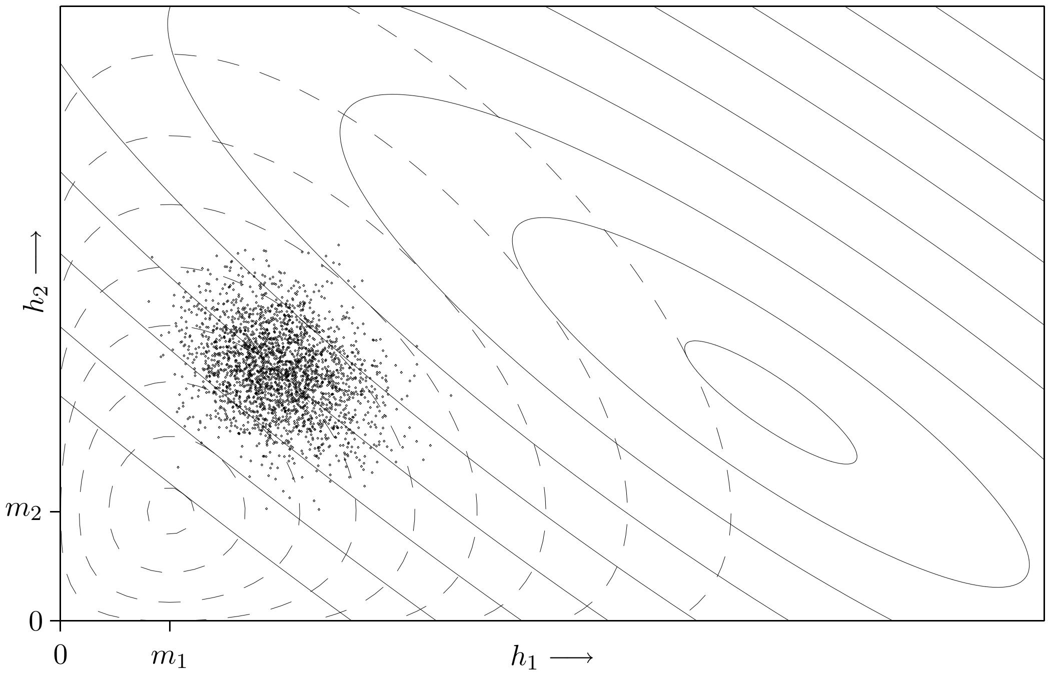

Figure 2.3: Maximum entropy cloud.

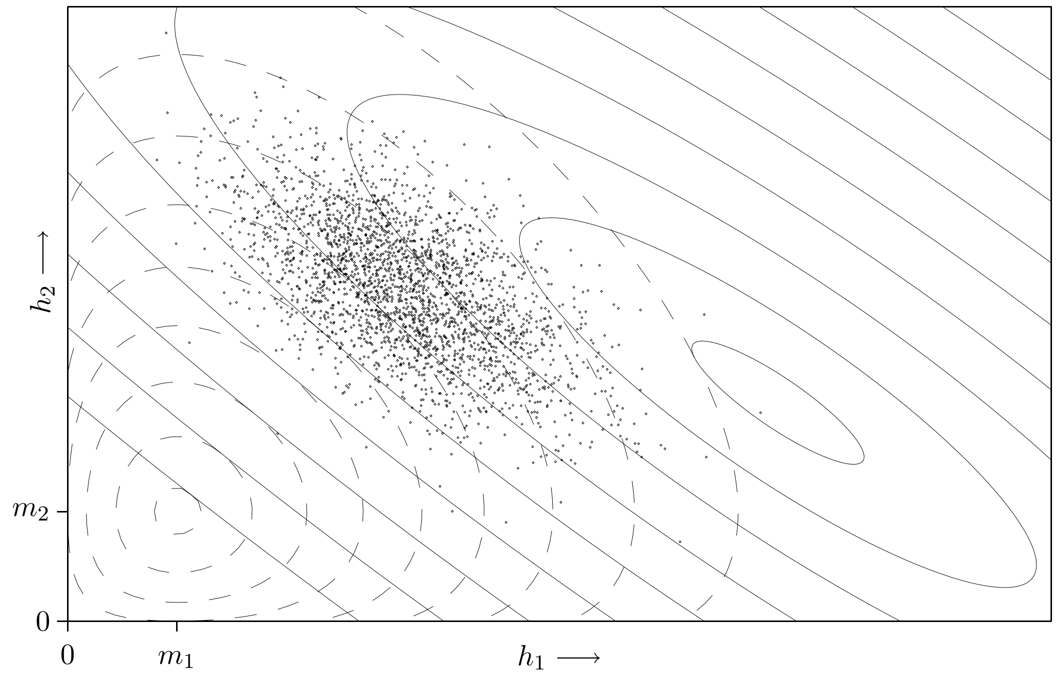

Figure 2.4: Maximum entropy cloud.

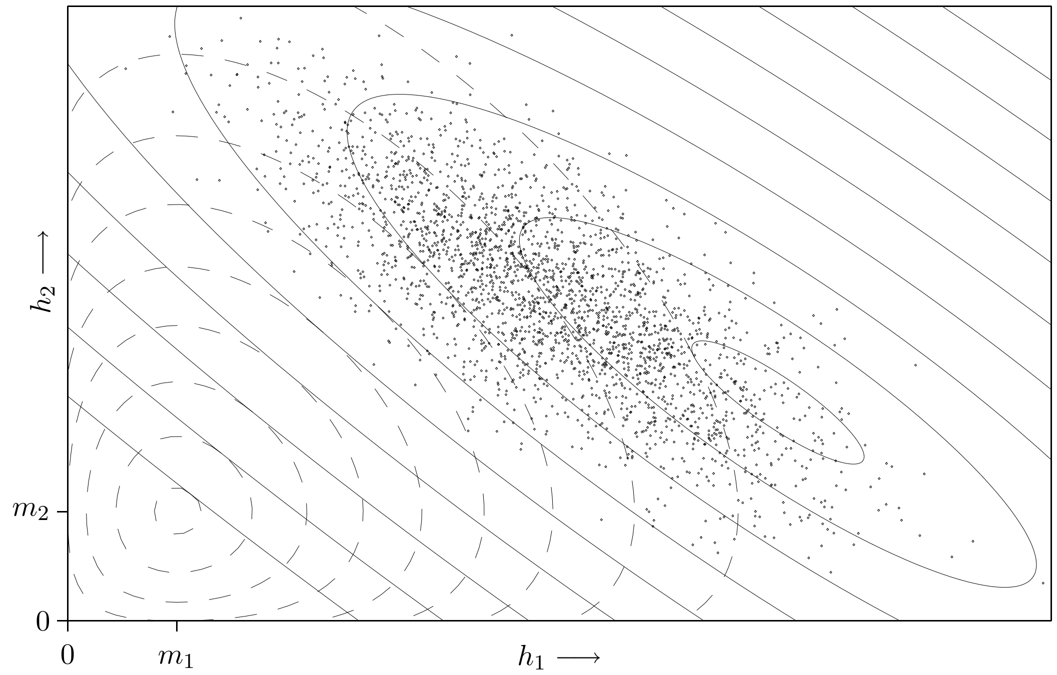

Figure 2.5: Maximum entropy cloud.

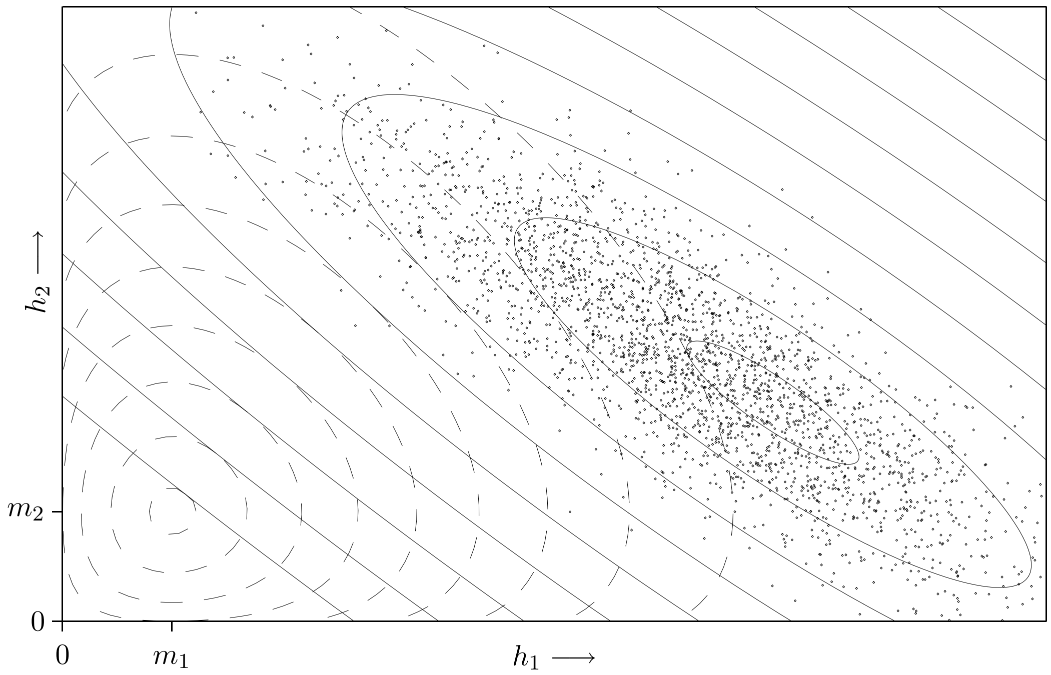

Figure 2.6: Maximum entropy cloud.

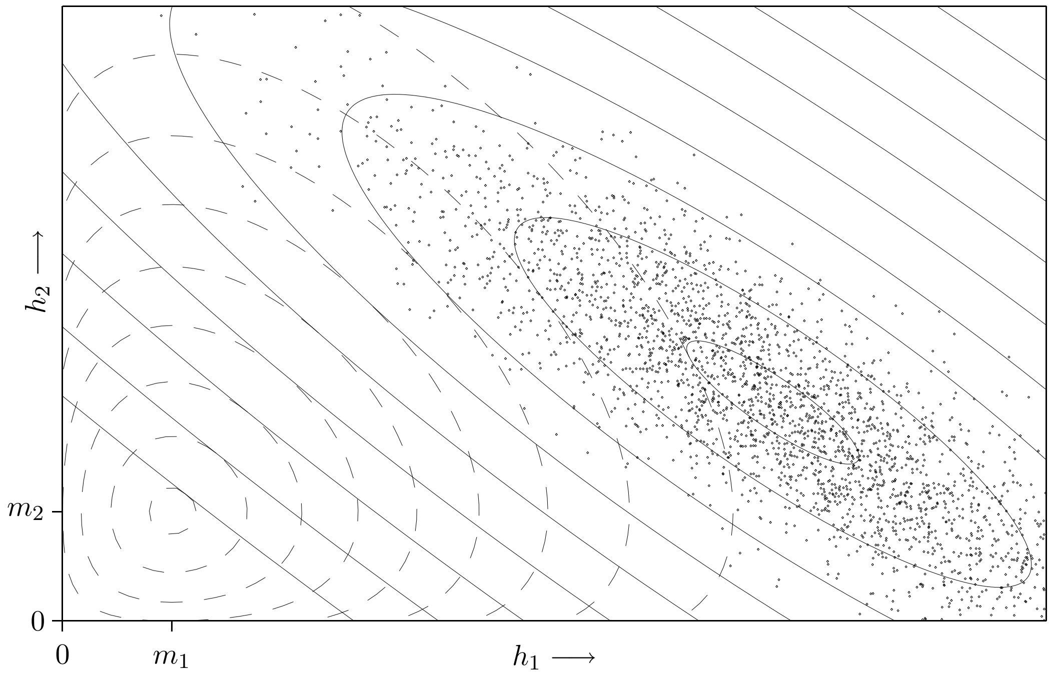

Figure 2.7: Maximum entropy cloud.

Integrated maximum entropy cloud.

The clouds in Figures 2.2 to 2.7 were plotted with fixed numbers of points, in order to produce clear pictures. Had they been plotted in accordance with the posterior probability of their α values, the initial clouds far from the "best" Figure 2.6 would have been invisibly faint.

Figure 2.8 shows the full posterior cloud, correctly integrated over the permissible values of α. Even for this very small-scale two-cell problem, the integrated cloud is visually indistinguishable from the "best" individual cloud (Figure 2.6) obtained by fixing α at its most probable value.

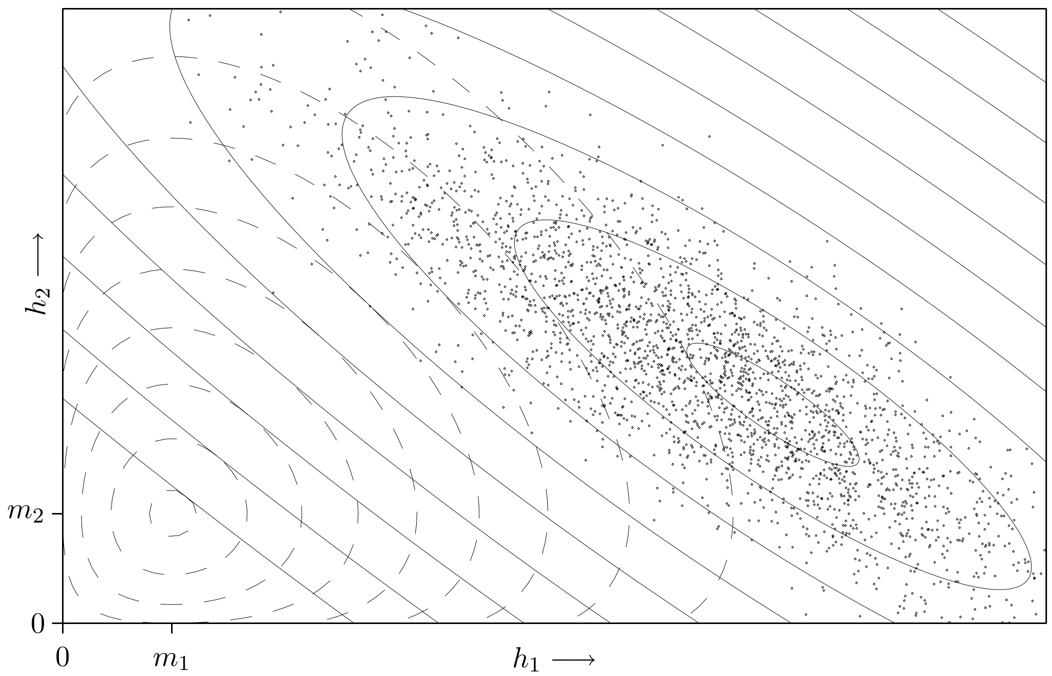

Figure 2.8: Integrated maximum entropy cloud.

2.2 Inferences about the reconstruction

The posterior probability cloud $\Pr(\boldsymbol{h} \mid \boldsymbol{D})$ has the interesting and entirely correct property that as the number of cells $L \to \infty$ the quantity h_i in any particular cell goes to zero as $O(L^{-1})$, whereas the corresponding standard deviation goes to zero only as $O(L^{-1/2})$. This means that the proportional error on individual components of $\boldsymbol{h}$ becomes arbitrarily large in the continuum limit. This is correct, because one should not expect macroscopic data of integral form

$$D_k = \int dx\, h(x)\, R_k(x) \pm \text{noise}$$

to be informative about the microscopic structure of $\boldsymbol{h}$. However, macroscopic data *are* informative about *macro*scopic structure, and integrals of the form

$$\rho = \int dx\, h(x)\, p(x)$$

where p is a "mask" function of position *are* well determined. Technically, we may note at this point that the positive additive distribution h really *is* a distribution, and not a function. It is defined through data integrals over position and used through other integrals over position, so that it is a member of the dual vector space to that of functions of position. Members of this space are formally known as distributions, and their pointwise values become undefined in the continuum limit.

Digitising to

$$\rho = \sum_{i=1}^{L} h_i p_i$$

the probability cloud for $\boldsymbol{h}$ integrates to give a Gaussian posterior density $\Pr(\rho \mid \boldsymbol{D})$ for ρ, with

$$\rho = \widehat{\rho} \pm \delta\rho$$

where

$$\widehat{\rho} = \sum \widehat{h}_i p_i = \widehat{\boldsymbol{h}}^T \boldsymbol{p}$$

(as might be expected) and

$$(\delta\rho)^2 = \boldsymbol{p}^T [\boldsymbol{\mu}^{1/2}]\, \boldsymbol{B}^{-1} [\boldsymbol{\mu}^{1/2}] \boldsymbol{p} / \widehat{\alpha}.$$

For a given mask $p(x)$, this posterior density of ρ is well defined in the continuum limit $L \to \infty$. $p(x)$ might, for example, be set to 1 in a certain domain and 0 elsewhere, in which case ρ would estimate the amount of h in that domain. To investigate a difference between two regions, ρ might be set $+1$ in one region, -1 in the other, and 0 elsewhere. Clearly there are many possibilities.

Note that the probability density of ρ, like that of $\boldsymbol{h}$, is conditional in particular on the assignment of the default model $\boldsymbol{m}$. Were p to be one of the experimental response functions R_k, the corresponding mock datum would not reproduce the actual datum D_k exactly: prior probability factors almost always destroy exact fits to data. Neither would the error σ_k be reproduced exactly. In fact the estimated error would be less than σ_k, possibly considerably less.

Indeed, if the original model $\boldsymbol{m}$ was sufficiently close to the data that there was no evidence for any difference, the entropy formalism would return $\boldsymbol{h} = \boldsymbol{m}$, and would claim all estimates to be perfectly accurate with zero error. That does not mean that the estimates become magically accurate—it just means that there is no evidence for any deviation from the model. Presumably the user is somewhat uncertain about what the reconstruction should be, otherwise there would be little reason to run maximum entropy. That means that the user will also be uncertain about the model $\boldsymbol{m}$, because otherwise he would simply assign "$\boldsymbol{m}$ = the assumed truth". If this assignment of $\boldsymbol{m}$ was correct, the data ought to agree reasonably well, so that the formalism would be entirely correct to return $\boldsymbol{h} = \boldsymbol{m}$ with no error, thus ignoring such discrepancies as could plausibly be explained as noise.

Logically, such uncertainties in the model should always be taken into account, and will induce their own variations in subsidiary estimates of derived quantities. In the special case of the entropy formalism returning $\alpha = \infty$ and $\boldsymbol{h} = \boldsymbol{m}$, the entire onus of such variations falls upon the model. Indeed, advanced use of maximum entropy relies on suitably sophisticated treatment of the model $\boldsymbol{m}$ (Gull 1989). In practice, though, assignment of a simple, uniform model often gives good reconstructions with sensible error bars.

One warning is that the statistics of ρ are derived from a Gaussian approximation to $\Pr(\boldsymbol{h} \mid \boldsymbol{D})$. It can happen that a computed range extends into negative values, (e.g., $\widehat{\rho} \pm \delta\rho = 0.1 \pm 0.3$) even when ρ is a positive functional of the positive distribution $\boldsymbol{h}$. Such results signal a local breakdown of the Gaussian approximation, which we are presently unable to quantify.

Although the above discussion concerned inference about a *linear* functional ρ of the distribution h, this restriction is by no means necessary. *Any* functional $\rho = \phi(h)$ has a probability density

$$\Pr(\rho \mid D) = \int \delta(\rho - \phi(h)) \Pr(h \mid D)\, d^L h / \det[\boldsymbol{\mu}^{1/2}]$$

derived from that of h itself.

The median location in a one-dimensional distribution is a good example of a nonlinear functional. The location of a maximum is another example. Here, though, one has to be more careful, because pointwise values of a distribution become undefined in the continuum limit, and the location of the maximum becomes correspondingly uncertain. That means that one should blur h to some finite resolution before attempting to determine a maximum.

Whatever functional ρ is required, one can obtain independent samples from its distribution simply by taking independent samples h_t ($t = 1, 2, \ldots, T$) from the posterior probability density $\Pr(h \mid \boldsymbol{D})$ of h, and evaluating $\rho(h_t)$ for each. Then the mean $\widehat{\rho}$ is estimated as

$$\widehat{\rho} = T^{-1} \sum_t \rho(h_t),$$

the variance as

$$(\delta\rho)^2 = T^{-1} \sum_t (\rho(h_t) - \widehat{\rho})^2$$

and so on. Other facets of the (non-Gaussian) probability density of ρ can also be estimated, provided one takes sufficient samples.

A particularly useful form of presentation is to display the samples h_t as a "movie" with t representing time. This is even more effective if the successive samples are allowed to be correlated, because one then sees a sample h_t moving ergodically and quasi-continuously through its probability distribution. The reliability of features in h can easily be assessed by eye from movie displays: all one does is watch the feature to see how frequently it is present.

All this extra generality, to nonlinear functionals and movie displays, relies on being able to compute samples from the posterior probability density of h. It is one of the strengths of `MemSys5` that this is now possible.

2.3 Basic iterative algorithm

The most efficient way of computing the required central reconstruction $\widehat{\boldsymbol{h}}(\widehat{\alpha})$ appears to be to iterate down the maximum entropy trajectory, decreasing α from its initial value of ∞ where $\boldsymbol{h} = \boldsymbol{m}$ until the stopping value is reached.

Having selected a value of α, the first need is for a procedure to iterate $\boldsymbol{h}$ towards $\widehat{\boldsymbol{h}}(\alpha)$ at that value. This is defined by the maximum of

$$Q(\boldsymbol{h}) = \alpha \mathcal{S}(\boldsymbol{h}) - \mathcal{L}(\boldsymbol{h})$$

Locally, the quadratic approximation to Q is

$$Q(\boldsymbol{h} + \delta\boldsymbol{h}) = Q(\boldsymbol{h}) + \delta\boldsymbol{h}^T \nabla Q + \frac{1}{2} \delta\boldsymbol{h}^T\, \nabla\nabla Q\, \delta\boldsymbol{h},$$

where

$$\nabla\nabla Q = -[\boldsymbol{\mu}^{-1/2}]\ (\alpha \boldsymbol{I} + \boldsymbol{A})\ [\boldsymbol{\mu}^{-1/2}]$$

and this may be presumed to be sufficiently accurate within some trust region near $\boldsymbol{h}$, defined using the entropy metric $[\boldsymbol{\mu}]$ by

$$|\delta \boldsymbol{r}|^2 = \delta \boldsymbol{h}^T [\boldsymbol{\mu}]^{-1}\, \delta \boldsymbol{h} \le r_0^2.$$

The distance limit r_0 defining the trust region radius should, on dimensional grounds, be set to some value of the order of $(\text{trace}[\boldsymbol{\mu}])^{1/2}$. Maximisation of Q within the trust region is effected by

$$\delta \boldsymbol{h} = [\boldsymbol{\mu}^{1/2}]\, (\beta \boldsymbol{I} + \boldsymbol{A})^{-1}\, [\boldsymbol{\mu}^{1/2}]\, \nabla Q$$

where $\beta \ge \alpha$ is set just large enough to ensure that the trust region constraint is obeyed.

The second need is for α to be changed (usually downwards) towards the stopping value. For safety, this should be done in steps sufficiently small that the corresponding change in $\boldsymbol{h}$ should not violate the current trust region constraint. This can be done alongside the iteration of $\boldsymbol{h}$ itself.

The third need is to converge to the correct stopping value $\widehat{\alpha}$. This can always be defined by an expression of the form

$$\Omega(\alpha) = 1 \qquad \text{with} \qquad \frac{d\Omega}{d\alpha} < 0.$$

For classic maximum entropy, $\Omega = G/(-2\alpha\mathcal{S})$, although other criteria can be defined. Derivative information about $d\Omega/d\alpha$ is generally difficult to compute, so that the stopping value is best approached by extrapolating or interpolating a table of values of (α, Ω) remembered from previous iterates.

Finally, a **termination criterion** is needed to determine when the computed probability cloud is sufficiently close to correct that the algorithm should be stopped. The correct cloud, centred at the correct trajectory point $\widehat{\boldsymbol{h}}$, is given by (2.4) on page 19:

$$\text{Pr}_0(\boldsymbol{h} \mid \boldsymbol{D}) = \text{constant} \times \exp\left(-\frac{\alpha}{2}(\boldsymbol{h} - \widehat{\boldsymbol{h}})^T [\boldsymbol{\mu}^{-1/2}]\, \boldsymbol{B}\, [\boldsymbol{\mu}^{-1/2}]\, (\boldsymbol{h} - \widehat{\boldsymbol{h}})\right).$$

If a slightly different estimate $\tilde{\boldsymbol{h}}$ were produced, it would be taken to represent the slightly different cloud

$$\text{Pr}_1(\boldsymbol{h} \mid \boldsymbol{D}) = \text{constant} \times \exp\left(-\frac{\alpha}{2}(\boldsymbol{h} - \tilde{\boldsymbol{h}})^T [\boldsymbol{\mu}^{-1/2}]\, \boldsymbol{B}\, [\boldsymbol{\mu}^{-1/2}]\, (\boldsymbol{h} - \tilde{\boldsymbol{h}})\right).$$

The mismatch between these clouds should be measured by their (positive) cross-entropy

$$H = \int \text{Pr}_1 \log(\text{Pr}_1 \,/\, \text{Pr}_0)\, d^L h / (\det[\boldsymbol{\mu}])^{1/2}$$

which evaluates to

$$\begin{aligned} H &= \frac{\alpha}{2}(\tilde{\boldsymbol{h}} - \widehat{\boldsymbol{h}})^T [\boldsymbol{\mu}^{-1/2}]\, \boldsymbol{B}\, [\boldsymbol{\mu}^{-1/2}]\, (\tilde{\boldsymbol{h}} - \widehat{\boldsymbol{h}}) \\ &= \frac{1}{2\alpha} \boldsymbol{g}^T [\boldsymbol{\mu}^{1/2}]\, \boldsymbol{B}^{-1} [\boldsymbol{\mu}^{1/2}]\, \boldsymbol{g} \end{aligned}$$

where

$$\begin{aligned} \boldsymbol{g} &= \nabla Q \\ &= \alpha \nabla \mathcal{S} + \left(\frac{\partial \boldsymbol{F}}{\partial \boldsymbol{h}}\right)^T [\boldsymbol{\sigma}^{-2}]\, (\boldsymbol{D} - \boldsymbol{F}) \end{aligned}$$

for Gaussian errors. Because of noise on the data, we expect random fluctuations $\pm\sigma$ on each datum, so that our knowledge of $\boldsymbol{g}$ is limited by

$$\delta \boldsymbol{g} = \left(\frac{\partial \boldsymbol{F}}{\partial \boldsymbol{h}}\right)^T [\boldsymbol{\sigma}^{-1}]\, \boldsymbol{r}$$

where $\boldsymbol{r}$ is a random vector of unit normal components. Accordingly, the expectation mismatch between clouds due to noise is

$$\begin{aligned}\langle H\rangle &= \frac{1}{2\alpha}\left\langle \boldsymbol{r}^T[\boldsymbol{\sigma}^{-1}]\left(\frac{\partial \boldsymbol{F}}{\partial \boldsymbol{h}}\right)[\boldsymbol{\mu}^{1/2}]\,\boldsymbol{B}^{-1}[\boldsymbol{\mu}^{1/2}]\left(\frac{\partial \boldsymbol{F}}{\partial \boldsymbol{h}}\right)^T[\boldsymbol{\sigma}^{-1}]\,\boldsymbol{r}\right\rangle \\ &= \frac{1}{2}\operatorname{trace}((\alpha\boldsymbol{B})^{-1}\boldsymbol{A}) \\ &= G/2.\end{aligned}$$

This result can be understood by noticing that "bad" degrees of freedom in $\boldsymbol{h}$ are controlled by the entropy, and do not fluctuate with the noise, so only the good degrees of freedom contribute to the mismatch.

We see that if H is less than some fraction of G, then the cloud represented by the current estimate $\tilde{\boldsymbol{h}}$ overlaps the true cloud to within the corresponding fraction of the noise. This mathematical guarantee is the termination criterion.

The basic iterative algorithm can be summarised as follows:

(1) Scalars:

$$\mathcal{S}(\boldsymbol{h}) = \sum(\boldsymbol{h} - \boldsymbol{m} - \boldsymbol{h}\log(\boldsymbol{h}/\boldsymbol{m}))$$

and

$$\mathcal{L}(\boldsymbol{h}) = \frac{1}{2}(\boldsymbol{D} - \boldsymbol{F}(\boldsymbol{h}))^T[\boldsymbol{\sigma}^{-2}]\,(\boldsymbol{D} - \boldsymbol{F}(\boldsymbol{h})).$$

(2) Iteration:

$$\delta\boldsymbol{h} = [\boldsymbol{\mu}^{1/2}]\,(\beta\boldsymbol{I} + \boldsymbol{A})^{-1}[\boldsymbol{\mu}^{1/2}]\,\boldsymbol{g}$$

and

$$H = \frac{1}{2\alpha}\boldsymbol{g}^T[\boldsymbol{\mu}^{1/2}]\,\boldsymbol{B}^{-1}[\boldsymbol{\mu}^{1/2}]\,\boldsymbol{g}$$

with

$$|\delta\boldsymbol{r}|^2 = \delta\boldsymbol{h}^T[\boldsymbol{\mu}^{-1}]\,\delta\boldsymbol{h} \le r_0^2$$

where

$$\boldsymbol{g} = \alpha\nabla\mathcal{S} - \nabla\mathcal{L}.$$

(3) Probabilities:

$$\Pr(\boldsymbol{D} \mid \alpha) = Z_{\mathcal{L}}^{-1}\,\exp(\alpha\mathcal{S}(\widehat{\boldsymbol{h}}) - \mathcal{L}(\widehat{\boldsymbol{h}}))\,(\det\boldsymbol{B})^{-1/2}$$

and

$$G = \operatorname{trace}((\alpha\boldsymbol{B})^{-1}\boldsymbol{A})$$

where

$$\boldsymbol{A} = [\boldsymbol{\mu}^{1/2}]\,\nabla\nabla\mathcal{L}\,[\boldsymbol{\mu}^{1/2}]$$

and

$$\boldsymbol{B} = \boldsymbol{I} + \boldsymbol{A}/\alpha.$$

G gives the stopping criterion, and $\Pr(\boldsymbol{D} \mid \widehat{\alpha})$ enables variables to be inferred.

(4) Samples from the posterior cloud $\Pr(\boldsymbol{h} \mid \boldsymbol{D})$

$$\boldsymbol{h} = \widehat{\boldsymbol{h}} + [\boldsymbol{\mu}^{1/2}]\,\boldsymbol{B}^{-1/2}\boldsymbol{r}/\alpha^{1/2}.$$

(5) Linear features of $\boldsymbol{h}$:

$$\widehat{\rho} = \sum \widehat{h}_i p_i = \boldsymbol{h}^T\boldsymbol{p}$$

and

$$(\delta\rho)^2 = \boldsymbol{p}^T[\boldsymbol{\mu}^{1/2}]\,\boldsymbol{B}^{-1}[\boldsymbol{\mu}^{1/2}]\,\boldsymbol{p}/\alpha.$$

2.4 The intrinsic correlation function

One of the fundamental axioms on which maximum entropy is based (Skilling 1989) is that entropy maximisation should not itself introduce correlations between individual elements of a PAD. This was illustrated in the "kangaroo argument" of Section 1.2. Nonetheless, in many (if not all) cases the object of interest is known *a priori* to contain correlations, typically between neighbouring elements. The source and scale of the correlations depends on the physical nature of the object or phenomenon under investigation.

The recommended technique for encoding this correlation is to derive the quantity of interest, such as an image or spectrum, and henceforth designated $f(y)$ or $\boldsymbol{f}$ in the discrete case with M cells, as a 'blurred' version of an underlying 'hidden variables' PAD $h(x)$ (Gull 1989, Charter 1990). The 'blurring' is accomplished with an operator $C(y, x)$, through an integral of the form

$$f(y) = \int C(y,x)\, h(x)\, dx$$

or, in the discrete case

$$f_j = \sum_{i=1}^{L} C_{ji} h_i \qquad (j = 1,\, 2,\, \ldots,\, M)$$

so that

$$\boldsymbol{f} = \boldsymbol{C}\, \boldsymbol{h}.$$

This operator is known as the **intrinsic correlation function** (ICF).

By construction, all the prior expectation of correlation is assigned to the ICF, so that h itself is *a priori* uncorrelated. Accordingly, it is legitimate to use an entropic prior on h, and all the corresponding analysis remains valid. Without the ICF construction, the MaxEnt analysis would simply fall apart. Often, the intrinsic correlation may be modelled by convolution with a Gaussian (say) of some given width. More sophisticated usage is possible, however, and the explicit incorporation of intrinsic correlation functions is a major advance in the maximum entropy formalism.

Depending on the nature of the ICF, one may wish to digitise h and f at different resolution, so that $\boldsymbol{h}$ and $\boldsymbol{f}$ may be of different sizes, and so should formally be considered to belong to two different spaces. The space to which $\boldsymbol{h}$ belongs is known as **hidden space**, and that to which $\boldsymbol{f}$ belongs is known as **visible space**. We let L be the number of cells in hidden space (as before), and set M to the number of cells in visible space.

It was pointed out in Section 2.2 that the proportional error on individual components of $\boldsymbol{h}$ becomes arbitrarily large in the continuum limit. Since $f(y)$ is defined in terms of an integral over $h(x)$, however, pointwise errors on f are well-behaved. It should be noted, though, that a series of pointwise errors on f may well be highly correlated, and should therefore be interpreted with caution.

2.5 Predicted data

Although nonlinear variants are allowable, the predicted data $\boldsymbol{F}$ are generally considered to be related to the visible distribution $\boldsymbol{f}$ via a $N \times M$ matrix $\boldsymbol{R}$ of response functions, so that

$$F_k \;=\; \sum_{j=1}^{M} R_{kj} f_j \qquad (k = 1,\, 2,\, \ldots,\, N)$$

$$= \sum_{j=1}^{M} R_{kj} \sum_{i=1}^{L} C_{ji} h_i$$

and hence

$$\boldsymbol{F} = \boldsymbol{R}\,\boldsymbol{C}\,\boldsymbol{h} \quad \text{and} \quad \left(\frac{\partial \boldsymbol{F}}{\partial \boldsymbol{h}}\right) = \boldsymbol{R}\,\boldsymbol{C}.$$

Note again the dimensions of the three spaces:

$$\begin{aligned} L &= \text{dimension of hidden space} \\ M &= \text{dimension of visible space} \\ N &= \text{dimension of data space.} \end{aligned}$$

2.6 Inferences about the noise level and other variables

The classic maximum entropy analysis carries with it the overall probability of the data from (2.5) on page 19:

$$\begin{aligned} \Pr(\boldsymbol{D}) &= \int d\alpha \Pr(\alpha \mid \boldsymbol{D}) = \Pr(\widehat{\alpha} \mid \boldsymbol{D}) \\ &= (2\pi)^{-N/2} \det[\boldsymbol{\sigma}^{-1}] \exp(\widehat{\alpha}\mathcal{S}(\widehat{\boldsymbol{h}}) - \mathcal{L}(\widehat{\boldsymbol{h}}))\,(\det \boldsymbol{B})^{-1/2}. \end{aligned}$$

This expression becomes useful when it is realised that, like any other probabilistic expression, it is conditional on the underlying assumptions. In fact all the probabilities derived in the classic maximum entropy analysis have been conditional upon a choice of model $\boldsymbol{m}$, experimental variables defining the response functions, noise amplitudes $\boldsymbol{\sigma}$, and so on, so that $\Pr(\boldsymbol{D})$ is really a shorthand for

$$\Pr(\boldsymbol{D} \mid \boldsymbol{m}, \boldsymbol{R}, \boldsymbol{\sigma}, \ldots).$$

If such variables are imperfectly known, these conditional probability values can be used to refine our knowledge of them, by using Bayes' theorem in the form

$$\Pr(\text{variables} \mid \boldsymbol{D}) = \text{constant} \times \Pr(\text{variables}) \Pr(\boldsymbol{D} \mid \text{variables}).$$

Ideally, one would set up a full prior for unknown variables and integrate them out in order to determine $\Pr(\boldsymbol{h} \mid \boldsymbol{D})$. In practice, though, with large datasets it usually suffices to select the single "best" values of the variables, just as was the case for the regularisation constant α.

A common special case of this concerns experimental data in which the overall noise level is uncertain, so that all the standard deviations $\boldsymbol{\sigma}$ in $\Pr(\boldsymbol{D})$ above should be scaled with some coefficient c. Rescaling α to α/c^2 for convenience gives

$$\Pr(\boldsymbol{D} \mid \alpha, c) = (2\pi c^2)^{-N/2} \det[\boldsymbol{\sigma}^{-1}] \exp\Big((\alpha\mathcal{S}(\widehat{\boldsymbol{h}}) - \mathcal{L}(\widehat{\boldsymbol{h}}))/c^2\Big)\;(\det \boldsymbol{B})^{-1/2}.$$

For this case, the maximum entropy trajectory itself is unaltered and parameterised by the same values of α, though the probability clouds for $\boldsymbol{h}$ are of different overall size. The Evidence is maximised over c when

$$c^2 = 2\,(\mathcal{L} - \alpha\mathcal{S})/N.$$

At this scaling, we note that (for linear data) the χ^2 misfit statistic

$$\chi^2 = (\boldsymbol{D} - \boldsymbol{F})^T [c^{-2}\boldsymbol{\sigma}^{-2}]\,(\boldsymbol{D} - \boldsymbol{F})$$

evaluates to

$$\chi^2 = 2\,\mathcal{L}(\boldsymbol{h})/c^2.$$

Thus, when the stopping criterion is satisfied at the most probable α,

$$\chi^2 + G = N \qquad (= \text{number of data}).$$

We see that the N data separate into the "good" measurements which are sufficiently informative to be fitted and induce structure in the central reconstruction $\widehat{\boldsymbol{h}}$, and the remaining number "χ^2" which are not informative about $\boldsymbol{h}$ and which are "bad" measurements serving merely to specify the noise scaling.

A corollary of this is that χ^2 at $\widehat{\boldsymbol{h}}$ is always less than the number of data. Redressing the balance, the average χ^2 over the whole posterior cloud is just N.

With large datasets, the noise scaling is determined to high accuracy, enough to overwhelm all but the most rigid prior knowledge of it, essentially because all the many "bad" measurements are being used to determine this single number. Accordingly, automatic noise scaling is usually recommended.

Chapter 3

Extensions and generalisations

3.1 Positive/negative distributions

The maximum entropy method can be extended to reconstruct distributions $\boldsymbol{h}$ which can be either positive or negative. It may be appropriate to describe such a distribution as the difference between two subsidiary positive distributions $\boldsymbol{u}$ and $\boldsymbol{v}$: thus

$$\boldsymbol{h} = \boldsymbol{u} - \boldsymbol{v}.$$

The total entropy, relative to a common model $\boldsymbol{m}$, is

$$S(\boldsymbol{u}, \boldsymbol{v}) = \sum_{i=1}^{L} (u_i - m_i - u_i \log(u_i/m_i)) + \sum_{i=1}^{L} (v_i - m_i - v_i \log(v_i/m_i)).$$

If we wished to find both $\boldsymbol{u}$ and $\boldsymbol{v}$ separately, we would incorporate the linear mapping $(\boldsymbol{u}, \boldsymbol{v}) \to \boldsymbol{h}$ into the ICF, and proceed with the standard analysis. However, we are not usually interested in this detail, wishing to estimate $\boldsymbol{h}$ only, with its corresponding visible distribution $\boldsymbol{f}$. In that case, we note that under any constraint on $\boldsymbol{h}$, the gradients with respect to $\boldsymbol{u}$ and $\boldsymbol{v}$ must be equal and opposite,

$$\frac{\partial S}{\partial \boldsymbol{u}} = -\frac{\partial S}{\partial \boldsymbol{v}}$$

so that the maximising u and v always obey

$$uv = m^2.$$

The entropy can now be rewritten as a functional of $\boldsymbol{h}$ alone.

$$S(\boldsymbol{h}) = \sum_{i=1}^{L} (\psi_i - 2m_i - h_i \log((\psi_i + h_i)/2m_i)), \qquad \psi_i = (h_i^2 + 4m_i^2)^{1/2}.$$

All the original maximum entropy algebra can be performed with this revised form, with appropriate modifications to the gradient and curvature. The underlying PADs $\boldsymbol{u}$ and $\boldsymbol{v}$ disappear from the subsequent formulae, and we can work directly with $\boldsymbol{h}$, which may now be of either sign.

3.2 Fermi–Dirac distributions

Another extension is to positive distributions which have an upper limit, such as reflective albedo, or electron density in a semiconductor band. Here we may think of the PAD $\boldsymbol{h}$ being accompanied by a second PAD $\boldsymbol{k}$, such that

$$h_i + k_i = 1 \qquad \text{in each cell.}$$

(Setting the upper limit to 1 is a mere convention; any other value can be scaled to 1 by appropriately re-scaling $\boldsymbol{h}$ and $\boldsymbol{k}$.) Because both $\boldsymbol{h}$ and $\boldsymbol{k}$ are positive, we have

$$0 \leq h_i \leq 1 \qquad \text{in each cell.}$$

The total entropy, relative to a model m_i for h_i (and $1 - m_i$ for k_i) is

$$S(\boldsymbol{h}) = \sum_{i=1}^{L} \left(-h_i \log \frac{h_i}{m_i} - (1 - h_i) \log \frac{1 - h_i}{1 - m_i} \right)$$

Again, all the original maximum entropy algebra can be performed with this revised form, with appropriate modifications. The extra PAD $\boldsymbol{k}$ disappears from the subsequent formulae, and we can work directly with the now-limited $\boldsymbol{h}$.

3.3 Quadratic entropy

Sometimes, one desires neither an upper nor a lower limit on the values of h_i. In that case we define

$$S(\boldsymbol{h}) = -\sum_{i=1}^{L} h_i^2 / 2m_i.$$

Maximum entropy then reduces to least squares, scaled proportionally to model variance m. The simplest "derivation" of this assignment is to note that in the large-α limit $\boldsymbol{h} \simeq \boldsymbol{m}$, the standard entropy approaches

$$S(\boldsymbol{h}) \simeq -\sum_{i=1}^{L} (h_i - m_i)^2 / 2m_i,$$

whence an offset of the origin of h gives the assigned quadratic form.

3.4 Fixed total

One does not always require the full generality of the standard entropy formula, and it is possible to restrict this in various ways. One such restriction is to require a fixed total

$$\sum_{i=1}^{L} h_i = \sum_{i=1}^{L} m_i = \text{fixed constant.}$$

Either one may know this total $\sum h$ accurately, from a particularly precise measurement, or one may impose it as part of the structure of the problem, as when $\boldsymbol{h}$ represents a set of proportions, summing to one by definition. This restriction on $\boldsymbol{h}$ removes one degree of freedom from the possible inferences. The calculations are performed as usual, but with that degree of freedom explicitly projected out.

Chapter 4

Algorithmic details

4.1 Iterative algorithm in hidden space

With the various extensions and generalisations just described, the stopping criterion Ω takes one of the following forms:

Option 1:	Classic maximum entropy:	$\Omega = G/(-2\alpha\mathcal{S})$
Option 2:	Classic automatic, with noise scaling:	$\Omega = Gc^2/(-2\alpha\mathcal{S})$ with $c^2 = 2\,(\mathcal{L} - \alpha\mathcal{S})/N$
Option 3:	*Ad hoc* "α fixed":	$\Omega = 1/\alpha$
Option 4:	Historic maximum entropy :	$\Omega = N/(2\mathcal{L})$ (see Appendix B)

The iterative hidden-space algorithm can then be summarised as follows:

(1) Scalars:

$$\mathcal{S}(\boldsymbol{h}) = \sum(\boldsymbol{h} - \boldsymbol{m} - \boldsymbol{h}\log(\boldsymbol{h}/\boldsymbol{m})) \qquad \text{or variant}$$

and

$$\mathcal{L}(\boldsymbol{h}) = \frac{1}{2}(\boldsymbol{D} - \boldsymbol{R}\,\boldsymbol{C}\,\boldsymbol{h})^T[\boldsymbol{\sigma}^{-2}]\,(\boldsymbol{D} - \boldsymbol{R}\,\boldsymbol{C}\,\boldsymbol{h}).$$

(2) Iteration:

$$\delta\boldsymbol{h} = [\boldsymbol{\mu}^{1/2}]\,(\beta\boldsymbol{I} + \boldsymbol{A})^{-1}[\boldsymbol{\mu}^{1/2}]\,\boldsymbol{g}$$

and

$$H = \frac{1}{2\alpha}\boldsymbol{g}^T[\boldsymbol{\mu}^{1/2}]\,\boldsymbol{B}^{-1}[\boldsymbol{\mu}^{1/2}]\,\boldsymbol{g}$$

with

$$|\delta\boldsymbol{r}|^2 = \delta\boldsymbol{h}^T[\boldsymbol{\mu}^{-1}]\,\delta\boldsymbol{h} \le r_0^2$$

where

$$\boldsymbol{g} = \alpha\nabla\mathcal{S} + \boldsymbol{C}^T\boldsymbol{R}^T[\boldsymbol{\sigma}^{-2}]\,(\boldsymbol{D} - \boldsymbol{R}\,\boldsymbol{C}\,\boldsymbol{h}).$$

(3) Probabilities:

$$\Pr(\boldsymbol{D} \mid \alpha, c) = (2\pi c^2)^{-N/2}\,\det[\boldsymbol{\sigma}^{-1}]\,\exp\Big((\alpha\mathcal{S}(\widehat{\boldsymbol{h}}) - \mathcal{L}(\widehat{\boldsymbol{h}}))/c^2\Big)\,(\det\boldsymbol{B})^{-1/2}$$

and

$$G = \mathrm{trace}((\alpha\boldsymbol{B})^{-1}\boldsymbol{A})$$

where

$$\boldsymbol{A} = [\boldsymbol{\mu}^{1/2}]\,\boldsymbol{C}^T\boldsymbol{R}^T[\boldsymbol{\sigma}^{-2}]\,\boldsymbol{R}\,\boldsymbol{C}\,[\boldsymbol{\mu}^{1/2}]$$

and

$$\boldsymbol{B} = \boldsymbol{I} + \boldsymbol{A}/\alpha.$$

For the classic options, G gives the stopping criterion, and $\Pr(\boldsymbol{D})$ enables variables to be inferred.

(4) Samples from the posterior cloud $\Pr(\boldsymbol{h} \mid \boldsymbol{D})$:

$$\boldsymbol{h} = \widehat{\boldsymbol{h}} + \left(\frac{c^2}{\alpha}\right)^{1/2} [\boldsymbol{\mu}^{1/2}]\, \boldsymbol{B}^{-1/2} \boldsymbol{r}.$$

(5) Linear features of $\boldsymbol{h}$:

$$\widehat{\rho} = \sum \widehat{h}_i p_i = \boldsymbol{h}^T \boldsymbol{p}$$

and

$$(\delta\rho)^2 = \frac{c^2}{\alpha}\, \boldsymbol{p}^T [\boldsymbol{\mu}^{1/2}]\, \boldsymbol{B}^{-1} [\boldsymbol{\mu}^{1/2}]\, \boldsymbol{p}.$$

4.2 Vector coding

The matrix operations which are needed in various parts of the algorithm are

$$\boldsymbol{B}^{-1}\boldsymbol{b}, \qquad \boldsymbol{b}^T\boldsymbol{B}^{-1}\boldsymbol{b}, \qquad \boldsymbol{B}^{-1/2}\boldsymbol{b}$$

and

$$\operatorname{trace}(\boldsymbol{B}^{-1}\boldsymbol{A}), \qquad \det(\boldsymbol{B}),$$

where $\boldsymbol{B} = \boldsymbol{I} + \boldsymbol{A}/\alpha$ and $\boldsymbol{A} = [\boldsymbol{\mu}^{1/2}]\, \boldsymbol{C}^T \boldsymbol{R}^T [\boldsymbol{\sigma}^{-2}]\, \boldsymbol{R}\, \boldsymbol{C}\, [\boldsymbol{\mu}^{1/2}]$.

Direct inversion of $\boldsymbol{B}$ and the evaluation of its determinant each require $O(L^3)$ operations. This would be impractical for large datasets, even if the $O(L^2)$ memory locations could be found for the list of elements. A practical large-scale algorithm can store only *vector* quantities, and its use of the transform routines $\boldsymbol{C}$, $\boldsymbol{C}^T$, $\boldsymbol{R}$ and $\boldsymbol{R}^T$ must be restricted to applying them (perhaps several times) to vectors.

In order to evaluate $\boldsymbol{B}^{-1}\boldsymbol{b}$, the obvious, indeed only, vector operand is $\boldsymbol{b}$ itself, so that one becomes restricted to some fairly small n-dimensional subspace spanned by $\boldsymbol{b}$, $\boldsymbol{A}\,\boldsymbol{b}$, $\boldsymbol{A}^2\boldsymbol{b}, \ldots, \boldsymbol{A}^{n-1}\boldsymbol{b}$. Within this subspace, a vector $\boldsymbol{y}_n$ can be found which maximises

$$Y = 2\, \boldsymbol{y}_n^T \boldsymbol{b} - \boldsymbol{y}_n^T \boldsymbol{B}\, \boldsymbol{y}_n.$$

As n increases, the subspace expands and $\boldsymbol{y}$ becomes less constrained, so that Y increases monotonically. Eventually, when n reaches L or perhaps before, Y reaches its greatest possible value

$$Y_0 \le Y_1 \le Y_2 \le \cdots \le Y_{\max} = \boldsymbol{b}^T\boldsymbol{B}^{-1}\boldsymbol{b} \quad \text{at} \quad \boldsymbol{y} = \boldsymbol{B}^{-1}\boldsymbol{b}.$$

By construction, the lower limits on $Y_{\max}$ are optimal in terms of the information available in the given subspace. Although the conjugate gradient algorithm organises this calculation efficiently, we need to know when to stop it.

For this, we introduce another vector $\boldsymbol{z}_n$ in the same subspace which maximises

$$Z = 2\, \boldsymbol{z}^T \boldsymbol{A}\, \boldsymbol{b} - \boldsymbol{z}^T \boldsymbol{B}\, \boldsymbol{A}\, \boldsymbol{z}.$$

This calculation too can be organised by conjugate gradients, and we reach another sequence of optimal lower limits

$$Z_0 \le Z_1 \le Z_2 \le \cdots \le Z_{\max} = \boldsymbol{b}^T\boldsymbol{B}^{-1}\boldsymbol{A}\, \boldsymbol{b}$$

Now

$$Y_{\max} + Z_{\max}/\alpha = \boldsymbol{b}^T \boldsymbol{B}^{-1}(\boldsymbol{I} + \boldsymbol{A}/\alpha)\, \boldsymbol{b} = \boldsymbol{b}^T \boldsymbol{b},$$

which is known on entering the calculations. Hence, just as $Y_0, Y_1, Y_2, \ldots$ give successively improved optimal *lower* limits to $Y_{\max}$, so do Z_0, Z_1, Z_2, ... lead to successively improved optimal *upper* limits. At any stage, we can write

$$\boldsymbol{b}^T \boldsymbol{B}^{-1} \boldsymbol{b} \in [Y_-, Y_+]$$

where

$$Y_- = Y_n, \qquad Y_+ = \boldsymbol{b}^T \boldsymbol{b} - Z_n/\alpha.$$

We terminate the conjugate gradient calculations when n is large enough that the numerical uncertainty is sufficiently small:

$$Y_+ - Y_- \le \epsilon Y_-$$

where ϵ is whatever tolerance (say 0.01) which may be desired.

As a technicality, there is no need to perform the calculations of Y and Z independently. Both use the same base vectors, and they can be merged together. In fact, accuracy is best preserved by slaving both of them to an underlying "master" conjugate gradient maximisation of

$$X = 2\, \boldsymbol{x}^T \boldsymbol{b} - \boldsymbol{x}^T \boldsymbol{A}\, \boldsymbol{x}.$$

$\boldsymbol{B}^{-1/2}$ can be obtained similarly.

The trace and determinant calculations can be carried out with the aid of one or more random normal vectors $\boldsymbol{r}$, whose L components are correlated according to

$$\langle \boldsymbol{r}\, \boldsymbol{r}^T \rangle = \boldsymbol{I}.$$

Consider

$$Z = \boldsymbol{r}^T \boldsymbol{B}^{-1} \boldsymbol{A}\, \boldsymbol{r}.$$

This can be evaluated as above, using $\boldsymbol{r}$ as the input vector and obtaining

$$Z \in [Z_-, Z_+]$$

where

$$Z_- = Z_n, \qquad Z_+ = \alpha(\boldsymbol{r}^T \boldsymbol{r} - Y_n).$$

The expectation over $\boldsymbol{r}$ is

$$\langle Z \rangle = \langle\, \boldsymbol{r}^T \boldsymbol{B}^{-1} \boldsymbol{A}\, \boldsymbol{r}\, \rangle = \mathrm{trace}(\boldsymbol{B}^{-1} \boldsymbol{A}),$$

which is one of the quantities (αG) that we need. Moreover, the variance of this estimate is also available

$$\begin{aligned} (\mathrm{dev}(Z))^2 &= 2\, \mathrm{trace}(\boldsymbol{B}^{-2} \boldsymbol{A}^2) \\ &\approx 2\, \boldsymbol{r}^T \boldsymbol{B}^{-2} \boldsymbol{A}^2\, \boldsymbol{r} \quad \approx \quad 2\, \boldsymbol{z}_n^T \boldsymbol{A}^2\, \boldsymbol{z}_n. \end{aligned}$$

Thus we can write

$$\alpha G = [Z_-, Z_+] \pm \mathrm{dev}(Z).$$

The random variation is additional to the numerical uncertainty caused by terminating the conjugate gradient calculation, and is part of the price we must pay for avoiding the direct, expensive calculation of the trace.

Although the error thus induced depends on the random vector $\boldsymbol{r}$, it will be systematic if the same $\boldsymbol{r}$ is used repeatedly. Thus, provided the same vector is re-used each iterate, it will not affect the actual convergence of the maximum entropy calculation, though it will affect the precise position on the trajectory to which the calculation converges.

This may well not matter much. G is the dimensionless number of good measurements, and the standard deviation of its estimate turns out to be only about (in fact, less than) $G^{1/2}$. For large datasets, with many good measurements, this variation will be relatively small, and unimportant. In effect, the integrations over the complete cloud which are needed to find G are being performed by a Monte Carlo exploration, but the space is so large that, paradoxically, a single exploration is usually sufficient. In any case, the variation can always be reduced, in proportion to the square root, by averaging the results over several random vectors $\boldsymbol{r}$.

An extension of this procedure gives the determinant $\det(\boldsymbol{B})$ in parallel with the evaluation of the trace. The determinant too has its numerical and random uncertainties, so that the evidence too is computed in the form

$$\text{Evidence} = [P_-, P_+] \pm \text{dev}(P).$$

4.3 Data-space algorithm

The hidden-space algorithm outlined in Section 4.1 involves computations with L-dimensional vectors and matrices, ideally with L large to give high resolution. However, any central reconstruction $\widehat{\boldsymbol{h}}$ maximises $Q = \alpha\mathcal{S} - \mathcal{L}$ so that $\nabla Q = \boldsymbol{0}$. Hence for standard entropy $\widehat{\boldsymbol{h}}$ must be of the form (exponentiating componentwise)

$$\widehat{\boldsymbol{h}} = \boldsymbol{m} \exp(\boldsymbol{C}^T \boldsymbol{R}^T [\boldsymbol{\sigma}^{-1}]\, \boldsymbol{w})$$

for some N-dimensional $\boldsymbol{w}$, actually equal to $\alpha^{-1}[\boldsymbol{\sigma}^{-1}]\,(\boldsymbol{D} - \boldsymbol{R}\,\boldsymbol{C}\,\boldsymbol{h})$. Similar expressions can be found for the other variants. By using $\boldsymbol{w}$ to define $\widehat{\boldsymbol{h}}$, memory space is saved when computing at high resolution, and accuracy improves because $\widehat{\boldsymbol{h}}$ automatically lies within the N-dimensional subspace of possible solutions. The data-space algorithm, finding and using $\boldsymbol{w}$, is obtained by translating the hidden-space steps directly into data space.

(0) Preliminary:

$$\boldsymbol{h} = \boldsymbol{m} \exp(\boldsymbol{C}^T \boldsymbol{R}^T [\boldsymbol{\sigma}^{-1}]\, \boldsymbol{w}) \qquad \text{or variant.}$$

(1) Scalars:

$$\mathcal{S}(\boldsymbol{w}) = \sum (\boldsymbol{h} - \boldsymbol{m} - \boldsymbol{h} \log(\boldsymbol{h}/\boldsymbol{m})) \qquad \text{or variant}$$

and

$$\mathcal{L}(\boldsymbol{w}) = \frac{1}{2}(\boldsymbol{D} - \boldsymbol{R}\,\boldsymbol{C}\,\boldsymbol{h})^T [\boldsymbol{\sigma}^{-2}]\,(\boldsymbol{D} - \boldsymbol{R}\,\boldsymbol{C}\,\boldsymbol{h}).$$

(2) Iteration:

$$\delta\boldsymbol{w} = (\beta\boldsymbol{I}' + \boldsymbol{A}')^{-1}\boldsymbol{g}'$$

and

$$H = \frac{1}{2\alpha}\boldsymbol{g}'^T \boldsymbol{B}'^{-1} \boldsymbol{A}'\, \boldsymbol{g}'$$

with

$$|\delta\boldsymbol{r}|^2 = \delta\boldsymbol{w}^T \boldsymbol{A}'\, \delta\boldsymbol{w} \le r_0^2$$

where

$$\boldsymbol{g}' = -\alpha \boldsymbol{w} + [\boldsymbol{\sigma}^{-1}]\,(\boldsymbol{D} - \boldsymbol{R}\,\boldsymbol{C}\,\boldsymbol{h})$$

and

$$\boldsymbol{A}' = [\boldsymbol{\sigma}^{-1}]\,\boldsymbol{R}\,\boldsymbol{C}\,[\boldsymbol{\mu}]\,\boldsymbol{C}^T\boldsymbol{R}^T[\boldsymbol{\sigma}^{-1}]$$

and

$$\boldsymbol{B}' = \boldsymbol{I}' + \boldsymbol{A}'/\alpha.$$

(3) Probabilities:

$$\Pr(\boldsymbol{D} \mid \alpha, c) = (2\pi c^2)^{-N/2}\,\det[\boldsymbol{\sigma}^{-1}]\,\exp\Big((\alpha\mathcal{S}(\widehat{\boldsymbol{h}}) - \mathcal{L}(\widehat{\boldsymbol{h}}))/c^2\Big)\,(\det \boldsymbol{B}')^{-1/2}$$

and

$$G = \mathrm{trace}((\alpha\boldsymbol{B}')^{-1}\boldsymbol{A}').$$

For the classic options, G gives the stopping criterion, and $\Pr(\boldsymbol{D})$ enables variables to be inferred.

(4) Samples from the posterior cloud $\Pr(\boldsymbol{h} \mid \boldsymbol{D})$:

$$\boldsymbol{h} = \widehat{\boldsymbol{h}} + \left(\frac{c^2}{\alpha}\right)^{1/2}[\boldsymbol{\mu}^{1/2}]\,\boldsymbol{B}^{-1/2}\boldsymbol{r}.$$

(5) Linear features of $\boldsymbol{h}$:

$$\widehat{\rho} = \sum \widehat{h}_i p_i = \boldsymbol{h}^T\boldsymbol{p}$$

and

$$(\delta\rho)^2 = \frac{c^2}{\alpha}\,\boldsymbol{p}^T[\boldsymbol{\mu}^{1/2}]\,\boldsymbol{B}^{-1}[\boldsymbol{\mu}^{1/2}]\,\boldsymbol{p}.$$

Part II

Practice

Chapter 5

The MemSys5 software package

The MemSys5 package (mnemonic Maximum entropy method System, 5th generation) of FORTRAN 77 subroutines is designed to converge to the posterior probability cloud $\Pr(\boldsymbol{h} \mid \boldsymbol{D})$, for a wide variety of types of data $\boldsymbol{D}$. Options are available for constant or variable Gaussian noise or Poisson errors, as well as for different stopping criteria.

The purpose of the major subroutine MEM5 is to perform one iterate of the data-space maximum entropy algorithm, initialising a new run if necessary. It updates the regularisation constant α and the Lagrange multipliers $\boldsymbol{w}$, finds the corresponding central reconstruction $\widehat{\boldsymbol{h}} = \boldsymbol{m} \exp(\boldsymbol{C}^T \boldsymbol{R}^T [\boldsymbol{\sigma}^{-1}] \boldsymbol{w})$ or the appropriate variant, and optionally calculates the overall evidence $\Pr(\boldsymbol{D} \mid \alpha)$ and the number of good measurements. It also controls progress down the maximum entropy trajectory towards the desired stopping value.

The purpose of subroutine MOVIE5 is to obtain samples from the posterior probability cloud in hidden space, optionally correlated with previous samples. From this, the user can display a movie in which successive frames show a sample reconstruction moving ergodically through the posterior density (Skilling, Robinson and Gull 1991). This important addition to the MemSys package allows the user to see whatever variability is present in the results. It also allows quantification of nonlinear functionals such as a maximum or a median (Charter 1991), simply by accumulating the values of such quantities over several independent frames.

The purpose of the quantification subroutine MASK5 is to take a user-defined mask $\boldsymbol{p}$ and evaluate $\rho = \boldsymbol{h}^T \boldsymbol{p}$, returning its mean $\widehat{\rho} = \widehat{\boldsymbol{h}}^T \boldsymbol{p}$ and its standard deviation $\delta\rho$. In this way linear features ρ can be estimated without having to average over MOVIE5 samples.

The hidden distribution $\boldsymbol{h}$, visible distribution $\boldsymbol{f}$, data $\boldsymbol{D}$, multipliers $\boldsymbol{w}$, and related quantities used in the algorithm, are stored in **areas** whose contents are manipulated by the MemSys5 package. Henceforward in this document, angle brackets $\langle\ \rangle$ denote an area. MemSys5 itself uses at most 14 areas, but up to 40 can be defined, to allow for extensions and development within the MemSys framework.

The internal arithmetic is carried out to REAL precision throughout.

There is, of course, more detail to be understood, but the basic way of using the system is as follows.

Call `MEINIT` to initialise the system.

Set up storage areas and data $\boldsymbol{D}$.

Optionally `CALL MEMTRQ` to check consistency of transforms.

Repeat ...
```
    CALL MEM5
```
... until converged to $\widehat{\boldsymbol{h}}$, with $\widehat{\boldsymbol{f}} = \boldsymbol{C}\,\widehat{\boldsymbol{h}}$.

Repeat ...
```
    CALL MOVIE5
```
... to generate ergodic samples $\boldsymbol{h}$ from $\Pr(\boldsymbol{h} \mid \boldsymbol{D})$.

Repeat ...
```
    CALL MASK5
```
... to quantify any desired features $\rho = \boldsymbol{h}^T \boldsymbol{p}$.

Chapter 6

Storage areas

6.1 Area structure

The package works with up to 40 areas, each of which may be held in memory or externally on disc or elsewhere at the user's discretion. By convention, areas ⟨`1`⟩ to ⟨`10`⟩ are hidden-space areas, each capable of holding a complete hidden distribution of L cells. Similarly, areas ⟨`11`⟩ to ⟨`20`⟩ are visible-space areas, each capable of holding a complete visible distribution of M cells. Areas ⟨`21`⟩ to ⟨`30`⟩ are data-space areas, each capable of holding a complete set of N data. The uses of the areas are summarised in Table 6.1. Any unused area is available for the user's own purposes, if required.

The `MemSys5` package uses the working space `ST` to perform vector operations on the areas. This working space `ST` is supplied by the user as an argument to the main routines of the package. The package requests access to an area by calling the user-supplied subroutine `VFETCH` before the operation, and relinquishes access to it by calling the user-supplied subroutine `VSTORE` when the operation is complete. There are no restrictions on the storage device or location in which the user holds an area when it is not being accessed by the package, and the address in `ST` at which it is made available to the package may be different on each occasion it is requested. The user can set the number of elements of an area supplied to the package at any one time (the block size) through `VFETCH`.

Table 6.1: `MemSys5` area usage.

Area	Space	Use	Input/output		Notes
⟨`1`⟩	H	Current $\widehat{\boldsymbol{h}}$		O	`MEM5`
		Hidden sample $\boldsymbol{v} = \widehat{\boldsymbol{h}} + \Delta\boldsymbol{h}$		O	`MOVIE5`
⟨`2`⟩	H	Working space for transforms			
⟨`3`⟩	H	Default model $\boldsymbol{m}$	I		unless `DEF` > 0
⟨`4`⟩	H	Mask $\boldsymbol{p}$	I		`MASK5`
		Hidden offset $\Delta\boldsymbol{h} = \alpha^{-1/2} c\,[\boldsymbol{\mu}^{1/2}]\,\boldsymbol{B}^{-1/2}\,\boldsymbol{r}$		O	`MOVIE5`
⟨`11`⟩	V	Working space for transforms			
		Current $\widehat{\boldsymbol{f}} = \boldsymbol{C}\,\widehat{\boldsymbol{h}}$		O	`MEM5`
		Visible sample $\boldsymbol{C}\,\boldsymbol{v}$		O	`MOVIE5`
⟨`21`⟩	D	Data $\boldsymbol{D}$	I		`MEM5`
⟨`22`⟩	D	Accuracies $[\boldsymbol{\sigma}^{-1}]$	I		unless `ACC` > 0
⟨`23`⟩	D	Multipliers $\boldsymbol{w}$	I	O	Not needed as input for initial call to `MEM5`
⟨`24`⟩	D	Normalised residuals $[\boldsymbol{\sigma}^{-1}]\,(\boldsymbol{D} - \boldsymbol{F})$		O	if `METHOD(2)` $= 1$
		$(\boldsymbol{D} - \boldsymbol{F})/(\boldsymbol{D} + \boldsymbol{1})^{1/2}$		O	if `METHOD(2)` $= 2$
⟨`25`⟩	D	Working space for transforms			
⟨`26`⟩	D	Working space			
⟨`27`⟩	D	Working space			
⟨`28`⟩	D	Working space			
⟨`29`⟩	D	Differential responsivity			Needed only if response is nonlinear

6.2 Initialisation: MEINIT

Before using any of the routines of the package, the user must

```
CALL MEINIT
```

This is a compulsory first call, which initialises the system to a defined state. It may also be called to re-initialise the package completely, erasing the set-up and access to results from the previous run.

6.3 Area handling and overlaying

The package's storage handling has been designed to leave as much freedom as possible in the way that areas may be stored when not being accessed.

All the areas must be kept separate, except for areas ⟨2⟩, ⟨11⟩, and ⟨25⟩. Within the package, all the transforms $\boldsymbol{C}$ and $\boldsymbol{C}^T$ between hidden space and visible space are between areas ⟨2⟩ and ⟨11⟩, and the transforms $\boldsymbol{R}$ and $\boldsymbol{R}^T$ between visible space and data space are between ⟨11⟩ and ⟨25⟩. To the extent that the user's transforms can be done in place, these three areas may be overlaid.

The maximum number of areas to which the package will request simultaneous access is four, in which case one of the areas will always be the transform area ⟨25⟩. Hence the absolute minimum required in ST is workspace for four blocks, one for part of each of the accessed areas. If storage is limited to this, the user must transform directly between his external areas, although the whole of ST would be available as working space. For convenience, though, transforms are usually performed in memory within ST wherever possible. Assuming that all the other areas are stored elsewhere, ST must then be long enough to hold the transform areas, plus workspace for three more blocks. It is, of course, generally more convenient to store all the areas directly in ST if this can be done.

It is possible to 'switch out' one or more observations from the data vector $\boldsymbol{D}$, by setting the corresponding element(s) of the accuracies in area ⟨22⟩ to zero. The system will then run as though these elements of $\boldsymbol{D}$ were absent. Since area ⟨22⟩ contains reciprocal standard deviations, setting an element $1/\sigma_k$ to zero effectively sets the standard deviation of D_k to infinity, and so D_k has no influence on the calculation.

One or more elements of $\boldsymbol{h}$ can be 'switched out', i.e., set to zero, by setting the corresponding elements of the default model $\boldsymbol{m}$ in area ⟨3⟩ to zero.

Chapter 7

The vector library

`MemSys5` is supplied as two source-code modules, `memsys5` with its `INCLUDE` file, and `vector`. The central module `memsys5`, hereafter referred to as the ‘system’, performs all scalar calculations, all high-level manipulation of the storage areas and the production of all diagnostics. This module is independent of any hardware considerations.

All the actual vector arithmetic, and the addressing needed to control it, is handled by the subroutines in the `vector` module, comprising the vector library. This means that it is relatively straightforward to transfer `MemSys5` to array processors, parallel systems, or other hardware architecture. The system allows areas to be accessed piecemeal in blocks, largely under the user’s control so that best use can be made of a possibly limited amount of memory. Sequences of calls to the vector library always form a chain, in which one or more vector operations are performed on successive blocks of up to four areas at a time (in which extreme case one of the areas is always the transform area ⟨25⟩). The following example illustrates a typical sequence of calls. In it, the contents of ⟨23⟩ and ⟨24⟩ are being used to calculate new contents for ⟨24⟩ and ⟨27⟩.

Enter vector chain: begin at the start of each area.
Repeat ...

- `CALL` fetch next block of ⟨24⟩ (input values needed)
- `CALL` fetch next block of ⟨27⟩
- `CALL` fetch next block of ⟨23⟩ (input values needed)

- `CALL` first vector operation on block of ⟨23⟩,⟨24⟩,⟨27⟩
- `CALL` second vector operation on block of ⟨23⟩,⟨24⟩,⟨27⟩
- ⋮
- `CALL` last vector operation on block of ⟨23⟩,⟨24⟩,⟨27⟩

- `CALL` store block of ⟨23⟩
- `CALL` store block of ⟨24⟩ (output values to be preserved)
- `CALL` store block of ⟨27⟩ (output values to be preserved)

... until the areas are exhausted: exit vector chain.

The system will never mix calls between the different spaces (hidden, visible and data), so that all the areas in a chain should be of the same length. Every “fetched” area will subsequently be “stored”, and conversely every area to be “stored” will first be “fetched”. As illustrated, however, the calls to “fetch” the areas may be in any order, though the calls to “store” them will be in serial order.

7.1 Area access routines VFETCH and VSTORE

VFETCH and VSTORE control the addressing, and are the only subroutines which require access to the user's pointer block COMMON /VPOINT/ which describes the user's storage structure. Hence, if the format of /VPOINT/ becomes inappropriate or inadequate, the only subroutines which need to be changed are VFETCH and VSTORE. As supplied, VFETCH and VSTORE have a rather complicated form which encodes dynamic control of blocked input/output to external storage outside the ST array. Dynamic control is in the interests of efficient I/O, but is not essential for proper operation of the system.

Should the user wish to alter VFETCH and VSTORE, he must adhere to their specifications:

```
SUBROUTINE VFETCH(ST,J,IOFF,ACTION, KCORE,LENGTH, MORE)
REAL ST(0:*)                    arithmetic workspace array
INTEGER J         Input:   area number
INTEGER IOFF      Input:   first element of current block
LOGICAL ACTION    Input:   contents to be read?
INTEGER KCORE     Output:  start address of current block
INTEGER LENGTH    Output:  number of elements in current block
INTEGER MORE      Input: start of new chain?

SUBROUTINE VSTORE(ST,J,IOFF,ACTION, KCORE,LENGTH, MORE)
REAL ST(0:*)                    arithmetic workspace array
INTEGER J         Input:   area number
INTEGER IOFF      Input:   first element of current block
LOGICAL ACTION    Input:   contents to be preserved?
INTEGER KCORE     Input:   start address of current block
INTEGER LENGTH    Input:   number of elements in current block
INTEGER MORE      Output:  any more elements?
```

VFETCH enables access to a single block of area ⟨J⟩, starting at element IOFF. This block may comprise either all or just part of the area. IOFF is controlled by the system. It will be zero for the first element of ⟨J⟩, and will thereafter be incremented by LENGTH until all the elements of ⟨J⟩ have been accessed. VFETCH must return a storage address KCORE pointing to where the block may thereafter be found in ST. VFETCH must also return LENGTH, being the number of elements in the block. Thus on return from VFETCH, elements IOFF, ..., IOFF + LENGTH − 1 of ⟨J⟩ are to be found in ST(KCORE), ..., ST(KCORE + LENGTH − 1). Within a particular loop of a vector chain, the system will keep supplying the same offset IOFF, and VFETCH must keep returning the same LENGTH.

If ACTION is .TRUE. then the system needs the current block of ⟨J⟩ for input, but if ACTION is .FALSE. the current values values are not needed. MORE is a status flag. On entry to each vector chain, the system will supply MORE = 0, but (subject to a restriction on its value as an output from VSTORE) the user is then free to use this flag for his own purposes.

VSTORE relinquishes access to a previously "fetched" block of ST. For each area number J, the offset IOFF, start address KCORE and length LENGTH will all be the same as on return from the previous VFETCH call. If ACTION is .TRUE., then the contents of the block have been altered and must be preserved, but if ACTION is .FALSE. any new values need not be preserved. The system will, however, assume that any old values which were unchanged by the vector calls will remain in place. A call to VSTORE with ACTION = .FALSE. does not mean that the old values of the block may be altered. On exit from the final VSTORE in a loop, the system will interrogate the value of

MORE. A zero value of MORE signals the end of a chain to the system, and VSTORE must provide this only at the correct time.

After the system has relinquished access to a block with VSTORE, it will not attempt to access it again without first enabling it with VFETCH, so the relevant part of ST may be used for other purposes if ⟨J⟩ is not being permanently stored there.

Although it is possible to include considerable sophistication in VFETCH and VSTORE if hardware considerations demand it, the routines do not have to be complicated. If all the areas are held permanently at fixed locations in ST, the following pair suffices.

```
SUBROUTINE VFETCH(ST,J,IOFF,ACTION, KCORE,LENGTH, MORE)
REAL ST(0:*)
LOGICAL ACTION
COMMON /VPOINT/ KL(40),KB(40)       Area lengths KL and base addresses KB
KCORE=KB(J)
LENGTH=KL(J)
END

SUBROUTINE VSTORE(ST,J,IOFF,ACTION, KCORE,LENGTH, MORE)
REAL ST(0:*)
LOGICAL ACTION
END
```

For the implementations of VFETCH and VSTORE supplied in vector, the COMMON block /VPOINT/ is as follows:

```
COMMON /VPOINT/ KL(40),KB(40),KA(40),KWORK,LWORK,LENREC
```

In this scheme, the array KA is used to indicate whether area ⟨J⟩ is held in the ST array in memory (KA(J) = 0) or on disc (KA(J) = 1). The array KB again holds start addresses for the areas. If area ⟨J⟩ is held in ST, then KB(J) holds the start address in ST, as above. If area ⟨J⟩ is held on disc, then KB(J) holds the start address on a direct-access file (or some equivalent) with a record length of LENREC REALs. In this case, the start address is encoded in KB(J) as an element of a (suitably large) imaginary array, with every area starting at the beginning of a new record. If any areas are stored on disc, then contiguous workspace is required in ST for disc buffers, in which the package can access the requested portions of these areas. The start address of this workspace must be supplied in KWORK, and its length in LWORK. The minimum amount of workspace required in ST is discussed in Section 6.3 on page 47.

The following code fragment illustrates how /VPOINT/ might be set up:

```
COMMON /VPOINT/ KL(40),KB(40),KA(40),KWORK,LWORK,LENREC
   ⋮
LENREC = record length (in REALs) for disc I/O, typically 1024 elements
DO 1 for each area ⟨J⟩ used
  IF ( ⟨J⟩ held in ST ) THEN
    KA(J) = 0
    KB(J) = start address within ST
  ELSEIF ( ⟨J⟩ held on disc ) THEN
    KA(J) = 1
    KB(J) = start address on disc
```

```
    ENDIF
    KL(J) =  length of ⟨J⟩
1 CONTINUE
  KWORK =  start of disc buffer area in ST
  LWORK =  length of disc buffer area in ST
  OPEN( UNIT = lun, ACCESS = 'DIRECT', ...  ) if any areas are on disc
      ⋮
  Proceed to MemSys5 calls, etc.
```

7.2 Disc-handling routines UFETCH and USTORE

The implementations of VFETCH and VSTORE in vector call the user-supplied routines UFETCH and USTORE to perform disc I/O.

```
SUBROUTINE UFETCH(ST,KCORE,KDISC,LENGTH)
REAL ST(0:*)
```

and

```
SUBROUTINE USTORE(ST,KCORE,KDISC,LENGTH)
REAL ST(0:*)
```

should read or write a block of LENGTH REALs, starting at address KCORE in ST, from or to disc, starting at disc address KDISC. The disc address KDISC will always correspond to the start of a disc record. The number of elements LENGTH to be transferred will not necessarily be an integer multiple of the record length LENREC, but incomplete records will be read or written only at the end of a storage area.

Examples of these routines are as follows:

```
      SUBROUTINE UFETCH(ST,KCORE,KDISC,LENGTH)
      REAL ST(0:*)
      COMMON /VPOINT/ KL(40),KB(40),KA(40),KWORK,LWORK,LENREC
      DO 1 I=0,(LENGTH-1)/LENREC
        READ (UNIT = lun, REC = 1+KDISC/LENREC+I)
     *          (ST(KCORE+K),K=I*LENREC,MIN((I+1)*LENREC-1,LENGTH))
    1 CONTINUE
      END
```

and

```
      SUBROUTINE USTORE(ST,KCORE,KDISC,LENGTH)
      REAL ST(0:*)
      COMMON /VPOINT/ KL(40),KB(40),KA(40),KWORK,LWORK,LENREC
      DO 1 I=0,(LENGTH-1)/LENREC
        WRITE (UNIT = lun, REC = 1+KDISC/LENREC+I)
     *          (ST(KCORE+K),K=I*LENREC,MIN((I+1)*LENREC-1,LENGTH))
    1 CONTINUE
      END
```

7.3 Arithmetical vector routines

All the vector arithmetic controlled by the package, apart from the transforms, is performed by the arithmetic routines in the vector library. Only these routines have to be replaced if the arithmetic is to be performed somewhere other than in the array `ST` in memory, on an array processor or such hardware.

Several of these arithmetical routines are simple, and commonly available in external libraries supplied with array processing hardware.

`VFILL` fills a block with a scalar.

`VMOV` moves a block to another location.

`VSWAP` interchanges two blocks.

`VMUL` multiplies two blocks.

`VDIV` divides two blocks. No denominator will be zero.

`VLOG` returns the logarithm of a strictly positive block.

`VSQRT` returns the square root of a non-negative block.

`VSIGN` returns the signs ($+1.0$ or -1.0) of a block.

`VSADD` adds a scalar to a block.

`VSMUL` multiplies a block by a scalar.

`VSMULA` multiplies a block by a scalar, then adds a second block.

`VSUM` returns the sum of the elements of a block. As supplied, `VSUM` uses a butterfly pattern of addition to preserve full arithmetical accuracy. Whilst this can be useful on occasion, especially with large-scale problems where rounding errors might otherwise accumulate, it is seldom essential.

`VDOT` returns the scalar product of two blocks. As supplied, `VDOT`, like `VSUM`, uses a butterfly pattern of addition to preserve full arithmetical accuracy.

The '`VRAND`' group of routines is more specific to `MemSys5`, and might need special coding in terms of external library routines. Such coding might involve extra "dummy" buffers to hold blocks of intermediate results. Such buffers could safely overlay areas ⟨`27`⟩ and ⟨`28`⟩.

`VRAND` fills a block with samples from the unit normal distribution.
`VRAND0` initialises the generator with an integer seed.
`VRAND1` saves the generator state.
`VRAND2` restores the generator state.

As supplied, the generator state is stored in

```
      COMMON /VRANDC/ P1,P2,P3,P4,P5,P6
```

Finally, the last arithmetical routine `VMEMX` sets any nonlinear corrections to the responsivity transform (`METHOD(3) = 2`). As supplied, this routine calls the user's `SUBROUTINE UMEMX` for each element of the area. For efficient vectorisation, the code for `UMEMX` should be pulled into an appropriately recoded version of `VMEMX`.

`VMEMX` sets the nonlinear option.

Chapter 8

Transform routines

8.1 Transform subroutines OPUS, TROPUS, and UMEMX

The user must supply two subroutines OPUS and TROPUS which transform between visible space and data space.

```
SUBROUTINE OPUS(ST,JM,JN)
```

should apply $\boldsymbol{R}$ to the visible-space distribution from area ⟨JM⟩ to calculate the corresponding data-space quantities

$$F_k = \sum_{j=1}^{M} R_{kj} f_j \qquad \text{or} \qquad \boldsymbol{F} = \boldsymbol{R}\boldsymbol{f}$$

and write them to data area ⟨JN⟩. Areas ⟨JM⟩ and ⟨JN⟩ can be, and usually are, of different length.

```
SUBROUTINE TROPUS(ST,JN,JM)
```

should apply $\boldsymbol{R}^T$ to data-space quantities from area ⟨JN⟩ to calculate the corresponding visible-space quantities

$$f_j = \sum_{k=1}^{N} R_{kj} F_k \qquad \text{or} \qquad \boldsymbol{f} = \boldsymbol{R}^T\boldsymbol{F}$$

and write them to area ⟨JM⟩.

In these formulae, $\boldsymbol{R}$ is the response function of the apparatus producing the data, which is assumed constant, as for a linear experiment giving

$$\boldsymbol{D} = \boldsymbol{R}\boldsymbol{f} \pm \boldsymbol{\sigma}$$

The package always calls OPUS and TROPUS with JM = 11 and JN = 25, but the explicit arguments remain in place in order to preserve flexibility and compatibility with earlier maximum entropy programs.

Clearly it will usually be convenient to have both area ⟨JM⟩ and area ⟨JN⟩ in ST, and possibly overlaid if the transform coding allows it, so that a simple OPUS routine could be constructed as follows.

```
SUBROUTINE OPUS(ST,JM,JN)
REAL ST(0:*)
```

```
      COMMON /VPOINT/ KL(40),KB(40)
      CALL VOPUS(ST,KB(JM),KB(JN),KL(JM),KL(JN))
      END
```

```
      SUBROUTINE VOPUS(ST,JU,JV,M,N)
      REAL ST(0:*)
```
Matrix multiply
```
      DO 2 K = 0,N-1
        X = 0.0
        DO 1 J = 0,M-1
          X = X + TransformMatrix(K,J) * ST(JU+J)
    1   CONTINUE
        ST(JV+K) = X
    2 CONTINUE
      END
```

The routine TROPUS would be similar

```
      SUBROUTINE TROPUS(ST,JN,JM)
      REAL ST(0:*)
      COMMON /VPOINT/ KL(40),KB(40)
      CALL VTROP(ST,KB(JN),KB(JM),KL(JN),KL(JM))
      END
```

```
      SUBROUTINE VTROP(ST,JV,JU,N,M)
      REAL ST(0:*)
```
Transpose matrix multiply
```
      DO 1 J = 0,M-1
        X = 0.0
        DO 2 K = 0,N-1
          X = X + TransformMatrix(K,J) * ST(JV+K)
    1   CONTINUE
        ST(JU+J) = X
    2 CONTINUE
      END
```

These examples assume a storage scheme similar to the one suggested in Section 7.1 on page 51, in which the lengths and start addresses of areas in memory are held in KL and KB, and the elements are stored sequentially. Any quantities other than lengths and start addresses must be passed to the transform routines in a separate COMMON block.

Nonlinearities of the type

$$D_k = \Phi(F_k) \pm \sigma_k$$

where Φ is some known and reasonably well-behaved function can be tolerated by the algorithm, provided that area ⟨29⟩ is defined, and that a subroutine UMEMX is supplied which calculates the values of Φ and its derivative for a given value of F_k.

```
      SUBROUTINE UMEMX(F,PHI,DPHI)
      REAL F,PHI,DPHI
```

should set PHI $= \Phi(F)$ and DPHI $= d\Phi(F)/dF$. The package always calls UMEMX with its three arguments at different locations.

8.2 Intrinsic correlation function routines ICF and TRICF

The user is also required to supply two routines ICF and TRICF which are analogous to OPUS and TROPUS.

```
SUBROUTINE ICF(ST,JL,JM)
```

should apply the ICF operator $\boldsymbol{C}$ to the hidden-space distribution from area ⟨JL⟩ to calculate the corresponding visible-space quantities

$$f_j = \sum_{i=1}^{L} C_{ji} h_i \qquad \text{or} \qquad \boldsymbol{f} = \boldsymbol{C}\,\boldsymbol{h}$$

and write them to visible area ⟨JM⟩.

Likewise,

```
SUBROUTINE TRICF(ST,JM,JL)
```

should apply $\boldsymbol{C}^T$ to visible-space quantities from area ⟨JM⟩ to calculate the corresponding hidden-space quantities

$$h_i = \sum_{j=1}^{M} C_{ji} f_j \qquad \text{or} \qquad \boldsymbol{h} = \boldsymbol{C}^T \boldsymbol{f}$$

and place the result in hidden-space area ⟨JL⟩. There is no need for ⟨JL⟩ and ⟨JM⟩ to be of equal length for these routines.

Within the MemSys5 package, ICF and TRICF are always called with JL $= 2$ and JM $= 11$.

Examples of ICF and TRICF are supplied with the MemSys5 package. For the user's convenience, these can operate in-place.

Chapter 9

Diagnostics and utilities

9.1 Diagnostics routine UINFO

The MemSys5 package itself performs no I/O, but instead directs all its diagnostic output through calls to the user-supplied subroutine UINFO, which should be as follows:

```
SUBROUTINE UINFO(STRING,MLEVEL)
INTEGER MLEVEL
CHARACTER*(*) STRING
```

The character string STRING contains the diagnostic message, and MLEVEL is the diagnostic type, as shown in Table 9.1. Diagnostics in the internal box (MLEVEL = 10) are generally found to be the most useful.

Table 9.1: Coding of INFO calls with MLEVEL.

Type	MLEVEL	Interpretation
Progress	1	Names of main routines as they are called
	2	Flowchart of area input/output
Numerical	10	Descriptor with triple equals sign ===
	20	Internal technical

An internally documented example of UINFO is supplied with the MemSys5 package.

When UINFO is called with MLEVEL = 1, STRING will contain the name of one of the main internal routines of the package, together with any area numbers which that routine takes as arguments. For example,

```
MeCopy(25,21)
```

indicates that MECOPY has been called to copy the contents of area ⟨25⟩ to ⟨21⟩.

When `UINFO` is called with `MLEVEL` = 2, `STRING` will contain one line of the flowchart showing area usage by the package in the vector operation just finished. It is often helpful to read this in conjunction with the preceding `MLEVEL` = 1 call, which indicates the subroutine involved. For example, the flowchart string following the above call was:

```
.......... .......... W...R..... ..........
```

This represents the status of each of the package's 40 areas, arranged in the four groups of ten: ⟨1⟩ to ⟨10⟩, ⟨11⟩ to ⟨20⟩, ⟨21⟩ to ⟨30⟩ and ⟨31⟩ to ⟨40⟩. The area is represented by a dot '`.`' if no operation has occurred. If the area has been read, then '`R`' appears. In the above example, `MECOPY` has read the contents of area ⟨25⟩. If the area has been written to, then '`W`' appears. In this example, `MECOPY` has written to area ⟨21⟩. If the contents of the area have been read and written in the same vector operation, then '`U`' (`U`pdate) appears. Finally, a semicolon '`:`' would denote an area used as temporary workspace.

`UINFO` calls with `MLEVEL` = 10 contain the main scalars calculated by `MEM5`, for example

```
Entropy ===  -3.3419E+03    Test ===  .0004    Chisq ===  3.3359E+01
```

These values are returned also in the output arguments of `MEM5`, in this case `S`, `TEST` and `CHISQ`.

`UINFO` calls with `MLEVEL` = 20 contain detailed technical diagnostic information about the package's internal functioning. This will generally be of little concern to the user.

9.2 Transform checking subroutine `MEMTRQ`

A common error in programming transform routines is inconsistency between `ICF` and `TRICF`, or `OPUS` and `TROPUS`, which arises if these routines do not represent each other's transpose. Matrices $\boldsymbol{Q}$ and $\boldsymbol{T}$ are each other's transpose if and only if

$$\boldsymbol{u}^T \boldsymbol{T}\, \boldsymbol{v} = \boldsymbol{v}^T \boldsymbol{Q}\, \boldsymbol{u} \qquad \text{for all vectors } \boldsymbol{u},\ \boldsymbol{v}.$$

Subroutine `MEMTRQ` (acronym `MEM TR`opus `Q`uery) enables this relationship to be checked for individual pairs of vectors $\boldsymbol{u}$, $\boldsymbol{v}$ in areas ⟨1⟩ and ⟨26⟩ respectively.

```
CALL MEMTRQ(ST,ISEED,ERR)
```

If the `INTEGER ISEED` is greater than zero, then `MEMTRQ` will use the package's internal random generator to set up areas ⟨1⟩ and ⟨26⟩. If `ISEED` is less than or equal to zero, `MEMTRQ` will use ⟨1⟩ and ⟨26⟩ as supplied by the user.

The routine will return the dimensionless `REAL ERR`:

$$\texttt{ERR} = \frac{|\, \boldsymbol{u}^T \boldsymbol{y} - \boldsymbol{v}^T \boldsymbol{x}\, |}{(|\boldsymbol{u}|\,|\boldsymbol{y}|\,|\boldsymbol{v}|\,|\boldsymbol{x}|)^{1/2}}$$

where

$$\boldsymbol{x} = \text{Opus}\,(\,\text{ICF}\,(\boldsymbol{u})\,) \qquad \text{and} \qquad \boldsymbol{y} = \text{TrICF}\,(\,\text{Tropus}\,(\boldsymbol{v})\,)\,.$$

If `OPUS`/`TROPUS` and `ICF`/`TRICF` have been consistently coded, the value of `ERR` should be appropriate to the rounding errors expected in the transforms. Most inconsistencies show up as considerably larger errors. Sometimes, addressing errors show up only after a second call to a routine, so that a repeated call to `MEMTRQ` is recommended.

Of course, `MEMTRQ` cannot detect all errors. Subroutines can be consistent, but still wrong.

9.3 Save and restore utility: MESAVE and MEREST

This facility allows the user to stop a MemSys5 run after any iterate, and preserve outside the package the information necessary to restart the run from exactly the stage at which it was halted. It can also be used, for example, to store the information from a converged MEM5 run so that one of the other routines, such as MOVIE5 or MASK5, can be run from a separate program.

To save the information from a run, the user should

```
CALL MESAVE
```

This will cause a call to the user-supplied routine USAVE which should be as follows:

```
SUBROUTINE USAVE(INTS,NINTS,REALS,NREALS)
INTEGER NINTS,NREALS,INTS(NINTS)
REAL REALS(NREALS)
```

The user is then required to save the contents of the arrays INTS, and REALS externally, perhaps on disc. The values of the input arguments NINTS and NREALS will be approximately 9 and 33 respectively, depending on the MemSys5 version being used. The vector storage areas ⟨3⟩ (if DEF $\leq$ 0), ⟨21⟩, ⟨22⟩ (if Poisson or ACC $\leq$ 0), ⟨23⟩ (and ⟨29⟩ if using nonlinear response) must also be preserved at this stage. The user will also need to save his storage addressing information in /VPOINT/, as well as any other variables passed in COMMON blocks to the transform routines. A call to MESAVE during a run has no effect on that run, which may be continued if desired.

In order to restart the package, the user should

```
CALL MEREST
```

This will result in a call to the user-supplied routine

```
SUBROUTINE UREST(INTS,NINTS,REALS,NREALS)
INTEGER NINTS,NREALS,INTS(NINTS)
REAL REALS(NREALS)
```

in which NINTS and NREALS are input arguments. The user should restore to the arrays INTS and REALS the values supplied by USAVE. The contents of the vector storage areas listed above must also be restored, along with /VPOINT/ and any other COMMON blocks.

Examples of USAVE and UREST are supplied with the package.

9.4 Error messages

All the errors detected within the MemSys5 package are deemed to be fatal, and so if an error is encountered the package will STOP with one of the following error messages:

`Illegal MEMRUN value`

MEMRUN is an INTEGER switch for initialisation or continuation of a MEM5 run, and must be set to a value between 1 and 4 inclusive when calling MEM5. See page 67.

`Illegal METHOD(0) value`

METHOD(0) is an INTEGER which determines the stopping criterion, and must be set to a value between 1 and 4 inclusive when calling MEMSET. See page 65.

`Illegal METHOD(1) value`

METHOD(1) is an INTEGER which determines the type of entropy to be used, and must be set to a value between 1 and 5 inclusive when calling MEMSET. See page 65.

`Illegal METHOD(2) value`

METHOD(2) is an INTEGER which determines the type of likelihood (Gaussian or Poisson) to be used, and must be set to either 1 or 2 before calling MEMSET. See page 65.

`Illegal METHOD(3) value`

METHOD(3) is an INTEGER which determines the type of response (linear or nonlinear) to be used, and must be set to either 1 or 2 before calling MEMSET. See page 66.

`Method incompatible with Poisson statistics`

Poisson likelihood cannot be used with automatic noise scaling.

`Illegal NRAND value`

NRAND is an INTEGER which sets the number of random vectors used to calculate G and Evidence for the classic maximum entropy stopping criteria. If one of these criteria is used, NRAND must be set greater than zero before calling MEMSET. See page 66.

`Illegal RATE value`

RATE is a REAL distance limit for a MEM5 iterate. It must be set greater than 0.0 before calling MEMSET. See page 66.

`Illegal AIM value`

AIM is the required REAL value of the stopping criterion. It must be set greater than or equal to 0.0 before calling MEMSET. See page 66.

`Illegal UTOL value`

UTOL is the dimensionless REAL tolerance demanded of various computations. It must be set between 0.0 and 1.0 inclusive before calling MEMSET. See page 66.

`Illegal area number`

MemSys storage areas are numbered from 1 to 40 inclusive. See page 45.

`TOLerance determination failed - see MEINIT source code`

The package attempts to estimate the computer's arithmetic tolerance TOL, i.e., the smallest positive number for which `(1.0+TOL) .GT. 1.0` (or `(1.0D0+TOL) .GT. 1.0D0` for DOUBLE PRECISION) is .TRUE., in MEINIT. If this calculation fails, then TOL may be set explicitly by editing the source code in MEINIT, for example `TOL = 1.0E-7` for REAL or `TOL = 1.0D-16` for DOUBLE PRECISION. The exact value is not critical.

`MEVRND needs separate output and work areas`

When generating correlated random vectors, MEVRND requires an area as workspace. This area must not be the same one as the output area. This error will not arise from the MemSys5 package's use of the routine.

In addition the following errors are detected by the storage-handling routines:

`Inconsistent lengths`

All the areas involved in a vector operation must be of the same length.

`Not enough space for buffers`

If areas are stored outside the workspace array `ST`, then `LWORK` must be large enough to allow temporary buffers to be set up in `ST`. See page 51.

`Stack overflow`

The maximum number of buffers in the workspace array `ST` which can be used simultaneously is four, and requests to `VFETCH` for further buffers will cause this error. It will not arise from the `MemSys5` package's use of this routine. See page 51.

`Stack underflow`

This indicates an error in the use of the storage-handling routines, such as a call to `VSTORE` which was not preceded by a corresponding call to `VFETCH`. This error will not arise from the `MemSys5` package's use of these routines. See page 51.

9.5 Miscellaneous subroutines

`MECOPY` and `MESWAP`

The user may find the following routines useful for copying data from one area to another and interchanging the contents of two areas.

```
CALL MECOPY(ST,J,K)
```

will copy the contents of area ⟨`J`⟩ to ⟨`K`⟩. If `J` = `K`, then no operation occurs. A common use of this facility is for loading an externally-stored area. The values may easily be read into a transform area held in memory in `ST`, and then copied to a less accessible destination area with `MECOPY`. Thus the data file could be read into ⟨`25`⟩, before copying it to ⟨`21`⟩ which might be stored on disc.

```
CALL MESWAP(ST,J,K)
```

will interchange the contents of areas ⟨`J`⟩ and ⟨`K`⟩. If `J` = `K`, then no operation occurs. `MESWAP` can be used for diagnostic purposes, swapping a disc area into memory for inspection, before returning it non-destructively to disc. The `MemSys5` package itself, however, makes no use of any `SWAP` routines.

`MEMICF` and `MTRICF`

```
CALL MEMICF(ST,JL,JM)
```

will apply the ICF operator $\boldsymbol{C}$ to ⟨`JL`⟩, putting the result in ⟨`JM`⟩. The routine uses the standard transform areas ⟨`2`⟩ and ⟨`11`⟩ as workspace. This enables a hidden-space area to be viewed in visible space. Conversely

```
CALL MTRICF(ST,JM,JL)
```

will apply $\boldsymbol{C}^T$ to ⟨`JM`⟩, putting the result in ⟨`JL`⟩, again using ⟨`2`⟩ and ⟨`11`⟩ as workspace.

Chapter 10

Specifications of the major subroutines

10.1 MEMSET arguments

The MemSys control variables are set by

```
CALL MEMSET(METHOD,NRAND,ISEED,AIM,RATE,DEF,ACC,UTOL)
```

which must be called before MEM5. The package's internal random generator is also re-initialised by every MEMSET call. All the arguments are input variables. In accordance with FORTRAN typing, METHOD, NRAND and ISEED are INTEGERs, and the other arguments are REALs.

Input variables should be set as follows:

METHOD is an INTEGER array for setting options, encoded as follows: INTEGER

METHOD(0) sets the stopping criterion:

1 Classic maximum entropy
2 Classic automatic, with noise scaling
3 *Ad hoc*, α fixed and specified by AIM
4 Historic '$\chi^2 = N$' maximum entropy

METHOD(1) sets the type of entropy:

1 Standard entropy
2 Positive/negative entropy
3 Fermi–Dirac entropy
4 Quadratic 'entropy'
5 Standard entropy with $\sum_i h_i = \sum_i m_i$.

METHOD(2) sets the type of noise:

1 Gaussian statistics
2 Poisson statistics

METHOD(3) allows some nonlinearity

1 response is linear in $\boldsymbol{h}$

2 response is nonlinear, given by SUBROUTINE UMEMX

NRAND	is the number of random vectors to be used to calculate the Evidence. NRAND is needed for the "classic" options METHOD(0) = 1 and METHOD(0) = 2, and is normally set to 1.	INTEGER, > 0 if used
ISEED	is the seed for the internal pseudo-random number generator, which is re-initialised by every MEMSET call.<br>ISEED is needed for the "classic" options METHOD(0) = 1 and METHOD(0) = 2, and is normally not changed between iterates.	INTEGER
AIM	is the required value of the stopping criterion $1/\Omega$.<br>For "classic" and "historic" options METHOD(0) = 1, 2, and 4, AIM ought to be set to 1.0. When setting α explicitly ("*ad hoc*" METHOD(0) = 3), AIM is the required value of α. In all cases, setting AIM to a smaller value forces the algorithm to proceed further down the trajectory towards fitting the data more closely.	REAL ≥ 0.0
RATE	is a dimensionless distance limit.<br>RATE gives a conservative upper bound, normally set to a value of order 1.0, to the trust region radius $r_0 =$ RATE $\times (\mathrm{trace}[\boldsymbol{\mu}])^{1/2}$.	REAL > 0.0
DEF	gives the default level.<br>If DEF > 0.0, then $m_i =$ DEF (or $m_i/(1-m_i) =$ DEF for Fermi–Dirac entropy) for $i = 1, 2, \ldots, L$. Otherwise, $\boldsymbol{m}$ (or $\boldsymbol{m}/(\boldsymbol{1}-\boldsymbol{m})$ for Fermi–Dirac) is assumed to be set up in area $\langle 3 \rangle$. Individual values of m_i must be positive or zero.	REAL
ACC	gives the accuracies, for Gaussian likelihood only.<br>If ACC > 0.0, then $1/\sigma_k =$ ACC for $k = 1, 2, \ldots, N$. Otherwise, $\boldsymbol{\sigma}^{-1}$ is assumed to be set up in area $\langle 22 \rangle$. Individual values of $1/\sigma_k$ must be positive or zero.	REAL
UTOL	is a dimensionless tolerance.<br>UTOL defines the accuracy demanded of the intermediate conjugate gradient results, and of the overlap between the computed and true probability clouds. UTOL is normally set about 0.1, or less for higher precision.	REAL between 0.0 and 1.0

The current values of the variables may be recovered by

```
CALL MEMGET(METHOD,NRAND,ISEED,AIM,RATE,DEF,ACC,UTOL)
```

where the types and dimensions of each of the arguments are as for MEMSET.

10.2 MEM5 arguments

The main maximum entropy routine MEM5 is called by

```
      CALL MEM5(ST,MEMRUN,
     *          S,TEST,CHISQ,SCALE,PLOW,PHIGH,PDEV,
     *          GLOW,GHIGH,GDEV,OMEGA,ALPHA,ISTAT,NTRANS)
```

The first two arguments are input variables, and the rest are output diagnostics (none of which needs to be preserved between iterates). In accordance with FORTRAN typing, `MEMRUN`, `ISTAT` and `NTRANS` are `INTEGER`s, and the other arguments are `REAL`s. Each call may involve up to a few tens of transforms, depending on the difficulty of the problem.

The input arguments are as follows:

`ST` is the vector arithmetic workspace. `REAL`

`MEMRUN` is a switch for initialisation or continuation: `INTEGER`

`1` initialises a new run, starting from $\boldsymbol{h} = \boldsymbol{m}$.

Enter with contents of ⟨21⟩.

`2` continues the previous run.

Enter with contents of ⟨21⟩, ⟨23⟩.

`3` restarts the previous run with different `METHOD`.

Enter with contents of ⟨21⟩, ⟨23⟩.

`4` finds diagnostics only.

The arguments `S`, `TEST`, `CHISQ`, `SCALE`, `OMEGA` (and `PLOW`, `PHIGH`, `PDEV`, `GLOW`, `GHIGH`, `GDEV` if appropriate) are returned for the current $\boldsymbol{w}$. The corresponding $\boldsymbol{h}$ and $\boldsymbol{f}$ are regenerated in areas ⟨1⟩ and ⟨11⟩ respectively. The argument `ALPHA` will be set to its current value, and the components code_2 (distance limit), code_3 (change in `ALPHA`) and code_5 (cloud mismatch accuracy) of `ISTAT` will be set to zero. The remaining components of `ISTAT` will be set according to the diagnostics calculated. The total number of transforms applied since the last call with `MEMRUN` = 1 will be returned in `NTRANS`.

Enter with contents of ⟨21⟩, ⟨23⟩.

In all cases, area ⟨3⟩ must also be set up by the user if `DEF` ≤ 0.
For Gaussian errors, area ⟨22⟩ must be set up by the user if `ACC` ≤ 0.
For Poisson likelihood, ⟨22⟩ must be provided but its contents will be supplied and manipulated by the package.
For nonlinear response, ⟨29⟩ must be provided, but its contents will be supplied and manipulated by the package.
Areas ⟨3⟩, ⟨22⟩ and ⟨29⟩ must be preserved between iterates if they are in use.

Output arguments are interpreted as follows:

`S` is the entropy of the input $\boldsymbol{h}$. `REAL` ≤ 0.0

`TEST`	is $1 - \cos\theta$ for the input $\boldsymbol{h}$.<br>θ is the angle between the gradients of entropy and χ^2. `TEST` is 0 on the trajectory and less than 1 whenever the angle is acute.	`REAL`<br>between 0.0 and 2.0
`CHISQ`	is $2\mathcal{L}$ (equal to χ^2 for Gaussian errors) for the input $\boldsymbol{h}$.<br>If the noise is being rescaled (`METHOD(0)` = 2), `CHISQ` incorporates this re-scaling, and at the end of the run will equal the number of "bad" measurements.	`REAL`<br>≥ 0.0
`SCALE`	is c, the scaling factor for the noise (classic automatic only).<br>`SCALE` multiplies the initial estimates of the noise standard deviations by c, and will be 1.0 if the noise is not being rescaled (`METHOD(0)` $\neq$ 2).	`REAL`<br>> 0.0
`PLOW`	is the numerical lower limit on $\log_e \Pr(\boldsymbol{D} \mid \alpha)$ for the input $\boldsymbol{h}$ (classic options only).<br>This value incorporates any rescaling of the noise.	`REAL`
`PHIGH`	is the numerical upper limit on $\log_e \Pr(\boldsymbol{D} \mid \alpha)$ for for the input $\boldsymbol{h}$ (classic options only).<br>This value incorporates any rescaling of the noise.	`REAL`
`PDEV`	is the random vector standard deviation of $\log_e \Pr(\boldsymbol{D} \mid \alpha)$ for the input $\boldsymbol{h}$ (classic options only).<br>This value incorporates any rescaling of the noise.	`REAL`<br>≥ 0.0
`GLOW`	is the numerical lower limit on G, the number of "good" measurements for the input $\boldsymbol{h}$ (classic options only).	`REAL`<br>≥ 0.0
`GHIGH`	is the numerical upper limit on G, the number of "good" measurements for the input $\boldsymbol{h}$ (classic options only).	`REAL`<br>≥ 0.0
`GDEV`	is the random vector standard devation of G, the number of "good" measurements for the input $\boldsymbol{h}$ (classic options only).	`REAL`<br>≥ 0.0
`OMEGA`	is $\Omega \times$ `AIM`, the rescaled stopping criterion for the input $\boldsymbol{h}$. (Note the distinction between the `MEM5` argument `OMEGA` and the algebraic quantity Ω.)<br>`OMEGA` should rise close to 1.0 at the end of the run.	`REAL`<br>≥ 0.0
`ALPHA`	is α, the regularisation constant used for the new $\boldsymbol{w}$.	`REAL`<br>> 0.0

ISTAT	is a return code. ISTAT $= 0$ indicates that the run has successfully ended to within the desired tolerance UTOL. Exceptionally, this occurs immediately if OMEGA ≥ 1.0 on setup, when the routine will return with $\boldsymbol{h} = \boldsymbol{m}$, and the following diagnostics defined: S, TEST, CHISQ, SCALE, OMEGA (and PLOW, PHIGH, PDEV, GLOW, GHIGH, GDEV if appropriate) for $\boldsymbol{h} = \boldsymbol{m}$. The argument ALPHA will be undefined. Otherwise,	INTEGER ≥ 0

$$\texttt{ISTAT} = 64 \times \text{code}_6 + 32 \times \text{code}_5 + 16 \times \text{code}_4 + 8 \times \text{code}_3 + 4 \times \text{code}_2 + 2 \times \text{code}_1 + \text{code}_0.$$

For each of the binary components code_0 to code_6 a value of zero denotes 'acceptable' or 'no change', and a value of one denotes the opposite. Values of zero correspond to the following for each of the individual components:

code_0	H	cloud mismatch is less than UTOL $\times$ G
code_1	OMEGA	is within UTOL of unity
code_2	β	distance limit not invoked for ALPHA
code_3	ALPHA	not changed
code_4	TEST	≤ 1
code_5	H	cloud mismatch was found to within UTOL $\times$ H
code_6	G	'good' degrees of freedom found to within UTOL $\times$ G

NTRANS	is the number of transforms (calls to ICF/OPUS or TRICF/TROPUS) performed within the package since the last call to MEM5 with MEMRUN $= 1$.	INTEGER > 0

On each return from MEM5 the new hidden and visible distributions $\boldsymbol{h}$ and $\boldsymbol{f}$ will be in areas ⟨1⟩ and ⟨11⟩ respectively.

Note that the probabilistic quantities PLOW, PHIGH, PDEV, GLOW, GHIGH, GDEV, SCALE, and the return code code_6, are calculated only for the "classic" options METHOD(0) $= 1$ and METHOD(0) $= 2$. Otherwise, they are returned with standard harmless values PLOW $=$ PHIGH $=$ PDEV $= 0.0$, GLOW $=$ GHIGH $=$ GDEV $= 0.0$, and SCALE $= 1.0$, $\text{code}_6 = 0$.

10.3 MOVIE5 arguments

The random-sample routine MOVIE5 is called by

```
CALL MOVIE5(ST,NCORR,KSTAT,NTRANS)
```

In accordance with Fortran typing the arguments NCORR, KSTAT and NTRANS are INTEGERs. MOVIE5 is designed to be called after MEM5 has converged, and each call is likely to be about as expensive as an iterate of MEM5.

If NCORR ≤ 0 the routine will use whatever vector $\boldsymbol{r}$ is supplied in area ⟨4⟩ as input, otherwise the routine itself will set up a random vector $\boldsymbol{r}$, with optional correlation with previous such vectors. The multipliers $\boldsymbol{w}$ in area ⟨23⟩ must be retained from the preceding MEM5 run, together with ⟨3⟩

for the model (if `DEF` ≤ 0.0), ⟨22⟩ for the accuracies (if `ACC` ≤ 0.0 or `METHOD(2)` $= 2$), and the nonlinear area ⟨29⟩ (if `METHOD(3)` $= 2$).

On return, ⟨4⟩ will contain a vector

$$\Delta \boldsymbol{h} = \frac{c}{\alpha^{1/2}} \, [\boldsymbol{\mu}^{1/2}] \, \boldsymbol{B}^{-1/2} \boldsymbol{r},$$

⟨1⟩ will contain $\boldsymbol{v} = \widehat{\boldsymbol{h}} + \Delta \boldsymbol{h}$ and ⟨11⟩ will contain $\boldsymbol{C}\,\boldsymbol{v}$. If $\boldsymbol{r}$ was a random vector, $\boldsymbol{v}$ will be a random sample from the hidden posterior probability cloud and $\boldsymbol{C}\,\boldsymbol{v}$ will be the corresponding sample from the visible posterior cloud.

The variables are interpreted as follows:

`ST` is the vector arithmetic workspace. `REAL`

`NCORR` determines the random vector $\boldsymbol{r}$ used as input. `INTEGER`

If `NCORR` ≤ 0 the routine will use the user-supplied vector in area ⟨4⟩ as input, otherwise the routine itself will set up $\boldsymbol{r}$, with optional correlation with previous random vectors. If `NCORR` $= 1$, $\boldsymbol{r}$ will be uncorrelated with previous vectors, but if `NCORR` > 1, then $\boldsymbol{r}$ will be correlated with them as follows: during several successive calls to the routine with the same value of `NCORR` (greater than zero), let the mth and m'th vectors be $r_i^{(m)}$ $(i = 0, \ldots, L-1)$ and $r_i^{(m')}$ $(i = 0, \ldots, L-1)$. All values are individually from $N(0,1)$, but have covariance structure

$$\langle r_i^{(m)} r_{i'}^{(m')} \rangle = \begin{cases} \max(1 - |m - m'|/\texttt{NCORR}, 0), & \text{if } i = i'; \\ 0, & \text{if } i \neq i'. \end{cases}$$

For example, `NCORR` $= 3$ gives (for each $i = i'$) covariance

$$\langle r^{(m)} r^{(m')} \rangle = \frac{1}{3} \begin{pmatrix} 3 & 2 & 1 & 0 & 0 \\ 2 & 3 & 2 & 1 & 0 \\ 1 & 2 & 3 & 2 & 1 \\ 0 & 1 & 2 & 3 & 2 \\ 0 & 0 & 1 & 2 & 3 \end{pmatrix}$$

between the first five samples. `NCORR` $= 1$ would have given the uncorrelated identity.

`KSTAT` is the return code of evaluation of the offset vector $\Delta \boldsymbol{h}$. `INTEGER` ≥ 0

On writing

$$\texttt{KSTAT} = \quad 2 \times \text{code}_1 \quad + \quad \text{code}_0,$$

then for each of the binary components code_0 and code_1 a value of zero denotes ‘acceptable’, and a value of one denotes the opposite. Values of zero correspond to the following for each of the individual components:

code_0 conjugate gradient converged successfully
code_1 no inconsistency detected in calculation of $\Delta \boldsymbol{h}$.

NTRANS	is the number of transforms (calls to ICF/OPUS or TRICF/TROPUS) performed within the package since the last call to MEM5 with MEMRUN $= 1$.	INTEGER > 0

10.4 MASK5 arguments

The inference routine MASK5 is called by

```
CALL MASK5(ST,AMEAN,ERRLO,ERRHI,JSTAT,NTRANS)
```

The arguments (apart from ST) are all output variables. In accordance with FORTRAN typing, JSTAT and NTRANS are INTEGERs, and the other arguments are REALs. MASK5 is designed to be called after MEM5 has converged, and each call is likely to be somewhat less expensive than an iterate of MEM5. A hidden mask $\boldsymbol{p}$ must be set up in area ⟨4⟩: this will be overwritten by the routine. Commonly, this will be a visible mask, transformed into the hidden area ⟨4⟩ by a call to MTRICF. The multipliers $\boldsymbol{w}$ in area ⟨23⟩ must be preserved from MEM5, together with ⟨3⟩ for the model (if DEF ≤ 0.0), ⟨22⟩ for the accuracies (if ACC ≤ 0.0 or METHOD(2) $= 2$), and the nonlinear area ⟨29⟩ (if METHOD(3) $= 2$).

The arguments are interpreted as follows:

ST	is the vector arithmetic workspace.	REAL
AMEAN	is $\widehat{\rho}$, the value of the required integral $\widehat{\boldsymbol{h}}^T\boldsymbol{p}$.	REAL
ERRLO	is the numerical lower limit on $\delta\rho$, the standard deviation of ρ.	REAL ≥ 0.0
ERRHI	is the numerical upper limit on $\delta\rho$, the standard deviation of ρ.	REAL ≥ 0.0
JSTAT	is the return code of evaluation of $\delta\rho$:<br>0 Standard deviation was estimated accurately<br>1 Standard deviation may not have been estimated accurately	INTEGER ≥ 0
NTRANS	is the number of transforms (calls to ICF/OPUS or TRICF/TROPUS) performed within the package since the last call to MEM5 with MEMRUN $= 1$.	INTEGER > 0

For some masks $\boldsymbol{p}$, the estimation of $\delta\rho$ can be intrinsically very ill-conditioned, and prone to serious rounding error. Thus the value of the return code JSTAT should be checked. If MASK5 returns with JSTAT $= 1$ and relaxing UTOL (by a call to MEMSET) does not solve the problem, the only palliative may be full DOUBLE PRECISION operation (see 'Arithmetic accuracy' on page 88).

Chapter 11

Example of simple use

A self-documented "TOY" deconvolution simulation is supplied with copies of `MemSys5`. Schematically, it operates as follows, using the basic steps common to maximum entropy driving programs.

1. Call `MEINIT` to initialise the package.
2. Request user input (here `METHOD`, `NRAND`, `AIM`, `RATE`, `UTOL` and ICF width for flexible testing).
3. Initialise the storage area management. ("TOY" has 64-cell areas, broken into multiple blocks for illustration.)
4. Call `MEMTRQ` to check that `ICF/TRICF` and `OPUS/TROPUS` are consistent (this step is optional).
5. Read the data into area ⟨21⟩ from the appropriate disc file, set up the default $\boldsymbol{m}$ (or $\boldsymbol{m}/(\boldsymbol{1}-\boldsymbol{m})$ for Fermi–Dirac entropy) in `DEF` or ⟨3⟩, and, for Gaussian likelihood, the accuracies $[\boldsymbol{\sigma}^{-1}]$ in `ACC` or ⟨22⟩. The data are from a simulation in which a 64-cell visible-space distribution is convolved with a square point-spread function of width five cells. Three files of data are provided: `gauss.dat` in which Gaussian noise with standard deviation 10 units is added to produce the dataset shown in Figure 11.1 (for `METHOD(2)` = 1), `poiss.dat` in which Poisson noise is added (for `METHOD(2)` = 2), and `fermi.dat` in which the data are scaled suitably for Fermi–Dirac entropy (`METHOD(1)` = 3).
6. | | |
   |---|---|
   | `CALL MEMSET(METHOD,...)` | to set up the control variables for `MEM5` |
   | `MEMRUN=1` | to make `MEM5` start a new run |
   | Repeat ... | |
   | `CALL MEM5(ST,MEMRUN,...)` | to update $\boldsymbol{w}$ and maybe find $\Pr(\boldsymbol{D} \mid \alpha)$ and G |
   | `MEMRUN=2` | to make `MEM5` continue the run |
   | ... until `(ISTAT.EQ.0)` | repeat until `MEM5` has converged |
7. Interrogate and/or output the results.
8. | | |
   |---|---|
   | Repeat ... | |
   | `CALL MOVIE5(..)` | to obtain a sample from $\Pr(\boldsymbol{f} \mid \boldsymbol{D})$ |
   | ... until (finish) | repeat for as many samples as desired |
9. | | |
   |---|---|
   | Repeat ... | |
   | Set mask | to define a linear feature of $\boldsymbol{f}$ |
   | `CALL MTRICF(..)` | to generate hidden-space mask |
   | `CALL MASK5(..)` | to estimate the feature, with error bar |
   | ... until (finish) | repeat for as many features as desired |

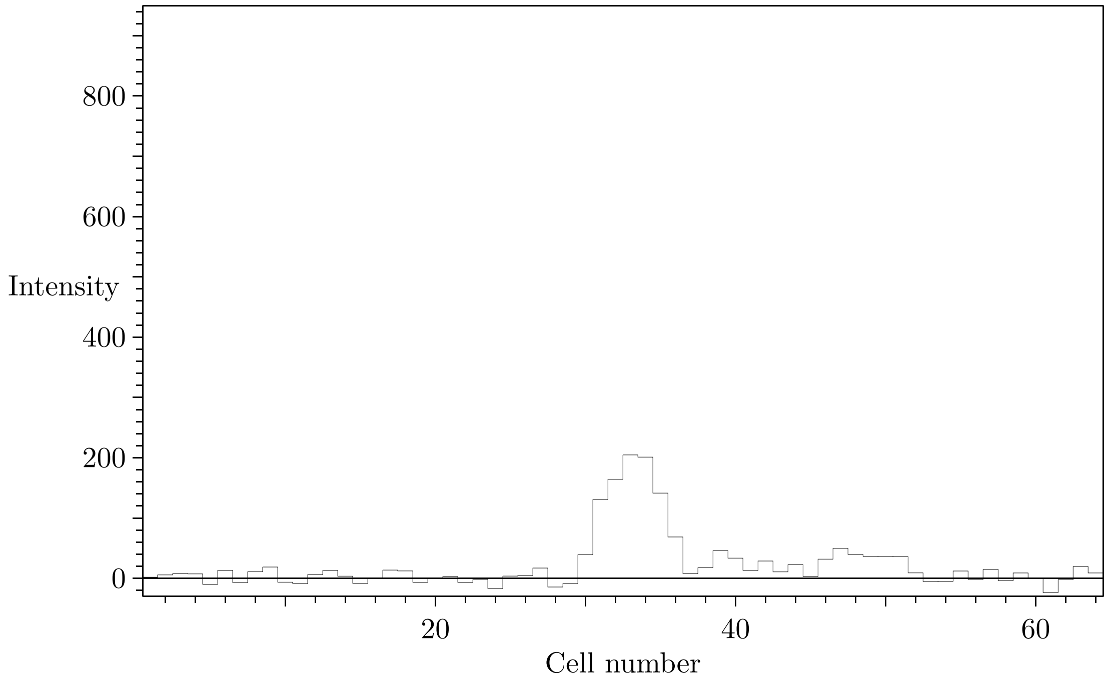

Figure 11.1: Noisy blurred data (`gauss.dat`).

11.1 Example of classic maximum entropy

For the MEM5 run to be discussed, the arguments of MEMSET were as follows:

METHOD(0)	=	2	classic with automatic noise scaling
METHOD(1)	=	1	standard entropy
METHOD(2)	=	1	Gaussian likelihood
METHOD(3)	=	1	linear response
AIM	=	1.0	stop at $\Omega = 1.0$
RATE	=	1.0	dimensionless distance limit
UTOL	=	0.01	dimensionless tolerance
NRAND	=	1	number of random vectors

The MEM5 diagnostics, reported in UINFO calls with MLEVEL = 10, were as follows, ending with statements of Good $= G$ and Evidence $= 10\log_{10}(\Pr(\boldsymbol{D} \mid \alpha))$, and a numerical printout of the central reconstruction $\widehat{\boldsymbol{f}}$.

Note: The exact values produced by TOY may differ between computers, though they should always converge to similar results.

```
Fractional OPUS/TROPUS inconsistency  =  1.6015E-08
Fractional OPUS/TROPUS inconsistency  =  4.5856E-09
Fractional OPUS/TROPUS inconsistency  =  1.0006E-08

 Iteration    1
  Entropy ===   0.0000E+00    Test === 1.0000    Chisq ===  6.4000E+01
                                                 Scale ===  4.6527E+00
  LogProb ===  -3.3657E+02    Code ===   0       Good  ===  0.0000E+00
  Omega   ===    .213183      dist ===  .6809    Alpha ===  3.7872E-01
  Ntrans  ===      7                             Code  ===  001010

 Iteration    2
  Entropy ===  -1.2811E+03    Test ===  .4970    Chisq ===  1.3494E+01
                                                 Scale ===  4.3833E+00
  LogProb ===  -3.3540E+02    Code ===   0       Good  ===  4.2742E+00
  Omega   ===    .084627      dist ===  .2615    Alpha ===  2.2674E-01
  Ntrans  ===     21                             Code  ===  001011

 Iteration    3
  Entropy ===  -8.5499E+02    Test ===  .0106    Chisq ===  2.7713E+01
                                                 Scale ===  3.2688E+00
  LogProb ===  -3.1733E+02    Code ===   0       Good  ===  5.2385E+00
  Omega   ===    .144363      dist ===  .1967    Alpha ===  1.2807E-01
  Ntrans  ===     37                             Code  ===  001011

 Iteration    4
  Entropy ===  -1.1799E+03    Test ===  .0021    Chisq ===  2.4730E+01
                                                 Scale ===  2.7741E+00
  LogProb ===  -3.0820E+02    Code ===   0       Good  ===  6.7738E+00
  Omega   ===    .172493      dist ===  .1697    Alpha ===  7.7915E-02
```

```
 Ntrans  ===     54                              Code  ===  001011

Iteration    5
 Entropy ===  -1.4613E+03    Test ===  .0032     Chisq ===  2.3842E+01
                                                 Scale ===  2.3813E+00
 LogProb ===  -2.9980E+02    Code ===   0        Good  ===  8.0827E+00
 Omega   ===    .201272      dist ===  .1696     Alpha ===  4.9607E-02
 Ntrans  ===     75                              Code  ===  001011

Iteration    6
 Entropy ===  -1.7225E+03    Test ===  .0022     Chisq ===  2.4083E+01
                                                 Scale ===  2.0691E+00
 LogProb ===  -2.9223E+02    Code ===   0        Good  ===  9.2738E+00
 Omega   ===    .232325      dist ===  .1621     Alpha ===  3.2603E-02
 Ntrans  ===     99                              Code  ===  001011

Iteration    7
 Entropy ===  -1.9524E+03    Test ===  .0015     Chisq ===  2.5585E+01
                                                 Scale ===  1.8205E+00
 LogProb ===  -2.8542E+02    Code ===   0        Good  ===  1.0242E+01
 Omega   ===    .266607      dist ===  .1628     Alpha ===  2.1939E-02
 Ntrans  ===    125                              Code  ===  001011

Iteration    8
 Entropy ===  -2.1635E+03    Test ===  .0008     Chisq ===  2.7936E+01
                                                 Scale ===  1.6225E+00
 LogProb ===  -2.7944E+02    Code ===   0        Good  ===  1.1064E+01
 Omega   ===    .306788      dist ===  .1600     Alpha ===  1.5025E-02
 Ntrans  ===    154                              Code  ===  001011

Iteration    9
 Entropy ===  -2.3606E+03    Test ===  .0009     Chisq ===  3.0943E+01
                                                 Scale ===  1.4649E+00
 LogProb ===  -2.7432E+02    Code ===   0        Good  ===  1.1787E+01
 Omega   ===    .356566      dist ===  .1639     Alpha ===  1.0433E-02
 Ntrans  ===    188                              Code  ===  001011

Iteration   10
 Entropy ===  -2.5434E+03    Test ===  .0008     Chisq ===  3.4437E+01
                                                 Scale ===  1.3398E+00
 LogProb ===  -2.7005E+02    Code ===   0        Good  ===  1.2323E+01
 Omega   ===    .416849      dist ===  .1642     Alpha ===  7.3241E-03
 Ntrans  ===    225                              Code  ===  001011

Iteration   11
 Entropy ===  -2.7154E+03    Test ===  .0007     Chisq ===  3.8179E+01
                                                 Scale ===  1.2412E+00
 LogProb ===  -2.6664E+02    Code ===   0        Good  ===  1.2802E+01
```

```
  Omega   ===    .495818      dist ===  .1637    Alpha ===  5.1881E-03
  Ntrans  ===    266                             Code  ===  001011

 Iteration   12
  Entropy ===  -2.8785E+03    Test ===  .0006    Chisq ===  4.1946E+01
                                                 Scale ===  1.1638E+00
  LogProb ===  -2.6402E+02    Code ===   0       Good  ===  1.3176E+01
  Omega   ===    .597425      dist ===  .1598    Alpha ===  3.7034E-03
  Ntrans  ===    310                             Code  ===  001011

 Iteration   13
  Entropy ===  -3.0312E+03    Test ===  .0006    Chisq ===  4.5560E+01
                                                 Scale ===  1.1034E+00
  LogProb ===  -2.6212E+02    Code ===   0       Good  ===  1.3505E+01
  Omega   ===    .732356      dist ===  .1532    Alpha ===  2.6618E-03
  Ntrans  ===    362                             Code  ===  001010

 Iteration   14
  Entropy ===  -3.1699E+03    Test ===  .0005    Chisq ===  4.8891E+01
                                                 Scale ===  1.0568E+00
  LogProb ===  -2.6091E+02    Code ===   0       Good  ===  1.3754E+01
  Omega   ===    .910269      dist ===  .0662    Alpha ===  2.2873E-03
  Ntrans  ===    419                             Code  ===  001010

 Iteration   15
  Entropy ===  -3.2234E+03    Test ===  .0000    Chisq ===  5.0331E+01
                                                 Scale ===  1.0386E+00
  LogProb ===  -2.6027E+02    Code ===   0       Good  ===  1.3749E+01
  Omega   ===   1.005892      dist ===  .0056    Alpha ===  2.2873E-03
  Ntrans  ===    469                             Code  ===  000000

Good     =      13.7 = [      13.7 ,      13.8 ] +-      4.7
Evidence =   -1130.4 = [   -1130.4 ,   -1130.3 ] +-     34.2 decibels

   3.1341E+01    1.0469E+01    1.4083E-02    3.4742E-03
   2.1907E-02    4.8423E+00    1.0920E+01    7.4004E+00
   1.5856E+00    3.1543E+00    5.4914E+00    2.6403E+00
   1.3046E-01    3.3589E+00    7.0285E+00    4.4637E+00
   1.8152E+00    5.2656E+00    7.1888E+00    3.0551E+00
   1.2920E-03    1.3445E-07    2.7727E-05    1.2424E-01
   5.8249E-01    8.4300E-01    4.4108E-01    6.2134E-02
   5.7649E-03    4.2275E-06    1.3015E+01    2.0464E+02
   4.1466E+02    2.7147E+02    5.2480E+01    4.2455E+00
   3.7920E+00    8.7204E+00    2.8976E+01    5.6673E+01
   4.3522E+01    1.0696E+01    1.3838E-02    1.1046E+01
   2.7999E+01    2.3419E+01    1.3818E+01    5.4434E+01
   8.8791E+01    4.3318E+01    1.8904E+00    7.3660E-01
   6.9818E-01    2.9075E-01    6.1664E-01    7.2216E+00
```

```
1.2752E+01    6.1454E+00    3.3634E-02    2.9892E-06
2.1309E-05    1.0211E-04    1.9958E-01    5.9834E-01
```

The first diagnostic is the consistency check, verifying that `OPUS/ICF` and `TRICF/TROPUS` are apparently each other's transpose to `REAL` precision accuracy. The check is done three times, not only to use different seed vectors, but also to ensure that `ICF`, `TRICF`, `OPUS` and `TROPUS` are at least reasonably non-destructive.

This simple deconvolution problem was solved to within the desired 1% fractional tolerance in 15 iterates and 469 transforms of `OPUS/ICF` or `TRICF/TROPUS`. The maximum entropy diagnostics, flagged by their triple equals sign `===` , generally correspond to the appropriate `MEM5` arguments, apart from the following:

`Code` (single-digit)	$= \mathrm{code}_6$
`Code` (six-digit)	$= \mathrm{code}_5$ to code_0 (reading from left to right)
`LogProb`	$= (\mathtt{PLOW} + \mathtt{PHIGH})/2 = \log_e \Pr(\boldsymbol{D} \mid \alpha)$
`Good`	$= (\mathtt{GLOW} + \mathtt{GHIGH})/2 = G$
`dist`	$= \mathtt{RATE} \times \lvert\delta \boldsymbol{r}\rvert / r_0$

The `Entropy`, zero on entry, falls away from this global maximum to a value of -3223. `Alpha`, which as printed is the value of α on exit, used to calculate the new multipliers $\boldsymbol{w}$, likewise falls steadily to its final value of 0.0023. (There might have been an occasional pause in α flagged by $\mathrm{code}_3 = 0$ in a problem of this difficulty, though this run happened to proceed continuously.) `Test`, after the first couple of iterates, remains comfortably small throughout the run. `Omega` rises steadily to 1.006 (tolerably close to 1). The return `Codes` in `ISTAT` give overall information on each iterate:

$\mathrm{code}_6 = 0$	records that G was always found to acceptable accuracy,
$\mathrm{code}_5 = 0$	records that the cloud mismatch H was always found to acceptable accuracy,
$\mathrm{code}_4 = 0$	records that `TEST` never exceeded 1.0,
$\mathrm{code}_3 = 1$	until the end records steady decrease in α,
$\mathrm{code}_2 = 0$	records that the distance limit was never invoked for α,
$\mathrm{code}_1 = 1$	until the end records the stopping condition $\mathtt{OMEGA} = 1.0$ not being reached,
$\mathrm{code}_0 = 1$	until the end records the cloud overlap criterion not being met.

At the end of the run, `Good` and `Evidence` are reported. Their numerical lower and upper limits are given in the form `[13.7,13.8]`. The width of this interval depends on the convergence criterion for conjugate gradient iteration, which is controlled by `UTOL`. The random vector standard deviation is also given, in the form `+-4.7`. This standard deviation may be decreased by increasing `NRAND`. The `Evidence` is expressed in decibels (dB), so that $10 \times -260.27/\log_e 10 = -1130.4\,\mathrm{dB}$.

Points to note are that `LogProb` does indeed rise to a maximum value at the end of the run, as it should because the final value of α ought to be the most probable. With automatic noise scaling in operation, χ^2 on entry rescales to the number of data (here 64), and when the stopping criterion is satisfied it measures the number of "bad" measurements (here 50). Conversely, the number of "good" measurements rises from 0 to 14, verifying the prediction (page 31) that when the stopping criterion is satisfied

$$\chi^2 + G = N.$$

The diagnostic `Scale` is the rescaling coefficient $\mathtt{SCALE} = c$. The final value $c = 1.0386$ suggests that the noise, which was initially assumed to have standard deviation 10.0, should more properly be assigned a standard deviation of 10.386. As it happens, the noise was drawn from a distribution with standard deviation 10.000. However, the prediction of 10.386 is unusually accurate for such a

small-scale problem, because with only 50 “bad” measurements to define c, a proportional error of order $50^{-1/2} \approx 14\%$ might be expected.

The reconstruction $\widehat{\boldsymbol{f}}$ at the centre of the posterior cloud for the classic maximum entropy analysis of the data shown in Figure 11.1 is shown in Figure 11.2, and the hidden-space distribution $\widehat{\boldsymbol{h}}$ from which it was derived is shown in Figure 11.3. The ICF was in this case a three-cell triangular point-spread function ($1/4$, $1/2$, $1/4$), and comparison of Figures 11.2 and 11.3 shows that its effect is to make $\boldsymbol{f} = \boldsymbol{C}\,\boldsymbol{h}$ a ‘smoother’ version of $\boldsymbol{h}$, by introducing local correlations between the elements of $\boldsymbol{f}$.

Inspection of $\widehat{\boldsymbol{f}}$ in Figure 11.2 shows a large signal around cell 33, some more structure around cell 49, a certain amount of signal between these two positions, and not much outside. Comparing this with the data in Figure 11.1, and noting that the noise standard deviation was 10 units, these features of the reconstruction are entirely reasonable. Nonetheless, inspection of $\widehat{\boldsymbol{f}}$ alone gives no indication of the reliability of the structures seen. To gain an impression of which features are likely to be ‘real’, we turn to the four uncorrelated `MOVIE5` samples from the posterior cloud $\Pr(\boldsymbol{f} \mid \boldsymbol{D})$, shown in Figures 11.4 to 11.7. From these we see immediately that the major peak in cells 32 to 34 is present in similar form in all four samples, which suggests that it is probably reliable. Indeed, the proportion of samples in which a feature appears is a direct estimate of the probability of that feature being present. There is also a secondary peak around cells 48 to 50 which, while slightly less clearly defined than the peak in cells 32 to 34, is also consistently present. There is also some structure present in all four samples between these two peaks which varies from sample to sample. Outside this region, there appears to be little consistent structure. All four `MOVIE5` samples show some negative values. As explained on page 25, such values signal a local breakdown of the Gaussian approximation, which we are presently unable to quantify.

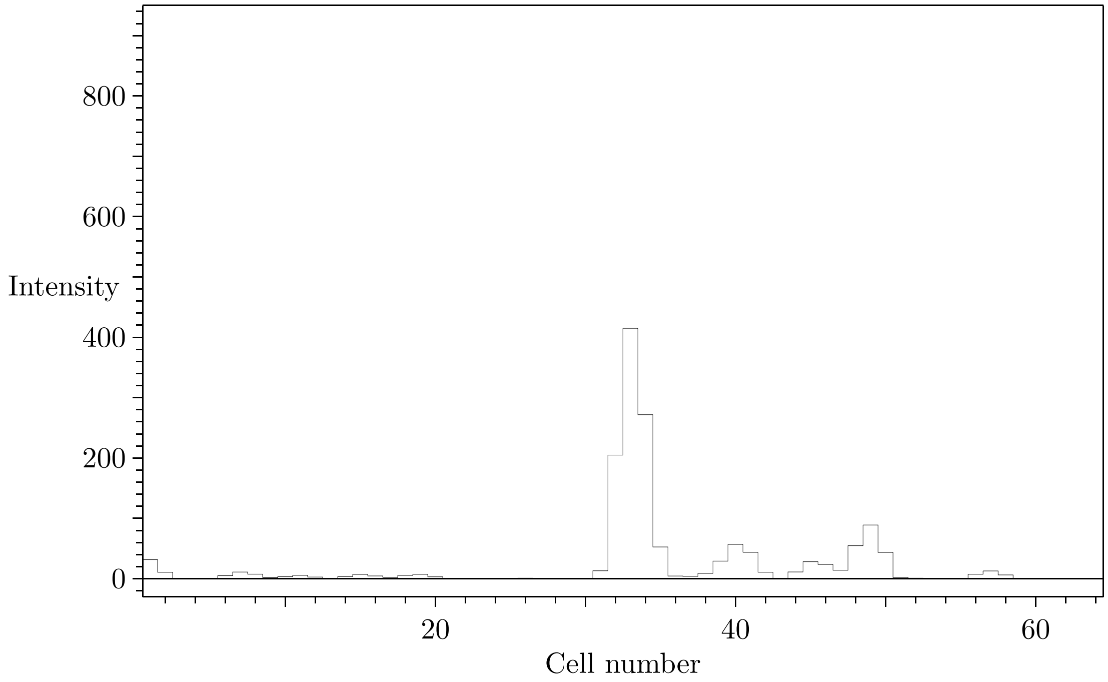

Figure 11.2: Classic maximum entropy reconstruction $\widehat{\boldsymbol{f}}$ from the data of Figure 11.1.

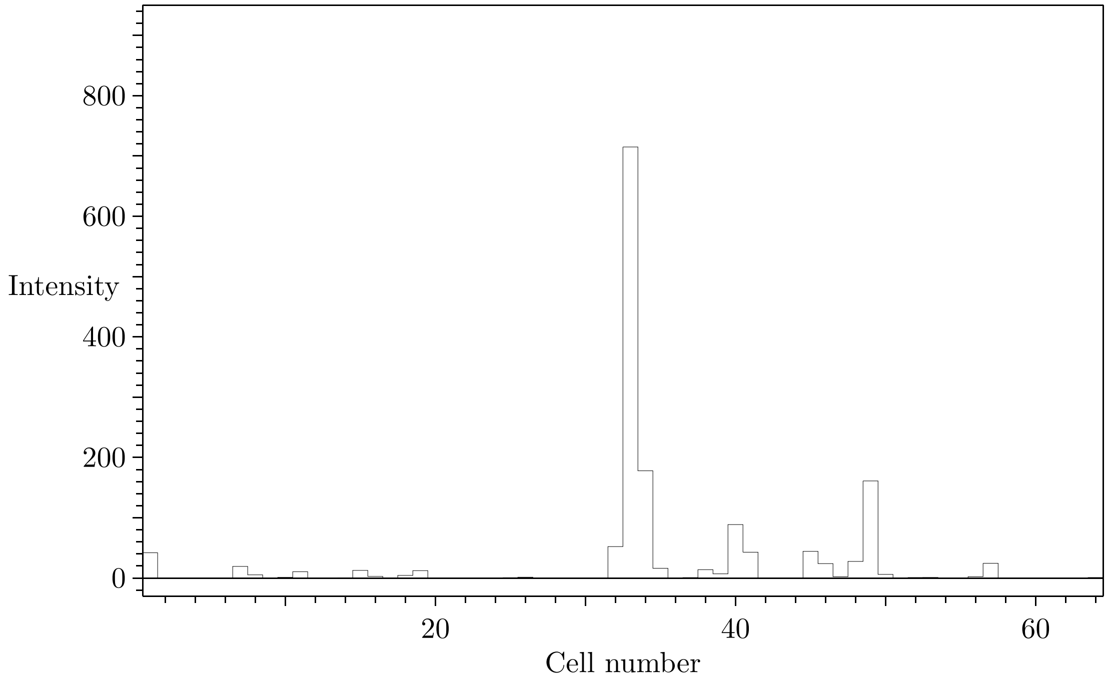

Figure 11.3: Hidden-space distribution $\widehat{\boldsymbol{h}}$ from which $\widehat{\boldsymbol{f}}$ in Figure 11.2 was constructed.

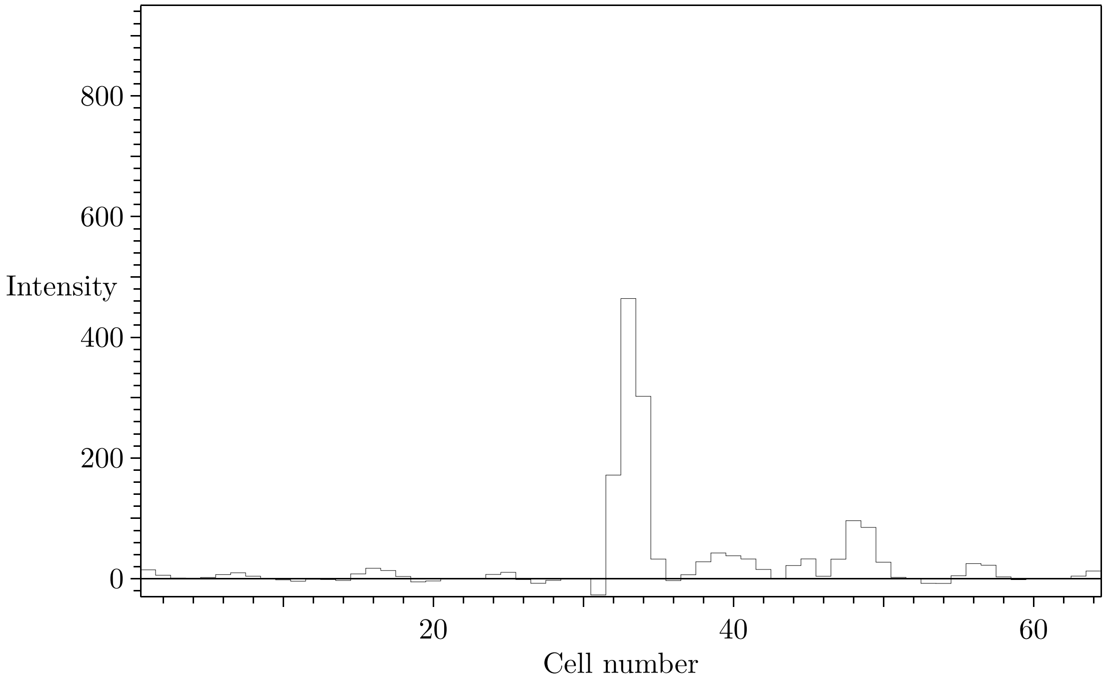

Figure 11.4: `MOVIE5` sample from the posterior cloud in visible space.

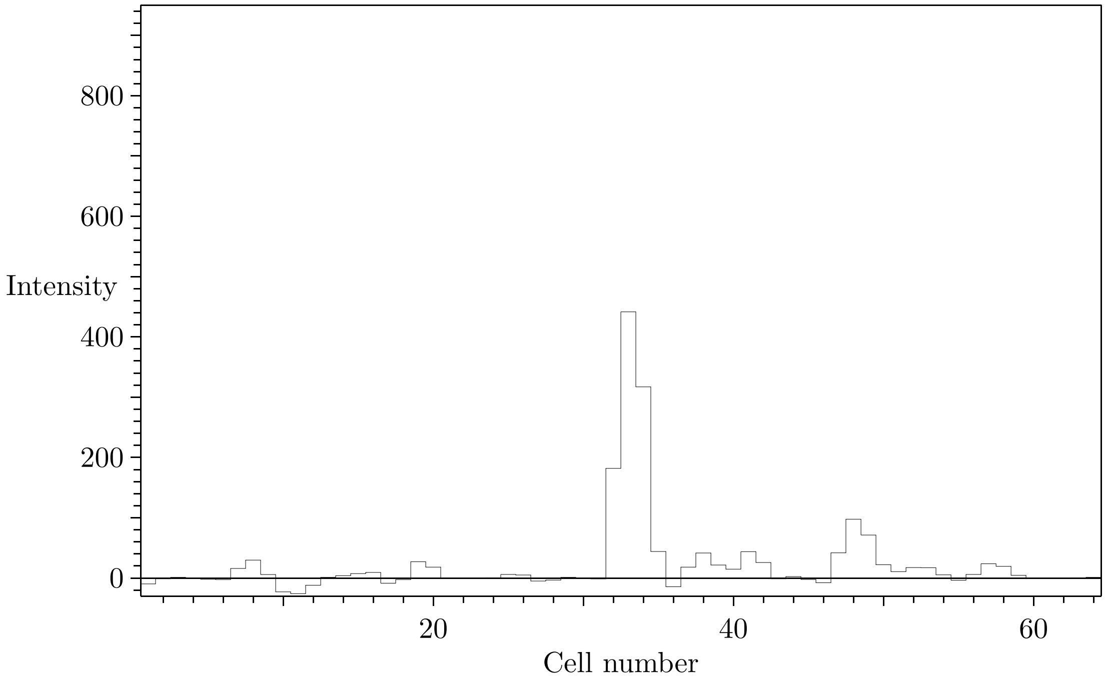

Figure 11.5: `MOVIE5` sample from the posterior cloud in visible space.

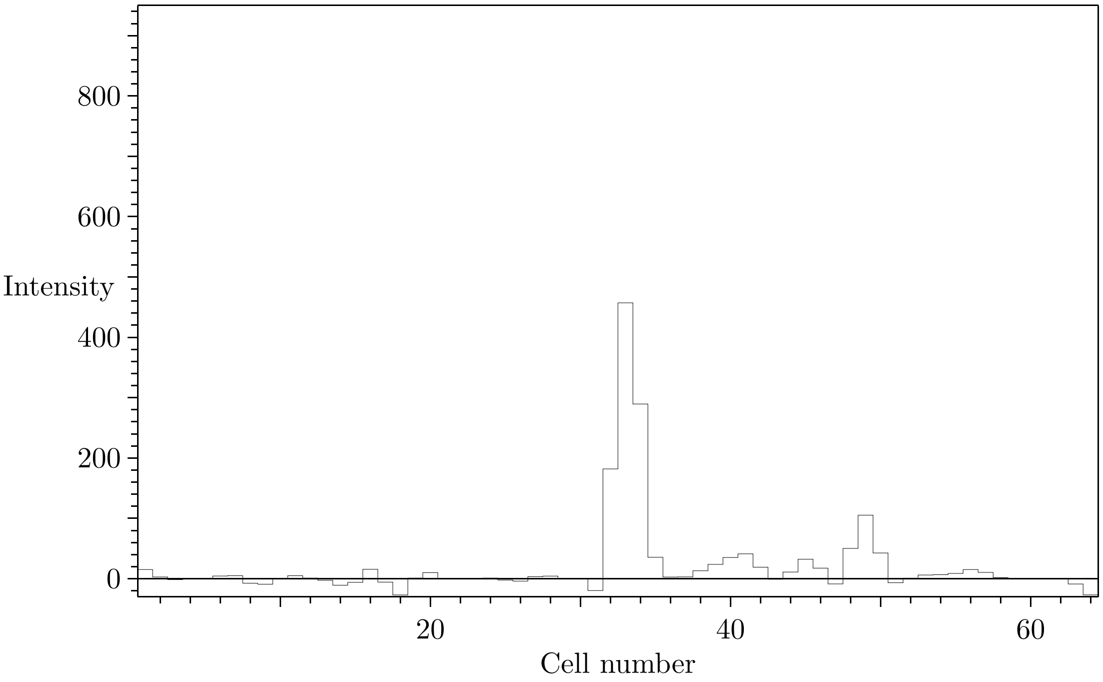

Figure 11.6: `MOVIE5` sample from the posterior cloud in visible space.

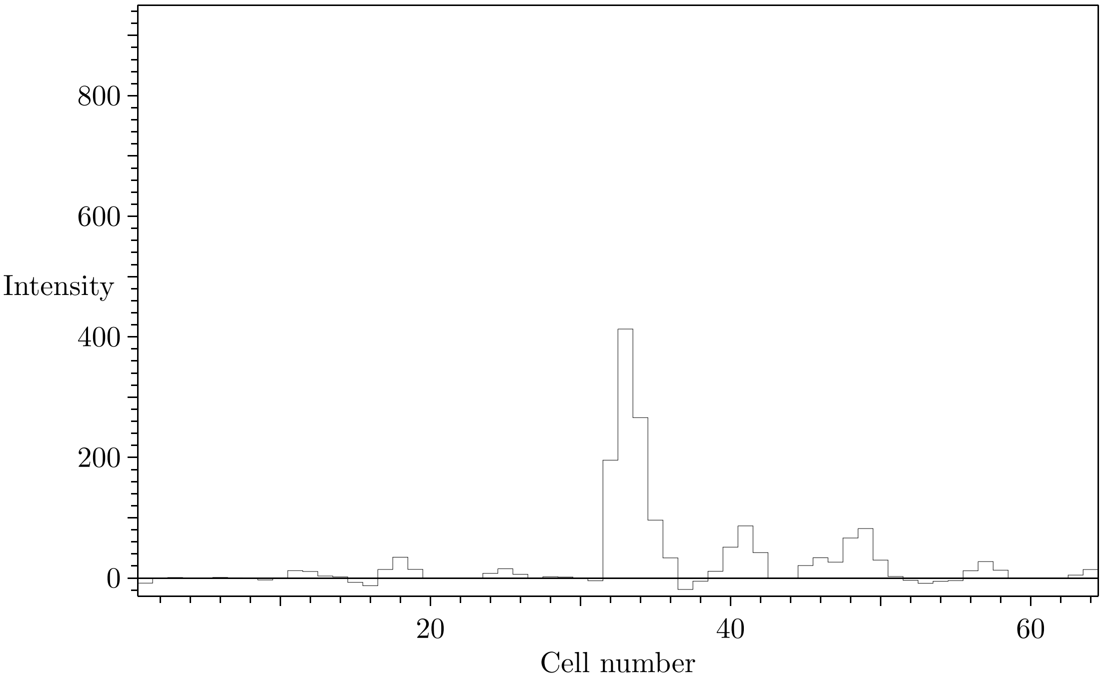

Figure 11.7: `MOVIE5` sample from the posterior cloud in visible space.

Finally, the use of `MASK5` is illustrated to enable specific linear features of the reconstruction to be quantified. Some results are given in Table 11.1.

Cells 32 to 34 contain the major peak, estimated as 891 ± 37. A little more accuracy is obtained by incorporating the nearest neighbours (956 ± 30). Although the central reconstruction shows some structure to the left of this peak, the `MOVIE5` samples indicate that it is not well-defined. This is confirmed by `MASK5`, which demonstrates that it is scarcely significant (112 ± 53). Localised structure to the left, as in cells 6 to 8, is even less significant (23 ± 26). Of course, this relatively large quoted error does not imply that the total quantity in these first three cells might be negative: it represents the curvature at the maximum of the probability cloud, and the cloud necessarily cuts off at zero intensity.

The second peak, in cells 48 to 50, has much the same absolute error as the first (187 ± 35 alone, 202 ± 30 with neighbours), and again there is no significant evidence for structure beyond it (29 ± 34). Between the two major peaks, there is evidence (219 ± 42) of some broad structure, although the precise form varies from one sample to another. There might be a barely-significant peak (62 ± 35) next to an insignificant valley (0 ± 1). Combining these two yields (62 ± 35), with almost the same quoted error (actually fractionally smaller) as on the peak alone: estimates of fluxes in different domains are correlated. All these estimates seem entirely reasonable in the light of the data (Figure 11.1), and serve to confirm the initial visual assessment of the analysis gained from $\widehat{\boldsymbol{f}}$ and the `MOVIE5` samples.

These examples of quantification are only a simple illustration of the use of `MASK5`. More sophisticated masks can be constructed to estimate uncertainties in the position or width of specific features. For features which cannot be expressed or approximated by simple masks, such as medians or maxima, marginal posterior probability densities can be obtained using `MOVIE5` to generate samples for a Monte Carlo analysis. Of course, it will generally be quicker to use `MASK5` to estimate the uncertainty of any specified linear feature.

Table 11.1: Results of `MASK5` quantification of features of $\widehat{\boldsymbol{f}}$ in Figure 11.2.

Left cell	Right cell	Estimate	Description
32	34	890.77 ± 37.46	First major peak
31	35	956.26 ± 30.44	First major peak neighbourhood
1	30	112.25 ± 53.37	Left of first major peak
6	8	23.16 ± 25.77	Localised structure
48	50	186.54 ± 34.50	Second major peak
47	51	202.25 ± 29.74	Second major peak neighbourhood
52	64	29.29 ± 33.80	Right of second major peak
36	46	219.10 ± 41.65	Broad structure
44	46	62.46 ± 34.72	Minor peak
43	43	0.01 ± 1.28	Minor valley
43	46	62.48 ± 34.69	Minor peak and valley

11.2 Post script

The TOY simulation was actually produced from the distribution shown in Figure 11.8—but it might not have been! When attempting to assess the performance of an algorithm by its recovery of a simulation, beware the fixed-point theorem, which implies that under very weak conditions, *any* reconstruction algorithm, no matter how bizarre, will work perfectly for *something*.

The MaxEnt quantified results are compared with the "truth" in Table 11.2. The MaxEnt error bars are seen to be quite reasonable, with the exception of the penultimate value, 0.01 ± 1.28, for which the "true" value was 20. This overly small quoted error, on a low value of $\widehat{f}$, must reflect a local failure of the Gaussian approximation to $\Pr(\boldsymbol{f})$.

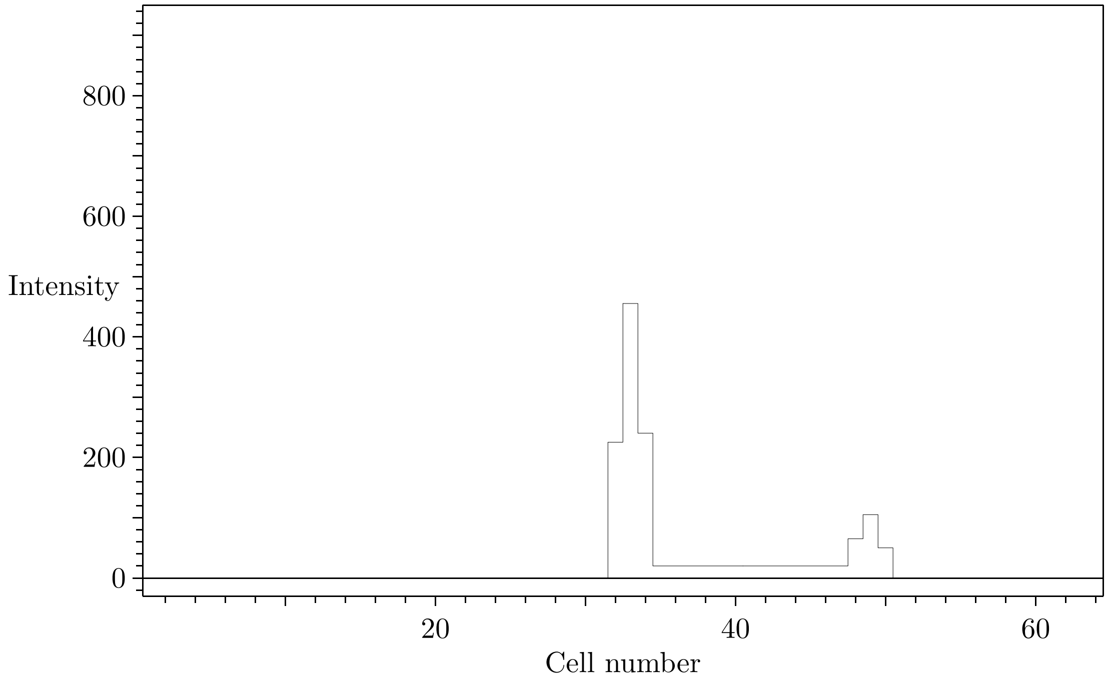

Figure 11.8: The true simulation $\boldsymbol{f}$ for Figures 11.1, 11.2 and 11.3.

Table 11.2: Comparison of estimated and true values for the simulation of Figure 11.2.

Left cell	Right cell	Estimate	True simulation
32	34	890.77 ± 37.46	920
31	35	956.26 ± 30.44	940
1	30	112.25 ± 53.37	0
6	8	23.16 ± 25.77	0
48	50	186.54 ± 34.50	220
47	51	202.25 ± 29.74	240
52	64	29.29 ± 33.80	0
36	46	219.10 ± 41.65	220
44	46	62.46 ± 34.72	60
43	43	0.01 ± 1.28	20
43	46	62.48 ± 34.69	80

Chapter 12

Helpful hints

As with other sophisticated systems, a "folklore" of useful advice continues to build up around the `MemSys` programs.

12.1 Multiple maxima of α

Perhaps most important is that the "best" (i.e., most probable) α may not be unique. As α decreases along the maximum entropy trajectory, there may be more than one local maximum of $\Pr(\boldsymbol{D} \mid \alpha)$, the natural logarithm of which is printed as the diagnostic `LogProb`. Subroutine `MEM5` is designed to give a zero return code at the first such maximum to be encountered. Indeed, `MEM5` will give a return code of zero right at the beginning if the initial default distribution $\boldsymbol{m}$ is already sufficiently close to the data ($\Omega \geq 1.0$).

However, there may be a yet more probable value of α further down the trajectory. The only way of finding out is to force `MEM5` to iterate further down by imposing a suitably low temporary target value `AIM` until `LogProb` starts to increase again (if it does). Then `AIM` can be reset to the desired value (usually 1.0) and `MEM5` should iterate towards the next local maximum.

12.2 Tolerance variable `UTOL`

It can be tempting to try to attain "more accuracy" by reducing the tolerance variable `UTOL`. However, a value of, say, 0.01 ensures that when the stopping is satisfied the probability cloud $\Pr(\boldsymbol{h} \mid \boldsymbol{D})$ estimated by the program overlaps the "true" cloud to within a cross-entropy H of less than 0.01. Actually, the overlap is likely to be considerably more accurate than this, because each successive iterate of `MEM5` attempts to reduce still further the difference between the estimated and "true" clouds. Even in the worst case, an integral $\rho = \widehat{\boldsymbol{h}}^T \boldsymbol{p}$ inferred from the computed cloud should be in error by only about $(2H)^{1/2} \approx 0.14$ of its quoted standard deviation $\delta\rho$. Any greater precision than this merely involves refining the estimates to within a fraction of a standard deviation. Indeed, making `UTOL` unduly small is actually harmful, because it imposes unnecessarily tight requirements on the conjugate gradient algorithms within the package. These will require more (expensive) transforms, possibly to the extent that the internal iteration limits are reached. If that happens, one or more of the return codes code_0, code_1, code_5, code_6 will be non-zero, and the user may be misled into thinking that the program has failed.

The exception to this, when "more accuracy" is truly needed, is when values of Evidence for different variable settings are being used as $\Pr(\boldsymbol{D} \mid \text{variable})$ in order to infer the most probable value of the variable in question. Here, one requires Evidence to be a smooth function of the

variable, in order to locate the maximum. Even here, though, the efficient way of proceeding is to start off with `NRAND` = 1 and `UTOL` reasonably large (perhaps even up to the allowed limit of 1.0), and then do a few more iterations at the end with a smaller value of `UTOL`, and perhaps a larger `NRAND`, until the value of Evidence settles down.

12.3 Internal iteration limits

Unusually difficult problems may hit internal iteration limits even when `UTOL` is relatively large. If this problem persists and causes difficulty, the `PARAMETER LMAX` in the `MemSys INCLUDE` file may be increased from its installation value (usually 30). This is not generally recommended because of the extra expense which may be incurred, and because accumulated rounding errors may detract from the potential extra accuracy.

12.4 Arithmetic accuracy

The accuracy of all calculations within `MemSys5` is commensurate with the arithmetic precision of the transform operations, so that ordinary `REAL` precision is adequate to deal with almost all practical data. In exceptional cases, with data of extreme accuracy, it may be necessary to go to double precision. This is effected by editing " `REAL` " to " `DOUBLE PRECISION` " throughout all the source code, including transforms and the `INCLUDE` file, with explicit spaces so that the variable name `NREALS` is preserved. A particularly strict compiler might also require floating-point formats to change from "`E`" to "`D`". Such formats are to be found in "`WRITE (INFO,'(...E...)')...`" statements. At the same time, the `PARAMETER LMAX` in the `MemSys INCLUDE` file should be increased, say to 50.

12.5 Combination of transforms

Within the `MemSys5` package, the application of $\boldsymbol{C}$ to a vector by `ICF` is almost always followed immediately by an application by `OPUS` of $\boldsymbol{R}$ to the result, and no use is made of the intermediate visible-space vector. The only exceptions to this are in the construction of $\widehat{\boldsymbol{f}}$ from $\widehat{\boldsymbol{h}}$ at the end of each `MEM5` iterate and the construction of the visible sample from $\boldsymbol{v} = \widehat{\boldsymbol{h}} + \Delta\boldsymbol{h}$ at the end of each `MOVIE5` call. Similarly, the application of $\boldsymbol{R}^T$ to a vector is always followed immediately by the application of $\boldsymbol{C}^T$ to the result, and the intermediate vector is not used.

In some cases, it may be computationally cheaper to apply the combined $\boldsymbol{R}\,\boldsymbol{C}$ transform in one operation than to apply the individual $\boldsymbol{C}$ and $\boldsymbol{R}$ transforms separately. Similarly for the transpose operation, it may be cheaper to apply $\boldsymbol{C}^T\boldsymbol{R}^T$ than to apply $\boldsymbol{R}^T$ and $\boldsymbol{C}^T$ separately. If this is so, the user should supply the combined forward operator $\boldsymbol{R}\,\boldsymbol{C}$ as *either* `ICF` *or* `OPUS`, and an identity operator as the other. Likewise, the combined transpose operator $\boldsymbol{C}^T\boldsymbol{R}^T$ should be supplied as *either* `TRICF` *or* `TROPUS`, and an identity operator as the other. It may be convenient to overlay areas ⟨2⟩ and ⟨`11`⟩ (if `OPUS` applies $\boldsymbol{R}\,\boldsymbol{C}$) or areas ⟨`11`⟩ and ⟨25⟩ (if `ICF` applies $\boldsymbol{R}\,\boldsymbol{C}$) so that no actual operation is required to apply the identity.

If the transforms are combined in this way, however, then the vectors supplied in area ⟨`11`⟩ on return from `MEM5` and `MOVIE5` will not be the correct visible-space quantities, and the recovery of the correct visible-space quantities from the hidden-space distributions becomes the user's responsibility.

Bibliography

[1] M. K. Charter. Drug absorption in man, and its measurement by MaxEnt. In Paul F. Fougère, editor, *Maximum Entropy and Bayesian Methods, Dartmouth College 1989*, pages 325–339, Dordrecht, 1990. Kluwer. ISBN 0–7923–0928–6.

[2] M. K. Charter. Quantifying drug absorption. In Grandy and Schick [4], pages 245–252. ISBN 0–7923–1140–X.

[3] R. T. Cox. Probability, frequency and reasonable expectation. *Am. J. Physics*, 14(1):1–13, 1946.

[4] W. T. Grandy, Jr. and L. H. Schick, editors. *Maximum Entropy and Bayesian Methods, Laramie, Wyoming, 1990*, Dordrecht, 1991. Kluwer. ISBN 0–7923–1140–X.

[5] S. F. Gull. Developments in maximum entropy data analysis. In Skilling [15], pages 53–71. ISBN 0–7923–0224–9.

[6] S. F. Gull and G. J. Daniell. Image reconstruction from incomplete and noisy data. *Nature*, 272:686–690, April 1978.

[7] S. F. Gull and J. Skilling. The maximum entropy method. In J. A. Roberts, editor, *Indirect Imaging*. Cambridge Univ. Press, 1984.

[8] S. F. Gull and J. Skilling. Maximum entropy method in image processing. *IEE Proc.*, 131(F):646–659, 1984.

[9] E. T. Jaynes. Where do we stand on maximum entropy? In R. D. Rosenkrantz, editor, *E.T. Jaynes: Papers on Probability, Statistics and Statistical Physics*, chapter 10, pages 210–314. D. Reidel, Dordrecht, 1983. Originally published in 1978 in the Proceedings of a Symposium on 'The Maximum-Entropy Formalism' held at M.I.T. in May 1978.

[10] H. Jeffreys. *Theory of Probability.* Oxford Univ. Press, Oxford, third edition, 1985. ISBN 0–19–853–193–1.

[11] R. D. Levine. Geometry in classical statistical thermodynamics. *J. Chem. Phys.*, 84:910–916, 1986.

[12] C. C. Rodríguez. The metrics induced by the Kullback number. In Skilling [15], pages 415–422. ISBN 0–7923–0224–9.

[13] J. E. Shore and R. W. Johnson. Axiomatic derivation of the principle of maximum entropy and the principle of minimum cross-entropy. *IEEE Trans. Info. Theory*, IT–26(1):26–37, January 1980.

[14] J. Skilling. Classic maximum entropy. In *Maximum Entropy and Bayesian Methods, Cambridge 1988* [15], pages 45–52. ISBN 0–7923–0224–9.

[15] J. Skilling, editor. *Maximum Entropy and Bayesian Methods, Cambridge 1988*, Dordrecht, 1989. Kluwer. ISBN 0–7923–0224–9.

[16] J. Skilling. On parameter estimation and quantified MaxEnt. In Grandy and Schick [4], pages 267–273. ISBN 0–7923–1140–X.

[17] J. Skilling and S. F. Gull. Bayesian maximum entropy. In A. Possolo, editor, *Proc. AMS–IMS–SIAM Conference on Spatial Statistics and Imaging, Bowdoin College, Maine, 1988*, 1989.

[18] J. Skilling, D. R. T. Robinson, and S. F. Gull. Probabilistic displays. In Grandy and Schick [4], pages 365–368. ISBN 0–7923–1140–X.

[19] Y. Tikochinsky, N. Z. Tishby, and R. D. Levine. Consistent inference of probabilities for reproducible experiments. *Phys. Rev. Lett.*, 52:1357–1360, 1984.

Appendix A

User-supplied routines

The following subroutines which are called by the `MemSys5` package must be supplied by the user. Examples of them are provided in the `TOY` demonstration program.

`UINFO` Handle diagnostic messages from the package. See page 59.

`USAVE` Preserve arrays of `INTEGER`s and `REAL`s containing the package's internal variables. See page 61.

`UREST` Retrieve the arrays of `INTEGER`s and `REAL`s preserved by `USAVE`. See page 61.

`UFETCH` Read a block of an area from a direct-access disc file. See page 52.

`USTORE` Write a block of an area to a direct-access disc file. See page 52.

`ICF` Transform from hidden space to visible space, giving correlations in visible-space distribution. See page 57.

`TRICF` Transform from visible to hidden space, applying transpose of `ICF` transform. See page 57.

`OPUS` Transform from visible space to data space, giving differential response. See page 55.

`TROPUS` Transform from data space to visible space, applying transpose of `OPUS` transform. See page 55.

`UMEMX` Calculate nonlinearity in differential response (called only if `METHOD(3)` $= 1$). See page 56.

Appendix B

Historic maximum entropy

It has been shown in part I of this manual that the maximum entropy analysis implemented in the `MemSys5` package follows inevitably from the basic laws of probability theory as applied to positive additive distributions, and the whole process should be considered as an application of Bayesian inference. Nonetheless, maximum entropy can be used as an *ad hoc* regularisation technique, in which the experimental data are forced into a definitive "testable" form which rejects outright all but a "feasible" set of $\boldsymbol{h}$. The Principle of Maximum Entropy is then applied directly by selecting that surviving feasible $\boldsymbol{h}$ which has greatest entropy $\mathcal{S}$.

The misfit between the observed data $\boldsymbol{D}$ and the predicted data $\boldsymbol{F}(\boldsymbol{h})$ may be quantified in the usual way with a χ^2 statistic:

$$\chi^2(\boldsymbol{h}) = (\boldsymbol{D} - \boldsymbol{F}(\boldsymbol{h}))^T [\boldsymbol{\sigma}^{-2}]\, (\boldsymbol{D} - \boldsymbol{F}(\boldsymbol{h})).$$

On average, χ^2 might be expected to take a value not much greater than its formal expectation over possible data, namely

$$\chi^2(\boldsymbol{h}) \leq N,$$

which corresponds to the data being, on average, one standard deviation away from the reconstruction. This "discrepancy principle" can be used as a testable constraint to define the feasible distributions $\boldsymbol{h}$.

In the event that $\boldsymbol{h}$ is positive and additive, entropic arguments apply, and the "best" feasible $\boldsymbol{h}$ can be found by

"maximising $\mathcal{S}(\boldsymbol{h})$ over the constraint $\chi^2(\boldsymbol{h}) = N$ ".

This is illustrated in Figure B.1. The "historic" maximum entropy reconstruction is that distribution $\boldsymbol{h} = (4.36, 3.00)$ which occurs where the trajectory cuts into the shaded region $\chi^2(\boldsymbol{h}) \leq N$.

We call the "$\chi^2 = N$" technique "historic maximum entropy" after its use by Frieden (1972), Gull and Daniell (1978), Gull and Skilling (1984b) and others. Although such reconstructions $\boldsymbol{h}$ are often much clearer and more informative than those obtained with cruder techniques, historic maximum entropy is not Bayesian, and hence imperfect. The constraint $\chi^2(\boldsymbol{h}) = N$ is only an approximation to the true likelihood $\Pr(\boldsymbol{D} \mid \boldsymbol{h})$, no single selected $\boldsymbol{h}$ can fully represent the posterior probability $\Pr(\boldsymbol{h} \mid \boldsymbol{D})$ which theory demands, and it is difficult to define the number N of fully independent data in a suitably invariant manner. A further difficulty arises with Poisson data, for which the formal expectation over possible data of the log(likelihood) analogue of χ^2 is not constant, but varies with the PAD $\boldsymbol{h}$.

Finally, it is well known that the discrepancy principle gives systematically underfitting reconstructions. The reason is that the χ^2 statistic between $\boldsymbol{D}$ and $\boldsymbol{F}(\boldsymbol{h})$ will indeed average to N if $\boldsymbol{h}$ is

the “truth”. The truth, however, is unattainable, and the computed $\boldsymbol{h}$ will necessarily be biassed towards the data, so that the misfit is reduced. Accordingly, “$\chi^2 = N$” is too pessimistic. The classic Bayesian analysis confirms this and quantifies the expected misfit in terms of good and bad degrees of freedom.

Nevertheless, for reasons of compatibility, historic maximum entropy is retained within `MemSys5` as the “`METHOD(0)` = 4” option.

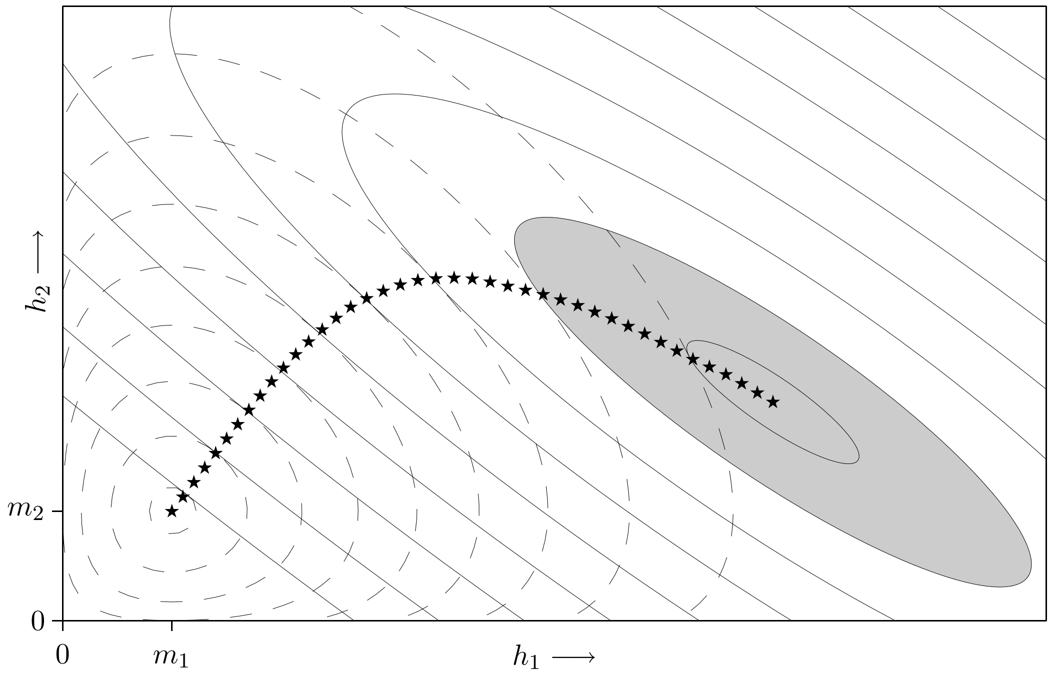

Figure B.1: Historic maximum entropy.

Appendix C

Reserved names

The `MemSys5` package contains the following globally-visible symbols:

MADD	MASK5	MCHECK	MCLEAR
MDIV	MDOT	MECOPY	MEFLAG
MEINFO	MEINIT	MEM5	MEMCGD
MEMCGH	MEMCHI	MEMCOM	MEMDET
MEMDOT	MEMENT	MEMGET	MEMICF
MEMLA	MEMLA0	MEMLAR	MEMLAW
MEMLB	MEMOP	MEMPOI	MEMSET
MEMSMA	MEMSMD	MEMTR	MEMTRQ
MENT1	MENT2	MENT3	MENT4
MENT51	MENT52	MEPRB0	MEPROB
MEPROJ	MEREST	MESAVE	MESCAL
MESMUL	MESURE	MESWAP	METEST
MEVMUL	MEVRND	MEXP	MEZERO
MFETCH	MFILL	MLOG	MMEMX
MMOV	MMUL	MOVIE5	MRAND
MRECIP	MSADD	MSIGN	MSMUL
MSMULA	MSQRT	MSUB	MSUM
MSWAP	MTRICF	MWHILE	

and from the library of storage-handling and vector routines:

VADD	VDIV	VDOT	VEXP
VFETCH	VFILL	VLOG	VMEMX
VMOV	VMUL	VPOINT	VRAND
VRAND0	VRAND1	VRAND2	VRANDC
VRECIP	VSADD	VSIGN	VSMUL
VSMULA	VSQRT	VSTACK	VSTORE
VSUB	VSUM	VSWAP	

These reserved names should be not used for other subroutines, functions, or `COMMON` blocks.

Appendix D

Changes from MemSys2 to MemSys3

Apart from the obvious improvements in quantification and algorithmic power, the principal changes from the earlier MemSys2 maximum entropy program are:-

(a) Incorporation of positive/negative distributions.

(b) Incorporation of Poisson statistics.

(c) Withdrawal of the Kramers–von Mises "exact-error-fitting" option as inconsistent with correct theory.

(d) Withdrawal of support for sequential-access disc files (because of the difficulty of updating in place).

(e) Withdrawal of user-defined weights in the individual cells, which are unnecessary, given that h_j is defined as the quantity in cell j.

(f) Accuracies defined as $1/\sigma$, not $2/\sigma^2$

(g) Altered format of common block MECOMP and addition of a control block MECOML.

(h) All variables within MemSys3 are explicitly typed, and the common blocks specified in the "INCLUDE" files must adhere to this convention.

(i) Storage limits, such as in MECOMS, can be declared as PARAMETERs, so that potential array bound violations can be checked at runtime.

(j) Only the lowest, vector library, routines need be aware of the physical storage locations, because all pointers are passed explicitly as integers: this aids portability.

Appendix E

Changes from MemSys3 to MemSys5

The principal changes from the earlier MemSys3 package are further algorithmic power, and:–

(a) Generation of random samples from the posterior probability density $\Pr(\boldsymbol{h} \mid \boldsymbol{D})$.

(b) Estimates of the numerical uncertainties in G and $\log \Pr(\boldsymbol{D})$ are provided.

(c) Different usage of the scalars DEF and ACC for setting 'flat' defaults and constant accuracies.

(d) Output scalars such as ALPHA and SCALE no longer need to be preserved by the user between calls to the package.

(e) Intrinsic correlation functions (ICFs) are supported explicitly.

(f) Fermi–Dirac and quadratic entropy have been added to the existing standard and positive/negative options.

(g) The vector arithmetic workspace ST is supplied as an argument to the major subroutines of the package, and also to the user-supplied transform routines.

(h) Revised storage management, so that the externally-visible COMMON blocks have been withdrawn.

(i) A separate subroutine is used to set the MemSys5 control variables METHOD, AIM, RATE, etc.

(j) All diagnostic output is directed through calls to a user-supplied diagnostic handler, so that LEVEL and IOUT are no longer required inside the package.

(k) A 'save/restore' facility has been added, to save the internal state of the package in external storage (perhaps on disc), whence it can later be restored to regenerate the internal state exactly.

(l) The area-copying routines MEMJ and MEMK have been withdrawn, and replaced with a single routine MECOPY.

(m) The user-supplied subroutine MEMEX for specifying nonlinear response has been re-named UMEMX.

(n) The compiled object size is smaller.

Index

α, *see* regularisation constant
β distance limit, **27**, 28, 35, 38, 69
χ^2, 16, 30, 65, 68, 78, 93–94

`ACC` argument
  of `MEMGET`, 66
  of `MEMSET`, 46, 61, 65, **66**, 67, 73, 99
accuracies, 46, 61, 66, 67, 70, 71, 73, 97
addressing, 49
  disc, 51–52, 60, 61
  memory, 45, 50–52, 60, 61
advanced maximum entropy, 25
`AIM` argument
  of `MEMGET`, 66
  of `MEMSET`, 62, 65, **66**, 68, 73, 87, 99
algorithm assessment, 84
algorithmic performance, 84, 97, 99
`ALPHA` argument, 66–69, 99
`AMEAN` argument, **71**
areas, 43, 45–47, 50–52, 66–71
arguments
  of `ICF`, 57
  of `MASK5`, 71
  of `MEM5`, 66–69
  of `MOVIE5`, 69–71
  of `MEMGET`, 66
  of `MEMSET`, 65–66
  of `OPUS`, 55
  of `TRICF`, 57
  of `TROPUS`, 55
  of `UMEMX`, 56
array bounds, 97
assignment of density, 13–15
automatic noise scaling, 30–31, 35, 62, 65, 75, 78

bad measurements, 28, 31, 68, 78, 94
Bayes' theorem, 12, 30
Bayesian calculus, 11
Bayesian formulation, 17, 93

buffers, **51**, 52, 53, 63

C language, 7
central reconstruction, 26, 31, 38, 43, 75, 83
`CHISQ` argument, 60, 66, 67, **68**, 69
classic maximum entropy, 17–24, 27, 30, 35, 36, 39, 62, 65, 66, 68, 69
  example, 75–83
cloud, **17**, 19–25, 27–28, 30, 31, 36, 38, 39, 43, 66, 67, 69, 70, 78, 79, 81–83, 87
compatibility, 55, 94
conditional probability, 12, 17, 19, 25, 30
conjugate gradient, 36–37, 66, 70, 78, 87
consistent coding, 44, 60, 73, 78
constraint on $\sum h$, 34, 65
continuum limit, 14, 24, 26, 29
control, 43
convolution, 73
cross-entropy, 27, 87

data, 7, 11–14, 16–27, 30–31, 36, 38, 43–45, 55, 63, 66, 73, 74, 78, 79, 83, 87, 88, 93
data space, 30, 43, 45, 47, 49, 55
  algorithm, 38–39
deconvolution, 7, 73, 78
`DEF` argument
  of `MEMGET`, 66
  of `MEMSET`, 46, 61, 65, **66**, 67, 73, 99
default model, **14**, 19, 25, 46, 47, 66, 73, 87, 99
degeneracy, 15
determinant of matrix, 36–38
diagnostics, 49, 59–60, 63, 67, 69, 75–79, 87, 99
direct-access disc file, 51
distance limit, 27, 62, 66, 75, 78
distribution, 13–15, **25**
  default, 87
  Fermi–Dirac, 34

hidden-space, *see* hidden space, distribution
visible-space, *see* visible space, distribution
distributions, 7
`DOUBLE PRECISION`, 62, 71, 88

eigenvalues, 18
entropic prior, 13–15, 17, 29
entropy, **14**, 17, 19, 28, 33, 35, 38, 65, 67, 78
Fermi–Dirac, *see* Fermi–Dirac entropy
positive/negative, *see* positive/negative entropy
quadratic, *see* quadratic entropy
standard, 65
`ERR` argument, 60
`ERRHI` argument, **71**
`ERRLO` argument, **71**
error messages, 61–63
evidence, **13**, 18, 19, 30, 38, 43, 62, 66, 75, 78, 87
exact-error-fitting, 97
external storage, 45, 47, 50, 52, 61, 63

fatal error, 61
feasible distribution, 93
Fermi–Dirac entropy, **34**, 65, 73, 99
fixed-point theorem, 84
flowchart, 59, 60
FORTRAN 77, 7, 43, 65, 67, 69, 71

Gaussian approximation, 17–18, 20, 25, 79, 84
Gaussian distribution, 25
Gaussian errors, 12, 15–17, 19, 27, 43, 62, 65, 66, 73, 75
`GDEV` argument, 66, 67, **68**, 69
`GHIGH` argument, 66, 67, **68**, 69
`GLOW` argument, 66, 67, **68**, 69, 78
good measurements, **19**, 28, 31, 38, 43, 68, 69, 78, 94

hardware, 49, 51, 53
hidden space, **29**, 38, 43, 45, 47, 49, 63, 70, 71, 73
algorithm, 35–36, 38
distribution, 16, 19, 43, 45, 57, 69, 79, 80, 88
hints, 87–88
historic maximum entropy, 35, 65, 66, 93–94

I/O, *see* input/output
ICF, *see* intrinsic correlation function
`ICF` subroutine, **57**, 60, 63, 73, 78, 88, 91
number of calls, *see* `NTRANS` argument
image, 13, 29
inference, 7, 11–13, 93
about noise level, 30–31
about the reconstruction, 19, 24–26
routine `MASK5`, 71
initialisation
`MEM5` run, 43, 61, 67
random generator, *see* random generator, initialisation
storage area management, 73
system, *see* system initialisation
input/output, 50–52, 59
intrinsic correlation function, **29**, 33, 57, 63, 73, 79, 99
`ISEED` argument
of `MEMGET`, 66
of `MEMSET`, 65, **66**
of `MEMTRQ`, 60
`ISTAT` argument, 66, 67, **69**, 73, 78
iterative algorithm, 26–29
in data space, *see* data space, algorithm
in hidden space, *see* hidden space, algorithm

joint probability, 12, 17
`JSTAT` argument, **71**

kangaroos, 14, 29
Kramers–von Mises, 97
`KSTAT` argument, **70**

L, **16**, 17
$\mathcal{L}$, 17–20, 26, 28, 30, 35, 38, 68
Lagrange multiplier, **38**, 43, 46, 69, 71, 78
least squares, 34
`LEVEL` argument, 99
likelihood, **12**, 13, 93
Gaussian, *see* Gaussian errors
Poisson, *see* Poisson statistics
local maximum, 19, 87
logical proposition, 11

macroscopic data, 24
mask, 25, 43, 46, 71, 73, 83

MASK5 subroutine, 43, 44, 46, 61, 71, 73, 83, 95
maximum entropy, 7, 14, 25, 29, 33, 34, 38
  classic, *see* classic maximum entropy
  cloud, 20–24
  historic, *see* historic maximum entropy
  principle, *see* principle of maximum entropy
  program, 55, 73
  trajectory, 19, 26, 30, 43, 87
MECOML common block, 97
MECOMP common block, 97
MECOMS common block, 97
MECOPY subroutine, 59, **63**, 95, 99
MEINIT subroutine, 44, **47**, 62, 73, 95
MEM5 subroutine, 43, 44, 46, 60–62, 65–69, 71, 73, 75–83, 87, 88, 95
MEMEX subroutine, 99
MEMGET subroutine, 66
MEMICF subroutine, **63**, 95
MEMJ subroutine, 99
MEMK subroutine, 99
MEMRUN argument, 61, **67**, 69, 71, 73
MEMSET subroutine, 65–66, 71
MemSys2, 97
MemSys3, 97–99
MemSys5, 7, 26, 43, 45, 49, 52, 53, 57, 59, 61–63, 73, 88, 93–95
MEMTRQ subroutine, 44, **60**, 73, 95
MEREST subroutine, **61**, 95
MESAVE subroutine, **61**, 95
MESWAP subroutine, **63**, 95
METHOD argument
  of MEMGET, 66
  of MEMSET, 54, 61, 62, **65**, 66–69, 73, 75, 94, 99
metric tensor, 15
microscopic structure, 24
MLEVEL argument, **59**, 75
mock data, 25, 29, 93
model, 25, 30, 33, 34, 70, 71
monkeys, 15
Monte Carlo, 38, 83
movie, **26**, 43
MOVIE5 subroutine, 43, 44, 46, 61, 69–71, 73, 79–83, 88, 95
MTRICF subroutine, **63**, 71, 73, 95
multiplier, *see* Lagrange multiplier

NCORR argument, 69, **70**
noise, 12, 15, 25, 27, 28, 30–31, 35, 65, 78, 79
  Gaussian, 43, 73
  Poisson, 73
  scaling, 68
nonlinear
  functional, 26, 43
  response, 29, 46, 54, 56, 61, 62, 66, 67, 70, 71
normal statistics, *see* Gaussian errors
NRAND argument
  of MEMGET, 66
  of MEMSET, 62, 65, **66**, 73, 75, 78, 88
NTRANS argument
  MASK5, 71
  MEM5, 66, 67, 69
  MOVIE5, 69, 71

OMEGA argument, 66, 67, **68**, 69, 78
OPUS subroutine, 55–57, 60, 73, 78, 88, 91
  number of calls, *see* NTRANS argument
overall probability, *see* evidence
overlap of clouds, 28, 66, 78, 87
overlay of areas, 47, 53, 55, 88

PAD, *see* positive additive distribution
PDEV argument, 66, 67, **68**, 69
PHIGH argument, 66, 67, **68**, 69, 78
PLOW argument, 66, 67, **68**, 69, 78
pointers, 50, 97
Poisson errors, 12, 43, 61, 62, 65, 67, 93, 97
positive additive distribution, 13–15, 25, 93
positive/negative distribution, 33, 97
positive/negative entropy, 65
posterior inference, 13
posterior probability, 17, 20, 24–26, 28, 31, 36, 39, 43, 70, 79, 83, 93, 99
power spectrum, 13, 29
predicted data, *see* mock data
principle of maximum entropy, 14, 93
prior probability, 13–15
  for α, 18
  for unknown variables, 30
probability calculus, 11–12
proportional error, 24, 29
  in c, 79
proposition, *see* logical proposition

quadratic entropy, 65, 99

random fluctuations, 27
random generator, 53, 60
  initialisation, 53, 65, 66
random vector, 28, 37, 38, 62, 66, 69, 70, 75
  standard deviation, 68, 78
RATE argument
  of MEMGET, 66
  of MEMSET, 62, 65, **66**, 73, 75, 99
REAL precision, 43, 78, 88
regularisation constant, 15, 18–19, 30, 43, 68
reserved names, 95
Residuals, 46
response function, 25, 29, 30, 54, 55, 62, 66, 75
  nonlinear, *see* nonlinear response
restore, *see* save/restore utility
return code, 67, 69–71, 78, 87
  ISTAT, *see* ISTAT argument
  JSTAT, *see* JSTAT argument
  KSTAT, *see* KSTAT argument
rounding error, 53, 60, 71, 88

S, **14**, 17, 33, 34
$\mathcal{S}$, 17–20, 26, 30, 35, 38, 93
S argument, 60, 66, 67, **67**, 69
save/restore utility, 61
SCALE argument, 66, 67, **68**, 69, 78, 99
scientific data analysis, 11, 12
sequential-access disc files, 97
single precision, *see* REAL precision
spatial invariance, 14
special case, 11, 14, 15, 25, 30
ST
  argument
    of ICF, 57
    of MASK5, **71**
    of MECOPY, 63
    of MEM5, **67**
    of MEMICF, 63
    of MEMTRQ, 60
    of MESWAP, 63
    of MOVIE5, 69, **70**
    of MTRICF, 63
    of OPUS, 55
    of TRICF, 57
    of TROPUS, 55
    of UFETCH, 52
    of USTORE, 52
    of VFETCH, 50
    of VSTORE, 50
  vector arithmetic workspace, **45**, 47, 50–53, 55, 63, 99
stopping criterion, **19**, 28, 31, 35, 36, 39, 43, 61, 62, 65, 66, 68, 78, 87
stopping value, **19**, 26, 27, 43
storage requirement, 47
subspace, 36, 38
system initialisation, 44, 47, 73

termination criterion, **27**, 28
TEST argument, 60, 66, 67, **68**, 69, 78
testable information, 14, 93
TOL variable, 62
tolerance
  arithmetic, 62
  user-supplied, 37, 62, 66, 69, 75, 87
TOY program, 7, 73, 75–84
trace of matrix, 37, 38
trajectory, *see* maximum entropy, trajectory
transform
  area, **47**, 49, 63, 88
  routines
    ICF, *see* ICF subroutine
    OPUS, *see* OPUS subroutine
    TRICF, *see* TRICF subroutine
    TROPUS, *see* TROPUS subroutine
transforms, 36, 46, 47, 53–56, 60, 61, 67, 71, 78, 87–88, 99
  consistency of, *see* consistent coding
  number of, *see* NTRANS argument
TRICF subroutine, **57**, 60, 73, 78, 88, 91
  number of calls, *see* NTRANS argument
TROPUS subroutine, 55–57, 60, 73, 78, 88, 91
  number of calls, *see* NTRANS argument
trust region, **27**, 66

UFETCH subroutine, **52**, 91
UINFO subroutine, 59–60, 75, 91
UMEMX subroutine, 54, **56**, 66, 91, 99
uniqueness, 19
  lack of, 19, 87
UREST subroutine, 61, 91
USAVE subroutine, **61**, 91
USTORE subroutine, **52**, 91
UTOL argument
  of MEMGET, 66

of `MEMSET`, 62, 65, **66**, 69, 71, 73, 75, 78, 87, 88

vector arithmetic, 49
library, 49, 53–54
workspace, *see* `ST`, vector arithmetic workspace
vector coding, 36–38
vector space, 25, 29, 49
data, *see* data space
hidden, *see* hidden space
visible, *see* visible space
`VFETCH` subroutine, 45, 50–52, 63, 95
visible space, **29**, 45, 47, 49, 55, 57, 63, 70, 71, 88
distribution, 29, 33, 43, 45, 55, 69
`/VPOINT/ COMMON` block, 50–52, 56, 61, 95
`VRAND` subroutine, 53, 95
`VSTORE` subroutine, 45, 50–52, 63, 95

weights, 97

zero error, 25

What do you think?

We would like your comments on the MemSys5 package, the TOY demonstration program and the Manual. Criticism is welcome, and constructive criticism with suggestions for improvements even more so. The following questions are a guide only, so please comment on other aspects if you wish.

The MemSys5 package

1. How easy do you think the package is to use? ....

2. What features make it easy to use? ....

   ....

3. What features make it difficult to use? ....

   ....

   How do you think these could be improved? ....

   ....

4. What other facilities would you like to see in the package? ....

   ....

5. Do you write your own transform routines (ICF, TRICF, OPUS, TROPUS)? ....

   If so, do you find this easy? ....

   If not, what do you find difficult? ....

   ....

The TOY demonstration program

1. If you ran the demonstration program, how helpful was it in illustrating how to use MemSys5?

   ....

2. What was particularly helpful about it? ....

   ....

3. What was particularly unhelpful about it? ....

   ....

   How do you think this could be improved? ....

   ....

4. What parts (if any) of the demonstration program would you like to see removed, and why?

   ....

5. What other features (if any) of `MemSys5` would you like to see illustrated in the demonstration program? ....

   ....

The `MemSys5` Users' manual

1. How helpful do you find this manual? ....

   ....

2. Which parts do you find most helpful, and why? ....

   ....

3. Which parts do you find least helpful, and why? ....

   ....

4. How could they be improved? ....

   ....

5. How do you find the mathematical content of the manual? ....

   ....

6. What other things (if any) would you like to see included in the manual? ....

   ....

Please return your comments to Maximum Entropy Data Consultants Ltd.,
South Hill, 42 Southgate Street, Bury St. Edmunds, Suffolk IP33 2AZ, United Kingdom.
