# Supplementary material for: A Molecular Ruler for Measuring Quantitative Distance Distributions
Source: PLoS One. 2008 Oct 17;3(10):e3229. doi: 10.1371/journal.pone.0003229 (PMC2566812; doi:10.1371/journal.pone.0003229)
Supplement: Supplementary Materials S2 — MATLAB Data Analysis Scripts (2.42 MB ZIP) [file pone.0003229.s002.zip › Data for PLoS ONE/Instructions_README/README_Data analysis procedure_final.doc]

**Data analysis procedure:**

Buffer subtracted scattering profiles are available as text files and are located in the folder labeled “buffer subtracted profiles”. Profiles are labeled AB (double-label), A (single label A), B (single label B), Un (unlabeled, normalized), Gn (gold, normalized), or Fn (buffer, normalized) followed by a number that corresponds to the length of the DNA duplex. The scattering angle vector, *S*, is labeled sval# (# corresponds to the length of the DNA duplex).

Data analysis scripts are available as MATLAB m-files and are stored in the “scripts” folder (compatible with version 7.3 or higher). To run a script, type “help script_name” to display the usage line. Copy and paste the usage line at the command prompt.

For example, type the following at the command prompt in MATLAB:

>> help tail

The following text will appear:

Usage: aligned=tail(VAR,vidx,TARG,tidx);

for vidx and tidx type the following at the command line: vidx=find(sval#>0.04);

VAR is the variable (AB, A or B profile) that will be scaled

TARG is the target (G) that VAR is scaled to match across the range specified by vidx

To create the variables vidx and tidx, type:

>> vidx = find(sval#>0.04);

>> tidx = find(sval#>0.04);

Copy and paste the usage line:

>> aligned = tail(VAR,vidx,TARG,tidx);

**Step 1:** Guinier extrapolate the gold scattering profile to determine the zero-angle intensity (gui.m). See Mathew-Fenn *et al.*, (2008) *PLoS One* (Methods, data analysis section) for a complete description of the procedure used to scale the unlabeled, buffer, and gold scattering profiles.

**Step 2:** Use tail.m to align AB#, A#, and B# with the normalized gold profile (G#n).

**Step 3:** Optimize scale coefficients for scattering interference profile using gen_Idelta.m.

The optimized profiles are labeled Id# (# corresponds to length of DNA duplex) and are stored as text files in the folder labeled “interference profiles”.

Please note: when scaling the 25bp data, use 2 copies of the A profile instead of one copy of the A profile and one copy of the B profile.

**Step 4:** Decompose the gold autoscattering profile into a volume-weighted gold nanocrystal radius distribution. At the command line type:

>> Rval = 1:100;

Use gen_IqR.m to create basis functions in the distance increment specified by Rval (1 Angstrom). To transform the gold profile using the 'lsqnonneg' function in MATLAB type:

>> PDD# = lsqnonneg(IqR,G#n);

Plot the transform and make sure that the distance distribution does not have any probability at Rval < 2 Angstrom. If probabilities at Rval < 2 Angstrom are present, then subtract off a constant from the gold scattering profile until the profile tends to zero at high scattering angles.

**Step 5:** Create Ic2c0 (gen_Ic2c0.m). At the command line create the distance increment variable, Dval by typing:

>> Dval = 1:200';

Create gold-gold basis functions for each DNA duplex (IqD#, use gen_IqD.m). Load 'mask.txt' at the command line (located in “scripts” folder):

>> load 'mask.txt'

Finally, create the intrinsic correlation function matrix (ICF, gen_ICF.m):

>> ICF = gen_ICF(Dval,mask,multiplier);

Refer to the MemSys5 users manual for a description of the ICF (located in the Instructions_README folder).

**Step 6:** Recover distance distributions using the MEMSYS 5 maximum entropy minimization routine (fms5.m). The transform can be checked quickly using the 'lsqnonneg' function in MATLAB. Create basis functions in 5 Angstrom increments instead of 1 Angstrom when using the 'lsqnonneg' function (gen_IqD.m, at command line type Dval = 1:5:200';). See Results section of Mathew-Fenn *et al.*, (2008) *PLoS One* for an explanation of method used to determine the natural basis resolution.

Basis functions (IqD#, 1 Angstrom increments) and ICF (one for all duplexes) are included in the “files for generating transforms” folder. Error estimates are in the “files for generating transforms” folder (D#_std.txt). The basis functions must be scaled to be smaller than the magnitude of the oscillations in the interference profile. The following scaling should be used at the command line to run fms5.m:

10bp: IqD10/10000;

15bp: IqD15/10000;

20bp: IqD20*1000000;

25bp: IqD25/10000;

30bp: IqD30/10000;

35bp: IqD35/10000;

The jackknife script calls fms5.m and uses a cross‑validation statistic to determine the regularization coefficient. To run the script type:

>> [fMAT,fvec,alphavec] = jacknife(sval#,Id#,D#_std,IqD#,ICF);

An example:

To run the jackknife script with the 10bp data and scaled basis functions, type:

>> [fMAT,fvec,alphavec] = jacknife(sval10,Id10,D10_std,IqD10/10000,ICF);
